# Three-component microwave-assisted synthesis of 3,5-disubstituted pyrazolo[3,4-*d*]pyrimidin-4-ones†

**DOI:** 10.1039/d2ra00980c

**Published:** 2022-03-16

**Authors:** Jia Hui Ng, Edward R. T. Tiekink, Anton V. Dolzhenko

**Affiliations:** School of Pharmacy, Monash University Malaysia Jalan Lagoon Selatan, Bandar Sunway Selangor Darul Ehsan 47500 Malaysia anton.dolzhenko@monash.edu; Research Centre for Crystalline Materials, School of Medical and Life Sciences, Sunway University 5 Jalan Universiti, Bandar Sunway Selangor Darul Ehsan 47500 Malaysia; School of Pharmacy and Biomedical Sciences, Curtin Health Innovation Research Institute, Faculty of Health Sciences, Curtin University GPO Box U1987 Perth Western Australia 6845 Australia

## Abstract

A practical three-component method for the synthesis of pyrazolo[3,4-*d*]pyrimidin-4-ones was developed. The reaction was performed in a one-pot manner under controlled microwave irradiation using easily accessible methyl 5-aminopyrazole-4-carboxylates, trimethyl orthoformate, and primary amines. Under the optimized conditions, challenging substrates, such as N-1 unsubstituted 5-aminopyrazole-4-carboxylates with another substituted amino group in position 3, reacted selectively affording 5-substituted 3-arylamino-1,5-dihydro-4*H*-pyrazolo[3,4-*d*]pyrimidin-4-ones. The reaction tolerated a range of primary amines, including anilines. The advantages of the developed protocol include short reaction time, pot- and step-economy, and convenient chromatography-free product isolation. The structural features of representative products were explored by X-ray crystallography.

## Abstract 2

A practical three-component method for the synthesis of pyrazolo[3,4-*d*]pyrimidin-4-ones was developed.

## Graphical abstract

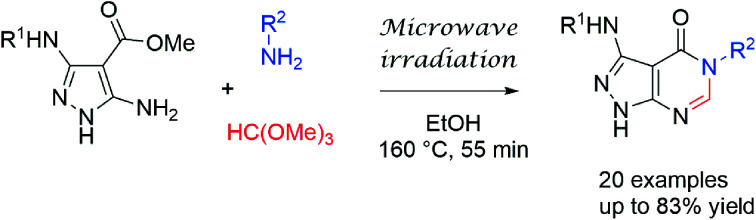

## 1. Introduction

**Fig. 1:**
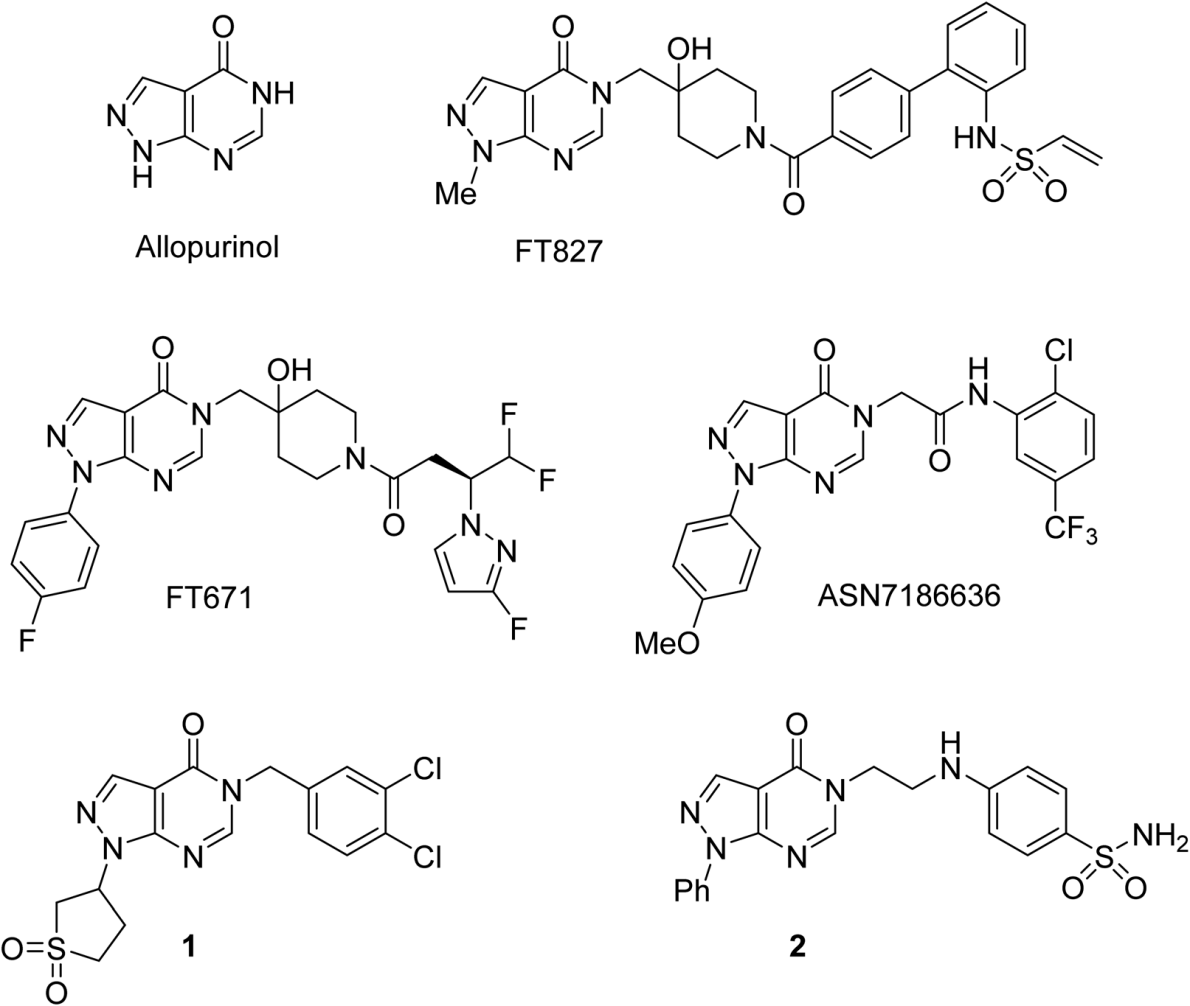
Selected bioactive pyrazolo[3,4-*d*]pyrimidin-4-ones.

The pyrazolo[3,4-*d*]pyrimidin-4-one scaffold has been utilized for bioactive molecule construction for a long time. Without other substituents, this bioisostere of hypoxanthine is known as the anti-gout drug allopurinol (Fig. 1). However, the pharmacological profile of substituted pyrazolo[3,4-*d*]pyrimidin-4-ones is more diverse. For example, pyrazolo[3,4-*d*]pyrimidin-4-ones substituted in position 5 were reported to possess antiviral,^1^ antimicrobial,^2–4^ and anticancer^5–7^ properties. This substitution pattern is shared by highly specific ubiquitin-specific protease 7 inhibitors FT827 and FT671, which were found to be active against cancer *in vitro* and *in vivo*.^8,9^ Recently, ASN7186636 was reported as an inhibitor of another anticancer target, tropomyosin receptor kinase A.^10^ Compound 1 possessing 3,4-dichlorobenzyl substitution in position 5 of the pyrazolo[3,4-*d*]pyrimidin-4-one system was identified as an inhibitor of pro-metastatic protein fascin and served as a starting point for the development of highly potent inhibitors.^11^ Anti-inflammatory and analgesic effects were demonstrated by a potent dual inhibitor cyclooxygenase 2 and inducible nitric oxide synthase, compound 2.^12^

**Scheme 1:**
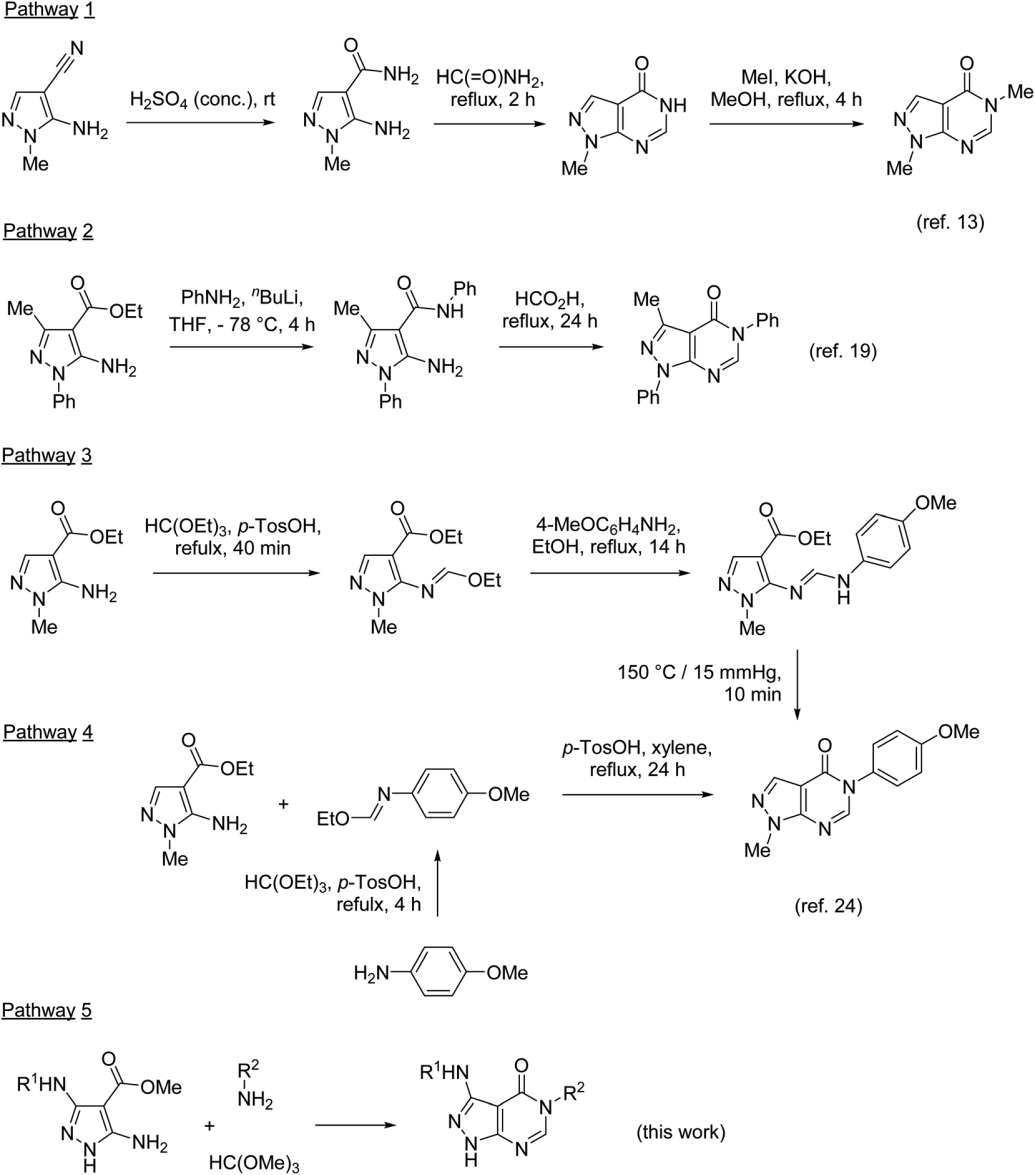
Methods for the synthesis of 5-substituted pyrazolo[3,4-*d*]pyrimidin-4-ones.

The synthesis of 5-substituted pyrazolo[3,4-*d*]pyrimidin-4-ones typically involves the pyrimidinone annulation followed by the *N*-alkylation in position 5. Developed in 1956,^13^ this approach was based on the conversion of 5-amino-1-methylpyrazole-4-nitrile to the corresponding amide and the subsequent pyrimidinone ring closure upon heating in formamide (Scheme 1, pathway 1). Many modifications of this ring closure have been reported. Instead of formamide, formic acid or triethyl orthoformate were used as one-carbon inserting reagents in the cyclization step.^5,14^ Pyrazolo[3,4-*d*]pyrimidin-4-ones were also prepared by heating 5-aminopyrazole-4-nitriles in formic acid^1,6,7,12^ or alkyl 5-aminopyrazole-4-carboxylates in formamide.^15,16^ However, the main drawbacks of this approach remain (1) lack of selectivity in the alkylation of pyrazolo[3,4-*d*]pyrimidin-4-ones unsubstituted at N-1 (ref. 17 and 18) and (2) limitation of groups introduced at N-3 to alkyls.

These limitations were overcome by the preparation of substituted 5-aminopyrazole-4-carboxamides, for example by amidation of esters (pathway 2), followed by the cyclization in the reaction with formic acid.^19^ The pyrimidinone ring formation was also achieved by the treatment of substituted 5-aminopyrazole-4-carboxamides with triethyl orthoformate^20^ or *N*,*N*-dimethylformamide dimethyl acetal (DMF-DMA).^21,22^ The reaction with DMF-DMA is typically performed in two steps with isolation of the corresponding formamidines as intermediates.^2,3,5,23^

Two synthetic approaches to 5-substituted pyrazolo[3,4-*d*]pyrimidin-4-ones were developed by Finlander and Pedersen.^24^ The reaction of ethyl 5-amino-1-methylpyrazole-4-carboxylate with triethyl orthoformate resulted in the formation of the formimidate, which upon the treatment with anisidine transformed to the corresponding formamidine (pathway 3). The thermal cyclization of this formamidine afforded the desired pyrazolo[3,4-*d*]pyrimidin-4-one. This approach, however, was unsuccessful with the N-1 unsubstituted analogue. A more general approach utilizes a different reaction sequence: preparation of formimidate from anisidine and triethyl orthoformate in the first step, followed by the reaction with 5-aminopyrazole-4-carboxylates (pathway 4). In pathway 3, DMF-DMA was also used instead of triethyl orthoformate.^25^

Pathways 2, 3, and 4 are based on similar types of reagents: 5-aminopyrazole-4-carboxylate, primary amines, and triethyl orthoformate (or its synthetic equivalents). However, these pathways are different in the order of steps combining the reagents. Since the outcome of these pathways does not depend on the sequence of their individual reactions, we decided to develop a three-component one-pot methodology introducing the reagents to the reaction mixture together (pathway 5). Multicomponent reactions involving 5-aminopyrazoles and orthoformates often benefit from microwave irradiation.^26–30^ Moreover, it has been reported^31^ that 5-aminopyrazoles react with orthoformates and secondary amines under microwave irradiation affording *N*-pyrazolylformamidines, which resemble intermediates for the synthesis of pyrazolo[3,4-*d*]pyrimidin-4-ones. Therefore, we applied microwave-assisted methodology for the development of our three-component protocol.

## 2. Results and discussion

### 2.1. Synthesis

The synthesis of starting 3-substituted 5-aminopyrazole-4-carboxylates 3 was performed according to the previously reported method.^32^ For the trial reaction and subsequent condition optimization, we used the model reaction of 5-aminopyrazole-4-carboxylate 3a, benzylamine, and trimethyl orthoformate under microwave irradiation in a Discover SP reactor (CEM, USA) (Table 1).

**Table 1:** Optimisation of reaction conditions for the synthesis of 5-benzyl-3-phenylaminopyrazolo[3,4-*d*]pyrimidin-4-one (4a) under microwave irradiation^a^

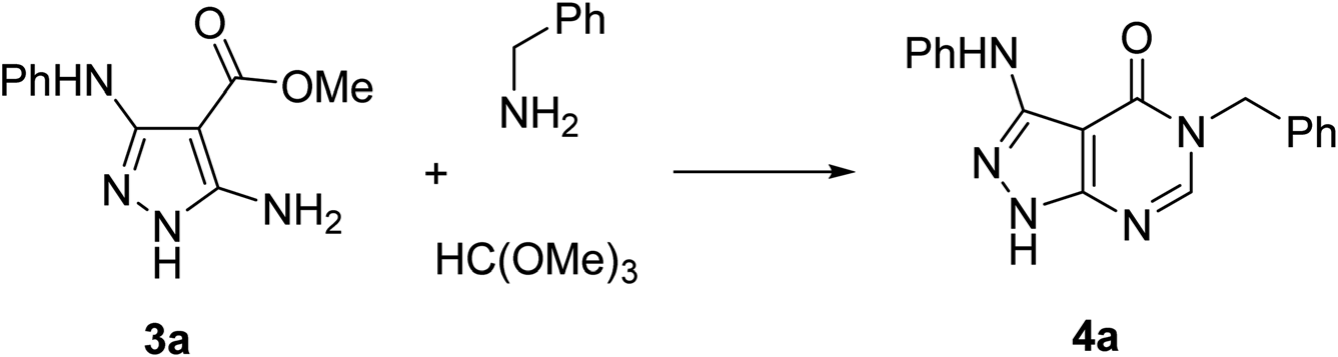				
Entry	Solvent	Temp (°C)	Reaction time (min)	Yield^b^ (%)
1	Toluene	160	35	10
2	MeCN	160	35	66
3	EtOH	160	35	72
4	^*n*^PrOH	160	35	35
5	^*i*^PrOH	160	35	53
6	Eucalyptol	160	35	28
7	2-MeTHF	160	35	12
8	EtOH	160	45	75
9	EtOH	160	55	83
10	EtOH	160	65	75
11	EtOH	150	55	45
12^c^	EtOH	Reflux	4320	Traces^d^
13^e^	EtOH	160	55	27

a The reactions were performed in a Discover SP (CEM, USA) using 3a (1 mmol), trimethyl orthoformate (3 mmol), and benzylamine (3 mmol) in 2 mL of a solvent under a maximal microwave irradiation power of 150 W.

b Isolated yield calculated on the basis of 3a.

c The reaction was performed using conventional heating under reflux.

d The traces are identified in the ^1^H NMR spectrum of the crude reaction mixture.

e The reaction was performed using conventional heating in a Monowave 50 (Anton Paar, Austria).

The reaction at 160 °C for 35 min resulted in the formation of the desired 5-benzyl-3-phenylaminopyrazolo[3,4-*d*]pyrimidin-4-one (4a), which was isolated by simple filtration. Several solvents, including emerging sustainable solvents 2-methyltetrahydrofuran (2-MeTHF) and eucalyptol, were screened under these conditions and the best results were obtained in EtOH (Table 1, entry 3). Further improvements in the yield were achieved by increasing the reaction time to 55 min (entry 9). An attempt to carry out the reaction at a lower temperature (150 °C) resulted in a lower yield (entry 11) while temperatures above 160 °C were precluded by an increase of the pressure above the instrument safety limits. The reaction performed using conventional heating under reflux in EtOH did not afford the desired product even after 3 days (entry 12). We also attempted to carry out this reaction using conventional heating in pressurized vessels resembling the conditions of the reaction under microwave irradiation (entry 13). This reaction in the Monowave 50 (Anton Paar) reactor resulted in the isolation of equally pure 4a but in lower yield (27%).

**Scheme 2:**
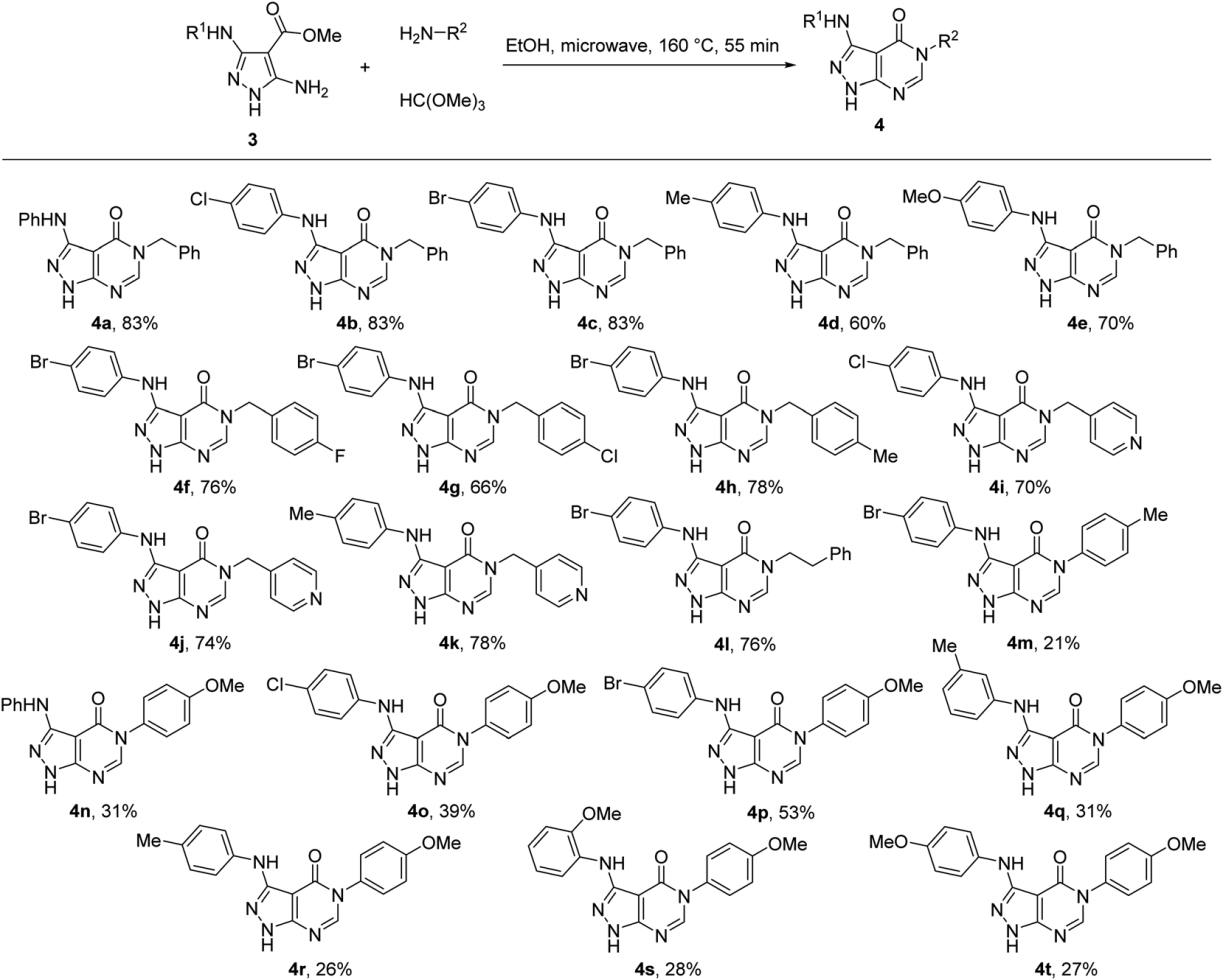
Synthesis of pyrazolo[3,4-*d*]pyrimidin-4-ones 4 under microwave irradiation.

Therefore, for the exploration of the multicomponent reaction scope, we used microwave irradiation at 160 °C for 55 min (Table 1, entry 9) as optimised conditions. Two points of diversity in positions 3 and 5 of pyrazolo[3,4-*d*]pyrimidin-4-ones 4 were generated by different combinations of 5-aminopyrazole-4-carboxylates 3 and primary amines (Scheme 2). Overall, the multicomponent reaction under microwave irradiation was found to be selective and its scope was rather general. The method allowed selective pyrimidine ring annulation on the N-1 unsubstituted 5-aminopyrazole-4-carboxylates 3 and no reactions at pyrazole ring nitrogen atoms or 3-arylamino group were observed. A variety of 3-arylamino substituents on the pyrazole ring of 3 were equally well tolerated. The method optimized for benzylamine was successfully applied for substituted benzylamines and their analogues affording pyrazolo[3,4-*d*]pyrimidin-4-ones 4 in 60–85% yields. However, the yields decreased to 21–53% when aromatic amines were used as substrates.

The structure of the prepared pyrazolo[3,4-*d*]pyrimidin-4-ones 4 was confirmed using NMR spectroscopic data. The carbonyl group signal from the constructed pyrimidinone ring appears in the ^13^C NMR spectra at 156.8–157.2 ppm. The methine group of this heterocyclic ring gives a signal at 150.7–151.3 ppm in the ^13^C NMR spectra and a downfield-shifted singlet at 8.06–8.54 ppm in the ^1^H NMR spectra. This signal in the ^1^H NMR spectra of compounds 4a–k appears ∼0.3 ppm more towards low field compared to the signals of 4m–t possessing an aryl substitution at N-5. The shielding effect of the aryl group at N-5 of 4m–t indicates the positioning of the phenyl out-of-plane of the pyrazolo[3,4-*d*]pyrimidin-4-one skeleton thus resulting in the anisotropic effect of the aryl substituent on H-6 located under the plane of this ring. Such an orientation of the phenyl ring at N-5 was further confirmed by X-ray crystallography and can be explained by the steric hindrance between the phenyl ring and oxygen atom of the carbonyl group.

### 2.2. X-ray crystallography

**Fig. 2:**
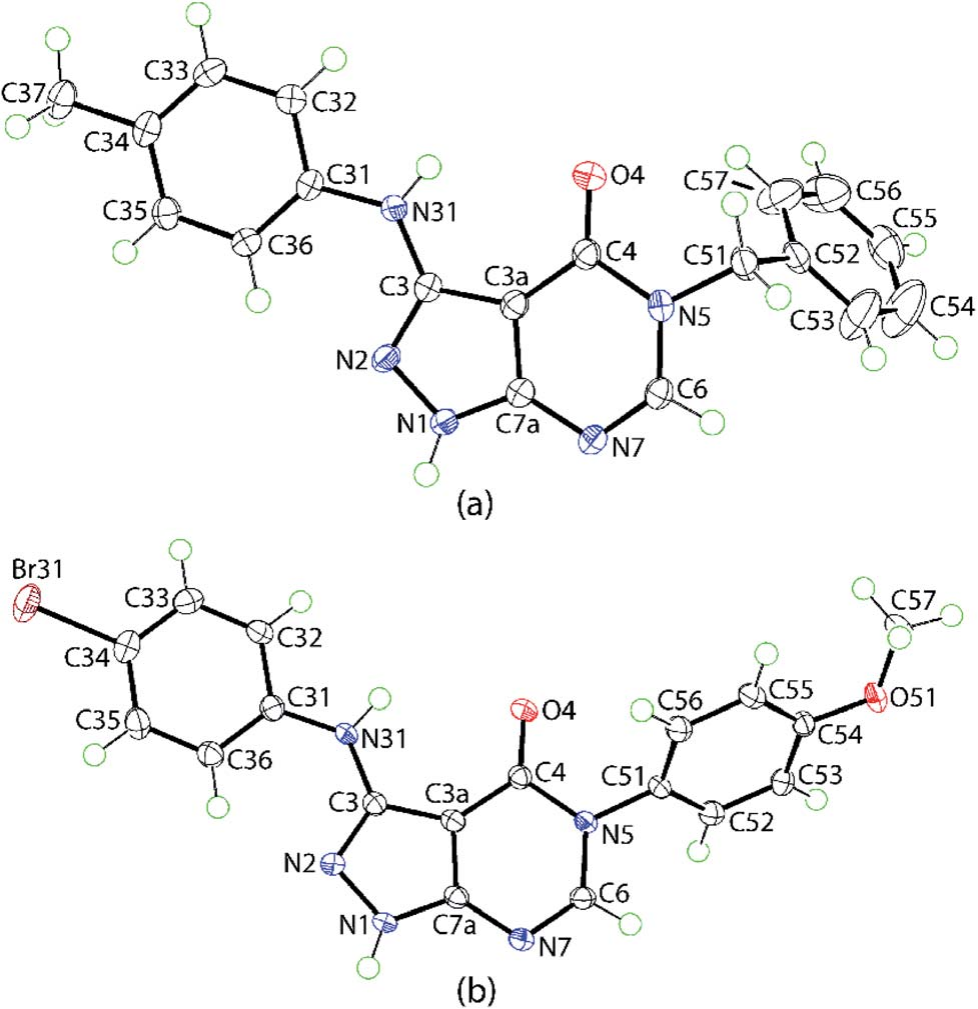
Molecular structures of (a) 4d and (b) 4p showing atom-labelling scheme and 70% anisotropic displacement ellipsoids.

The molecular structures of two representative derivatives were established by X-ray crystallography, thereby providing further supporting evidence for the structures of the products. The molecular structures of 4d and 4p are illustrated in Fig. 2 and selected geometric parameters are collated in Table 2. For 4d, the 10 atoms comprising the pyrazolo[3,4-*d*]pyrimidin-4-one core exhibit a root-mean-square (r.m.s.) deviation of 0.0124 Å with the maximum deviation from the least-squares plane being 0.0223(10) Å for the C3a atom. The dihedral angles between the central plane and the planes through the N-bound tolyl and phenyl rings are 4.36(5) and 71.10(4)°, respectively, indicating near to co-planar and perpendicular relationships; the dihedral angle between the outer rings is 69.35(4)°. The planarity of the fused-ring system coupled with the systematic variations in bond lengths, Table 2, suggest considerable delocalization of π-electron density in this residue. The most notable elongations are seen in the C3–N2 and C6–N7 bond lengths with commensurate shortening in the N1–N2, C3–C3a and C3a–C4 bonds.

**Table 2:** Selected geometric (Å) parameters for 4d and 4p

Parameter	4d	4p
N1–N2	1.3838(13)	1.3838(13)
C4–O4	1.2277(14)	1.2226(18)
C7a–N1	1.3337(14)	1.338(2)
C3–N2	1.3284(14)	1.3309(19)
C4–N5	1.4238(14)	1.431(2)
C6–N5	1.3660(14)	1.3718(19)
C6–N7	1.3006(14)	1.304(2)
C7a–N7	1.3734(14)	1.370(2)
C3–C3a	1.4256(15)	1.420(2)
C3a–C4	1.4187(15)	1.426(2)
C3a–C7a	1.3877(15)	1.388(2)

The molecular structure of 4p shows features similar to those exhibited by 4d. The 10-membered core exhibits minor distortions from planarity having a r.m.s. deviation of 0.0319 Å with the maximum deviation of 0.0605(13) Å noted for the C3a atom; the dihedral angle between the five- and six-membered rings = 4.52(8)°. The dihedral angles between the central plane and the planes through the N-bound bromo- and methoxy-phenyl rings are 13.15(6)° and 58.81(3)°, respectively, are indicative of significant twisting in the molecule; the dihedral angle between the outer rings is 52.82(4)°.

**Fig. 3:**
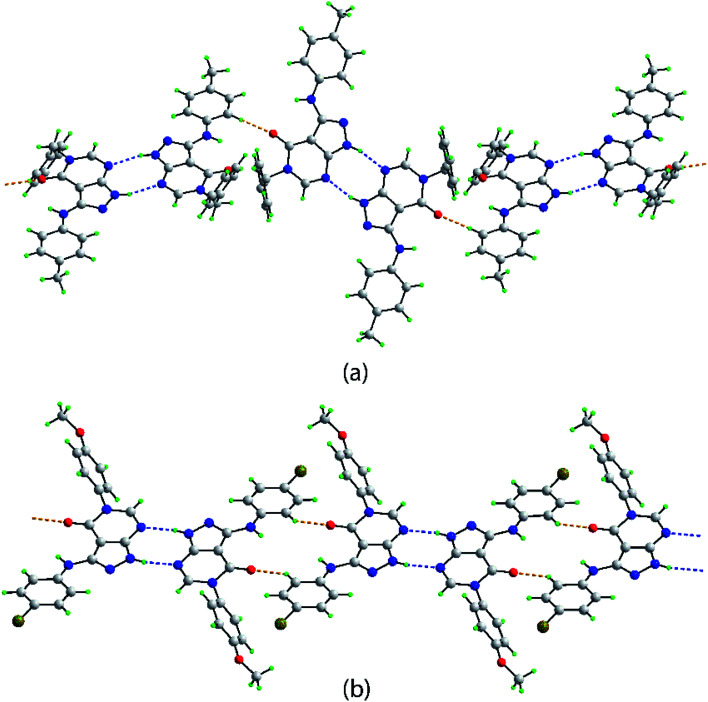
Supramolecular tape mediated by pyrazolyl-N–H⋯N(pyrimidyl) and C–H⋯O(carbonyl) interactions shown as blue and orange dashed lines, respectively in the crystals of (a) 4d and (b) 4p.

In both 4d and 4p, an intramolecular amine-N–H⋯O(carbonyl) hydrogen bond is noted (Table 3). Hydrogen bonding features prominently in the supramolecular association evident in the crystals of 4d and 4p, Table 3. Centrosymmetric dimers are formed in the crystal of 4d, being mediated by pyrazolyl-N–H⋯N(pyrimidyl) hydrogen bonds giving rise to eight-membered {⋯NCNH}_2_ synthons. These are connected into a supramolecular chain with a twisted topology *via* rather short tolyl-C–H⋯O(carbonyl) interactions, Fig. 3a. As detailed in ESI Fig. S1,† the chains are connected into a three-dimensional architecture *via* π(pyrazolyl)⋯π(tolyl), π(pyrimidyl)⋯π(tolyl), methyl-C–H⋯π(pyrazolyl) and phenyl-C–H⋯π(pyrimidyl) interactions.

**Table 3:** Geometric parameters (Å, °) characterising the key intermolecular contacts in the crystals of 4d and 4p

Interaction (A–H⋯B)	H⋯B	A⋯B	A–H⋯B	Symmetry operation
4d				
N31–H31n⋯O1	2.589(12)	3.2035(12)	128.0(11)	*x*, *y*, *z*
N1–H1n⋯N7	2.061(12)	2.9215(12)	164.4(12)	1 − *x*, 1 − *y*, −*z*
C32–H32⋯O1	2.33	3.2751(14)	178	½ − *x*, ½ + *y*, ½ − *z*

4p				
N31–H31n⋯O1	2.590(17)	3.1991(17)	127.1(15)	*x*, *y*, *z*
N1–H1n⋯N7	2.039(18)	2.9005(18)	170.3(19)	3 − *x*, 2 − *y*, 1 − *z*
C32–H32⋯O1	2.37	3.2264(18)	150	1 − *x*, 1 − *y*, 1 − *z*

Centrosymmetric eight-membered {⋯NCNH}_2_ synthons are formed in the crystal of 4p. In this case, the dimeric aggregates are linked by relatively short bromophenyl-C–H⋯O(carbonyl) interactions which also associate about a center of inversion to form a flat, supramolecular tape, Fig. 3b. Connections between tapes to form a two-dimensional array comprise bromophenyl-C–H⋯π(methoxyphenyl), methoxyphenyl-C–H⋯π(bromophenyl) and π(pyrazolyl)⋯N(pyrimidyl) interactions. The connections between layers are of the type Br31⋯O51, with the separation of 3.1620(11) Å being indicative of a halogen bond; see ESI Fig. S2† for details.

## 3. Conclusions

In conclusion, we developed an efficient three-component method for the synthesis of 3,5-disubstituted pyrazolo[3,4-*d*]pyrimidin-4-ones 4 from methyl 5-aminopyrazolyl-4-carboxylates 3, trimethyl orthoformate, and primary amines. The reaction does not require any catalyst and proceeded selectively with a range of substrates as demonstrated by twenty examples. The three-component one-pot approach resulted in pot- and step-economy, while microwave-assisted protocol shortens the reaction time. Isolation of prepared pyrazolo[3,4-*d*]pyrimidin-4-ones 4 by simple filtration further contributes to the practicality of our method.

## 4. Experimental

### 4.1. General

Microwave-assisted reactions were carried out using a Discover SP microwave synthesizer (CEM, USA) applying the closed vessel focused single-mode operational protocol and controlling reaction temperature with the equipped IR sensor. The control experiment was also carried out in using a Monowave 50 (Anton Paar, Austria) reactor. Melting points (uncorrected) were measured using a Stuart SMP40 automatic melting point apparatus. ^1^H and ^13^C NMR spectra were recorded on a Bruker Fourier NMR spectrometer (300 MHz) using DMSO-*d*_6_ as a solvent and TMS as an internal reference.

### 4.2. General method for the synthesis of 5-substituted 3-arylaminopyrazolo[3,4-*d*]pyrimidin-4-ones (4)

Substituted 5-aminopyrazole-4-carboxylates 3 (1 mmol), trimethyl orthoformate (0.33 mL, 3 mmol), and a primary amine (3 mmol) were added to EtOH (2 mL) in a 10 mL seamless pressure vial. The reaction mixture was irradiated in a Discover SP (CEM) microwave reactor operating at a maximal microwave power of 150 W and pressure limit of 435 psi at 160 °C for 55 min. After cooling, the precipitated product was isolated by vacuum filtration and recrystallised using an appropriate solvent.

#### 4.2.1 5-Benzyl-3-(phenylamino)-1,5-dihydro-4*H*-pyrazolo[3,4-*d*]pyrimidin-4-one (4a)

White solid; yield: 262 mg (83%); mp 238–240 °C (AcOEt).

^1^H NMR (300 MHz, DMSO-*d*_6_): *δ* 5.16 (3H, s, C*H*_2_Ph), 6.85 (1H, t, ^3^*J* = 7.3 Hz, H-4′), 7.24 (2H, t, ^3^*J* = 7.8 Hz, H-3′ and H-5′), 7.27–7.37 (5H, m, CH_2_*Ph*), 7.66 (2H, d, ^3^*J* = 7.7 Hz, H-2′ and H-6′), 7.91 (1H, s, NH), 8.51 (1H, s, H-6), 12.93 (1H, s, NH).

^13^C NMR (75 MHz, DMSO-*d*_6_): *δ* 47.9 (CH_2_), 93.3 (C-3a), 116.4 (2C), 119.8, 127.5 (3C), 128.5 (2C), 128.5 (2C), 137.1, 141.5, 147.3 (C-3), 151.2 (C-6), 151.9 (C-7a), 157.0 (C-4).

Anal. calcd for C_18_H_15_N_5_O: C, 68.13; H, 4.76; N, 22.07. Found: C, 67.95; H, 4.92; N, 21.83.

#### 4.2.2 5-Benzyl-3-(4-chlorophenylamino)-1,5-dihydro-4*H*-pyrazolo[3,4-*d*]pyrimidin-4-one (4b)

White solid; yield: 290 mg (82%); mp 278–280 °C (AcOEt).

^1^H NMR (300 MHz, DMSO-*d*_6_): *δ* 5.16 (2H, s, CH_2_), 7.24–7.37 (7H, m, H-3′, H-5′, H-2′′, H-3′′, H-4′′, H-5′′ and H-6′′), 7.72 (2H, d, ^3^*J* = 8.9 Hz, H-2′ and H-6′), 8.21 (1H, s, NH), 8.52 (1H, s, H-6), 13.00 (1H, s, NH).

^13^C NMR (75 MHz, DMSO-*d*_6_): *δ* 47.9 (CH_2_), 93.4 (C-3a), 118.0 (2C), 123.1, 127.5 (3C), 128.2 (2C), 128.5 (2C), 137.1, 140.6, 146.9 (C-3), 151.2 (C-6), 152.0 (C-7a), 156.8 (C-4).

Anal. calcd for C_18_H_14_ClN_5_O: C, 61.46; H, 4.01; N, 19.91. Found: C, 61.33; H, 4.16; N, 19.79.

#### 4.2.3 5-Benzyl-3-(4-bromophenylamino)-1,5-dihydro-4*H*-pyrazolo[3,4-*d*]pyrimidin-4-one (4c)

White solid; yield: 329 mg (83%); mp 283–285 °C (AcOEt).

^1^H NMR (300 MHz, DMSO-*d*_6_): *δ* 5.16 (2H, s, CH_2_), 7.24–7.37 (5H, m, H-2′′, H-3′′, H-4′′, H-5′′ and H-6′′), 7.39 (2H, d, ^3^*J* = 8.9 Hz, H-2′ and H-6′), 7.66 (2H, d, ^3^*J* = 9.0 Hz, H-3′ and H-5′), 8.22 (1H, s, NH), 8.52 (1H, s, H-6), 13.01 (1H, s, NH).

^13^C NMR (75 MHz, DMSO-*d*_6_): *δ* 47.9 (CH_2_), 93.4 (C-3a), 110.8, 118.5 (2C), 127.5 (3C), 128.5 (2C), 131.1 (2C), 137.1, 141.0, 146.8 (C-3), 151.2 (C-6), 152.0 (C-7a), 156.8 (C-4).

Anal. calcd for C_18_H_14_BrN_5_O: C, 54.56; H, 3.56; N, 17.67. Found: C, 54.48; H, 3.65; N, 17.54.

#### 4.2.4 5-Benzyl-3-(4-methylphenylamino)-1,5-dihydro-4*H*-pyrazolo[3,4-*d*]pyrimidin-4-one (4d)

White solid; yield: 198 mg (60%); mp 247–249 °C (AcOEt).

^1^H NMR (300 MHz, DMSO-*d*_6_): *δ* 2.23 (3H, s, CH_3_), 5.16 (2H, s, CH_2_), 7.05 (2H, d, ^3^*J* = 8.3 Hz, H-3′ and H-5′), 7.24–7.37 (5H, m, H-2′′, H-3′′, H-4′′, H-5′′ and H-6′′), 7.56 (2H, d, ^3^*J* = 8.3 Hz, H-2′ and H-6′), 7.79 (1H, s, NH), 8.50 (1H, s, H-6), 12.89 (1H, s, NH).

^13^C NMR (75 MHz, DMSO-*d*_6_): *δ* 20.2 (CH_3_), 47.8 (CH_2_), 93.1 (C-3a), 116.5 (2C), 127.5 (3C), 128.4, 128.5 (2C), 129.0 (2C), 137.1, 139.0, 147.5 (C-3), 151.2 (C-6), 151.9 (C-7a), 157.1 (C-4).

Anal. calcd for C_19_H_17_N_5_O: C, 68.87; H, 5.17; N, 21.13. Found: C, 68.02; H, 5.30; N, 20.99.

#### 4.2.5 5-Benzyl-3-(4-methoxyphenylamino)-1,5-dihydro-4*H*-pyrazolo[3,4-*d*]pyrimidin-4-one (4e)

White solid; yield: 243 mg (70%); mp 228–230 °C (MeOH).

^1^H NMR (300 MHz, DMSO-*d*_6_): *δ* 3.71 (3H, s, OCH_3_), 5.16 (2H, s, CH_2_), 6.85 (2H, d, ^3^*J* = 9.1 Hz, H-3′ and H-5′), 7.24–7.37 (5H, m, H-2′′, H-3′′, H-4′′, H-5′′ and H-6′′), 7.61 (2H, d, ^3^*J* = 9.0 Hz, H-2′ and H-6′), 7.73 (1H, s, NH), 8.49 (1H, s, H-6), 12.82 (1H, s, NH).

^13^C NMR (75 MHz, DMSO-*d*_6_): *δ* 47.8 (CH_2_), 55.1 (OCH_3_), 92.9 (C-3a), 113.8 (2C), 117.9 (2C), 127.5 (3C), 128.5 (2C), 135.0, 137.1, 147.8 (C-3), 151.1 (C-6), 151.9 (C-7a), 153.0, 157.0 (C-4).

Anal. calcd for C_19_H_17_N_5_O_2_: C, 65.69; H, 4.93; N, 20.16. Found: C, 65.56; H, 5.08; N, 19.00.

#### 4.2.6 3-(4-Bromophenylamino)-5-(4-fluorobenzyl)-1,5-dihydro-4*H*-pyrazolo[3,4-*d*]pyrimidin-4-one (4f)

White solid; yield: 316 mg (76%); mp 263–265 °C (AcOEt).

^1^H NMR (300 MHz, DMSO-*d*_6_): *δ* 5.15 (2H, s, CH_2_), 7.19 (2H, dd, ^3^*J*_HF_ = 8.8 Hz, ^3^*J*_HH_ = 8.8 Hz, H-3′′ and H-5′′), 7.35–7.48 (4H, m, H-2′, H-6′, H-2′′ and H-6′′), 7.68 (2H, d, ^3^*J* = 8.8 Hz, H-3′ and H-5′), 8.23 (1H, s, NH), 8.54 (1H, s, H-6), 13.03 (1H, s, NH).

^13^C NMR (75 MHz, DMSO-*d*_6_): *δ* 47.3 (CH_2_), 93.4 (C-3a), 110.8, 115.3 (d, ^2^*J*_CF_ = 21.4 Hz, C-3′′ and C-5′′), 118.5 (2C), 129.9 (d, ^3^*J*_CF_ = 8.4 Hz, C-2′′ and C-6′′), 131.1 (2C), 133.3 (d, ^4^*J*_CF_ = 3.0 Hz, C-1′′), 141.0, 146.8 (C-3), 151.2 (C-6), 152.0 (C-7a), 156.8 (C-4), 161.5 (d, ^1^*J*_CF_ = 243.6 Hz, C-4′′).

Anal. calcd for C_18_H_13_BrFN_5_O: C, 52.19; H, 3.16; N, 16.91. Found: C, 52.06; H, 3.32; N, 16.75.

#### 4.2.7 3-(4-Bromophenylamino)-5-(4-chlorobenzyl)-1,5-dihydro-4*H*-pyrazolo[3,4-*d*]pyrimidin-4-one (4g)

White solid; yield: 284 mg (66%); mp 273–275 °C (AcOEt).

^1^H NMR (300 MHz, DMSO-*d*_6_): *δ* 5.15 (2H, s, CH_2_), 7.35–7.44 (6H, m, H-2′, H-6′, H-2′′, H-3′′, H-5′′ and H-6′′), 7.67 (2H, d, ^3^*J* = 8.9 Hz, H-3′ and H-5′), 8.22 (1H, s, NH), 8.53 (1H, s, H-6), 13.03 (1H, s, NH).

^13^C NMR (75 MHz, DMSO-*d*_6_): *δ* 47.4 (CH_2_), 93.4 (C-3a), 110.8, 118.5 (2C), 128.4 (2C), 129.5 (2C), 131.1 (2C), 132.2, 136.1, 141.0, 146.8 (C-3), 151.2 (C-6), 152.0 (C-7a), 156.8 (C-4).

Anal. calcd for C_18_H_13_BrClN_5_O: C, 50.20; H, 3.04; N, 16.26. Found: C, 50.08; H, 3.16; N, 16.17.

#### 4.2.8 3-(4-Bromophenylamino)-5-(4-methylbenzyl)-1,5-dihydro-4*H*-pyrazolo[3,4-*d*]pyrimidin-4-one (4h)

White solid; yield: 321 mg (78%); mp 291–293 °C (AcOEt).

^1^H NMR (300 MHz, DMSO-*d*_6_): *δ* 2.27 (3H, s, CH_3_), 5.11 (2H, s, CH_2_), 7.15 (2H, d, ^3^*J* = 8.3 Hz, H-2′′ and H-6′′), 7.25 (2H, d, ^3^*J* = 8.0 Hz, H-3′′ and H-5′′), 7.39 (2H, d, ^3^*J* = 8.9 Hz, H-2′ and H-6′), 7.67 (2H, d, ^3^*J* = 8.9 Hz, H-3′ and H-5′), 8.21 (1H, s, NH), 8.50 (1H, s, H-6), 13.00 (1H, s, NH).

^13^C NMR (75 MHz, DMSO-*d*_6_): *δ* 20.6 (CH_3_), 47.6 (CH_2_), 93.4 (C-3a), 110.8, 118.5 (2C), 127.6 (2C), 129.0 (2C), 131.1 (2C), 134.1, 136.8, 141.0, 146.8 (C-3), 151.2 (C-6), 152.0 (C-7a), 156.8 (C-4).

Anal. calcd for C_19_H_16_BrN_5_O: C, 55.62; H, 3.93; N, 17.07. Found: C, 55.57; H, 4.05; N, 16.89.

#### 4.2.9 3-(4-Chlorophenylamino)-5-(4-picolyl)-1,5-dihydro-4*H*-pyrazolo[3,4-*d*]pyrimidin-4-one (4i)

Yellowish solid; yield: 247 mg (70%); mp 242–244 °C (MeOH).

^1^H NMR (300 MHz, DMSO-*d*_6_): *δ* 5.19 (2H, s, CH_2_), 7.24–7.30 (4H, m, H-3′, H-5′, H-2′′ and H-6′′), 7.72 (2H, d, ^3^*J* = 9.0 Hz, H-2′ and H-6′), 8.22 (1H, s, NH), 8.51 (1H, s, H-6), 8.53 (2H, d, ^3^*J* = 6.0 Hz, H-3′′ and H-5′′), 13.07 (1H, s, NH).

^13^C NMR (75 MHz, DMSO-*d*_6_): *δ* 47.3 (CH_2_), 93.4 (C-3a), 118.1 (2C), 122.0 (2C), 123.1, 128.2 (2C), 140.6, 145.9, 146.9 (C-3), 149.7 (2C), 151.3 (C-6), 152.1 (C-7a), 156.8 (C-4).

Anal. calcd for C_17_H_13_ClN_6_O: C, 57.88; H, 3.71; N, 23.82. Found: C, 57.64; H, 3.92; N, 23.60.

#### 4.2.10 3-(4-Bromophenylamino)-5-(4-picolyl)-1,5-dihydro-4*H*-pyrazolo[3,4-*d*]pyrimidin-4-one (4j)

White solid; yield: 294 mg (74%); mp 250–252 °C (MeOH).

^1^H NMR (300 MHz, DMSO-*d*_6_): *δ* 5.19 (2H, s, CH_2_), 7.28 (2H, d, ^3^*J* = 6.0 Hz, H-2′′ and H-6′′), 7.39 (2H, d, ^3^*J* = 8.9 Hz, H-2′ and H-6′), 7.66 (2H, d, ^3^*J* = 9.0 Hz, H-3′ and H-5′), 8.23 (1H, s, NH), 8.50 (1H, s, H-6), 8.53 (2H, d, ^3^*J* = 6.0 Hz, H-3′′ and H-5′′), 13.07 (1H, s, NH).

^13^C NMR (75 MHz, DMSO-*d*_6_): *δ* 47.3 (CH_2_), 93.4 (C-3a), 110.8, 118.5 (2C), 122.0 (2C), 131.1 (2C), 141.0, 145.9, 146.9 (C-3), 149.7 (2C), 151.3 (C-6), 152.1 (C-7a), 156.7 (C-4).

Anal. calcd for C_17_H_13_BrN_6_O: C, 51.40; H, 3.30; N, 21.16. Found: C, 51.31; H, 3.38; N, 21.03.

#### 4.2.11 3-(4-Methylphenylamino)-5-(4-picolyl)-1,5-dihydro-4*H*-pyrazolo[3,4-*d*]pyrimidin-4-one (4k)

White solid; yield: 259 mg (78%); mp 251–253 °C (MeOH).

^1^H NMR (300 MHz, DMSO-*d*_6_): *δ* 2.23 (3H, s, CH_3_), 5.19 (2H, s, CH_2_), 7.05 (2H, d, ^3^*J* = 8.3 Hz, H-3′ and H-5′), 7.28 (2H, d, ^3^*J* = 6.1 Hz, H-2′′ and H-6′′), 7.55 (2H, d, ^3^*J* = 8.3 Hz, H-2′ and H-6′), 7.80 (1H, s, NH), 8.48 (1H, s, H-6), 8.53 (2H, d, ^3^*J* = 6.0 Hz, H-3′′ and H-5′′), 12.93 (1H, s, NH).

^13^C NMR (75 MHz, DMSO-*d*_6_): *δ* 20.2 (CH_3_), 47.2 (CH_2_), 93.0 (C-3a), 116.5 (2C), 122.0 (2C), 128.5, 128.9 (2C), 139.0, 146.0, 147.5 (C-3), 149.7 (2C), 151.2 (C-6), 152.0 (C-7a), 157.0 (C-4).

Anal. calcd for C_18_H_16_N_6_O: C, 65.05; H, 4.85; N, 25.29. Found: C, 64.92; H, 5.01; N, 25.04.

#### 4.2.12 3-(4-Bromophenylamino)-5-(4-phenethyl)-1,5-dihydro-4*H*-pyrazolo[3,4-*d*]pyrimidin-4-one (4l)

White solid; yield: 311 mg (76%); mp 250–252 °C (AcOEt).

^1^H NMR (300 MHz, DMSO-*d*_6_): *δ* 2.99 (2H, t, ^3^*J* = 7.3 Hz, CH_2_), 4.17 (2H, t, ^3^*J* = 7.3 Hz, CH_2_), 7.19–7.35 (5H, m, H-2′′, H-3′′, H-4′′, H-5′′ and H-6′′), 7.41 (2H, d, ^3^*J* = 8.9 Hz, H-2′ and H-6′), 7.69 (2H, d, ^3^*J* = 8.9 Hz, H-3′ and H-5′), 8.06 (1H, s, NH), 8.25 (1H, s, H-6), 12.92 (1H, s, NH).

^13^C NMR (75 MHz, DMSO-*d*_6_): *δ* 34.7 (CH_2_), 46.4 (CH_2_), 93.4 (C-3a), 110.8, 118.5 (2C), 126.5 (2C), 128.5 (2C), 128.7, 131.1 (2C), 137.8, 141.1, 146.8 (C-3), 150.9 (C-6), 152.1 (C-7a), 156.9 (C-4).

Anal. calcd for C_19_H_16_BrN_5_O: C, 55.62; H, 3.93; N, 17.07. Found: C, 55.50; H, 4.06; N, 16.98.

#### 4.2.13 3-(4-Bromophenylamino)-5-(4-methylphenyl)-1,5-dihydro-4*H*-pyrazolo[3,4-*d*]pyrimidin-4-one (4m)

Light brown solid; yield: 85 mg (21%); mp 322–324 °C (MeCN).

^1^H NMR (300 MHz, DMSO-*d*_6_): *δ* 2.39 (3H, s, CH_3_), 7.34–7.42 (6H, m, H-2′, H-6′, H-2′′, H-3′′, H-5′′ and H-6′′), 7.66 (2H, d, ^3^*J* = 9.3 Hz, H-3′ and H-5′), 8.22 (1H, s, NH), 8.24 (1H, s, H-6), 13.10 (1H, s, NH).

^13^C NMR (75 MHz, DMSO-*d*_6_): *δ* 20.6 (CH_3_), 93.3 (C-3a), 110.9, 118.5 (2C), 127.4 (2C), 129.5 (2C), 131.1 (2C), 134.4, 138.1, 141.0, 147.2 (C-3), 150.7 (C-6), 151.8 (C-7a), 156.8 (C-4).

Anal. calcd for C_18_H_14_BrN_5_O: C, 54.56; H, 3.56; N, 17.67. Found: C, 54.38; H, 3.70; N, 17.38.

#### 4.2.14 5-(4-Methoxylphenyl)-3-phenylamino-1,5-dihydro-4*H*-pyrazolo[3,4-*d*]pyrimidin-4-one (4n)

Brown solid; yield: 103 mg (31%); mp 270–272 °C (MeCN).

^1^H NMR (300 MHz, DMSO-*d*_6_): *δ* 3.82 (3H, s, OCH_3_), 6.87 (1H, t, ^3^*J* = 7.3 Hz, H-4′), 7.08 (2H, d, ^3^*J* = 8.9 Hz, H-3′′ and H-5′′), 7.26 (2H, d, ^3^*J* = 7.9 Hz, H-3′ and H-5′), 7.41 (2H, d, ^3^*J* = 8.9 Hz, H-2′′ and H-6′′), 7.66 (2H, d, ^3^*J* = 8.0 Hz, H-2′ and H-6′), 7.91 (1H, s, NH), 8.23 (1H, s, H-6), 13.02 (1H, s, NH).

^13^C NMR (75 MHz, DMSO-*d*_6_): *δ* 55.4 (OCH_3_), 93.1 (C-3a), 114.1 (2C), 116.4 (2C), 119.9, 128.6 (2C), 128.8 (2C), 129.6, 141.4, 147.7 (C-3), 150.8 (C-6), 151.8 (C-7a), 157.2 (C-4), 159.1.

Anal. calcd for C_18_H_15_N_5_O_2_: C, 64.86; H, 4.54; N, 21.01. Found: C, 64.77; H, 4.64; N, 20.78.

#### 4.2.15 3-(4-Chlorophenylamino)-5-(4-methoxylphenyl)-1,5-dihydro-4*H*-pyrazolo[3,4-*d*]pyrimidin-4-one (4o)

Brown solid; yield: 143 mg (39%); mp 281–283 °C (MeOH).

^1^H NMR (300 MHz, DMSO-*d*_6_): *δ* 3.82 (3H, s, OCH_3_), 7.08 (2H, d, ^3^*J* = 8.9 Hz, H-3′′ and H-5′′), 7.28 (2H, d, ^3^*J* = 8.9 Hz, H-3′ and H-5′), 7.40 (2H, d, ^3^*J* = 8.9 Hz, H-2′′ and H-6′′), 7.71 (2H, d, ^3^*J* = 8.9 Hz, H-2′ and H-6′), 8.20 (1H, s, NH), 8.23 (1H, s, H-6), 13.07 (1H, s, NH).

^13^C NMR (75 MHz, DMSO-*d*_6_): *δ* 55.4 (OCH_3_), 93.3 (C-3a), 114.1 (2C), 118.0 (2C), 123.1, 128.3 (2C), 128.8 (2C), 129.6, 140.6, 147.2 (C-3), 150.9 (C-6), 151.9 (C-7a), 157.0 (C-4), 159.1.

Anal. calcd for C_18_H_14_ClN_5_O_2_: C, 58.78; H, 3.84; N, 19.04. Found: C, 58.72; H,3.92; N, 18.91.

#### 4.2.16 3-(4-Bromophenylamino)-5-(4-methoxylphenyl)-1,5-dihydro-4*H*-pyrazolo[3,4-*d*]pyrimidin-4-one (4p)

Light brown solid; yield: 220 mg (53%); mp 297–299 °C (MeOH).

^1^H NMR (300 MHz, DMSO-*d*_6_): *δ* 3.82 (3H, s, OCH_3_), 7.08 (2H, d, ^3^*J* = 8.9 Hz, H-3′′ and H-5′′), 7.37–7.42 (4H, m, H-2′, H-6′, H-2′′ and H-6′′), 7.66 (2H, d, ^3^*J* = 8.9 Hz, H-3′ and H-5′), 8.21 (1H, s, NH), 8.23 (1H, s, H-6), 13.08 (1H, s, NH).

^13^C NMR (75 MHz, DMSO-*d*_6_): *δ* 55.4 (OCH_3_), 93.3 (C-3a), 110.8, 114.1 (2C), 118.5 (2C), 128.8 (2C), 129.6, 131.1 (2C), 141.0, 147.2 (C-3), 150.9 (C-6), 151.9 (C-7a), 156.9 (C-4), 159.1.

Anal. calcd for C_18_H_14_BrN_5_O_2_: C, 51.15; H, 3.28; N, 17.54. Found: C, 50.96; H, 3.50; N, 17.34.

#### 4.2.17 5-(4-Methoxylphenyl)-3-(3-methylphenylamino)-1,5-dihydro-4*H*-pyrazolo[3,4-*d*]pyrimidin-4-one (4q)

Beige solid; yield: 109 mg (31%); mp 272–274 °C (MeOH).

^1^H NMR (300 MHz, DMSO-*d*_6_): *δ* 2.28 (3H, s, CH_3_), 3.82 (3H, s, OCH_3_), 6.69 (1H, d, ^3^*J* = 7.4 Hz, H-4′), 7.05–7.20 (3H, m, H-5′, H-3′′ and H-5′′), 7.38–7.55 (4H, m, H-2′, H-6′, H-2′′ and H-6′′), 7.78 (1H, s, NH), 8.22 (1H, s, H-6), 13.00 (1H, s, NH).

^13^C NMR (75 MHz, DMSO-*d*_6_): *δ* 21.3 (CH_3_), 55.4 (OCH_3_), 93.1 (C-3a), 113.7, 114.1 (2C), 116.9, 120.7, 128.5, 128.8 (2C), 129.6, 137.7, 141.3, 147.7 (C-3), 150.8 (C-6), 151.7 (C-7a), 157.2 (C-4), 159.1.

Anal. calcd for C_19_H_17_N_5_O_2_: C, 65.69; H, 4.93; N, 20.16. Found: C, 65.58; H, 5.04; N, 19.93.

#### 4.2.18 5-(4-Methoxylphenyl)-3-(4-methylphenylamino)-1,5-dihydro-4*H*-pyrazolo[3,4-*d*]pyrimidin-4-one (4r)

Brown solid; yield: 90 mg (26%); mp 255–257 °C (MeOH).

^1^H NMR (300 MHz, DMSO-*d*_6_): *δ* 2.24 (3H, s, CH_3_), 3.82 (3H, s, OCH_3_), 7.04–7.10 (4H, m, H-3′, H-5′, H-3′′ and H-5′′), 7.40 (2H, d, ^3^*J* = 8.9 Hz, H-2′ and H-6′), 7.55 (2H, d, ^3^*J* = 8.4 Hz, H-2′′ and H-6′′), 7.76 (1H, s, NH), 8.22 (1H, s, H-6), 12.96 (1H, s, NH).

^13^C NMR (75 MHz, DMSO-*d*_6_): *δ* 20.2 (CH_3_), 55.4 (OCH_3_), 92.9 (C-3a), 114.1 (2C), 116.5 (2C), 128.5, 128.8 (2C), 129.0 (2C), 129.6, 139.0, 147.8 (C-3), 150.8 (C-6), 151.7 (C-7a), 157.2 (C-4), 159.1.

Anal. calcd for C_19_H_17_N_5_O_2_: C, 65.69; H, 4.93; N, 20.16. Found: C, 65.57; H, 5.10; N, 19.96.

#### 4.2.19 3-(2-Methoxylphenylamino)-5-(4-methoxylphenyl)-1,5-dihydro-4*H*-pyrazolo[3,4-*d*]pyrimidin-4-one (4s)

Pink solid; yield: 101 mg (28%); mp 280–282 °C (EtOH).

^1^H NMR (300 MHz, DMSO-*d*_6_): *δ* 3.83 (3H, s, OCH_3_), 3.87 (3H, s, OCH_3_), 6.87 (1H, ddd, ^4^*J* = 1.6 Hz, ^3^*J* = 7.6 Hz, ^3^*J* = 7.6 Hz, H-4′), 6.96 (1H, ddd, ^4^*J* = 1.4 Hz, ^3^*J* = 7.7 Hz, ^3^*J* = 7.7 Hz, H-5′), 7.01 (1H, dd, ^4^*J* = 1.4 Hz, ^3^*J* = 7.9 Hz, H-3′), 7.09 (2H, d, ^3^*J* = 9.1 Hz, H-3′′ and H-5′′), 7.42 (2H, d, ^3^*J* = 8.9 Hz, H-2′′ and H-6′′), 7.91 (1H, s, H-6), 8.22–8.30 (2H, m, H-6′ and NH), 13.04 (1H, s, NH).

^13^C NMR (75 MHz, DMSO-*d*_6_): *δ* 55.4 (OCH_3_), 55.7 (OCH_3_), 93.0 (C-3a), 110.0, 114.2 (2C), 115.2, 119.9, 120.7, 128.9 (2C), 129.4, 130.1, 146.1, 147.6 (C-3), 151.0 (C-6), 151.6 (C-7a), 157.6 (C-4), 159.2.

Anal. calcd for C_19_H_17_N_5_O_3_: C, 62.80; H, 4.72; N, 19.27. Found: C, 62.73; H, 4.87; N, 19.06.

#### 4.2.20 3-(4-Methoxylphenylamino)-5-(4-methoxylphenyl)-1,5-dihydro-4*H*-pyrazolo[3,4-*d*]pyrimidin-4-one (4t)

Brown solid; yield: 99 mg (27%); mp 277–279 °C (MeOH).

^1^H NMR (300 MHz, DMSO-*d*_6_): *δ* 3.71 (3H, s, OCH_3_), 3.82 (3H, s, OCH_3_), 6.86 (2H, d, ^3^*J* = 9.0 Hz, H-3′ and H-5′), 7.08 (2H, d, ^3^*J* = 8.9 Hz, H-3′′ and H-5′′), 7.40 (2H, d, ^3^*J* = 8.9 Hz, H-2′′ and H-6′′), 7.60 (2H, d, ^3^*J* = 9.0 Hz, H-2′ and H-6′), 7.70 (1H, s, NH), 8.21 (1H, s, H-6), 12.89 (1H, s, NH).

^13^C NMR (75 MHz, DMSO-*d*_6_): *δ* 55.1 (OCH_3_), 55.4 (OCH_3_), 92.7 (C-3a), 113.9 (2C), 114.1 (2C), 117.9 (2C), 128.8 (2C), 129.6, 135.0, 148.1 (C-3), 150.8 (C-6), 151.7 (C-7a), 153.1, 157.2 (C-4), 159.1.

Anal. calcd for C_19_H_17_N_5_O_3_: C, 62.80; H, 4.72; N, 19.27. Found: C, 62.69; H, 4.80; N, 19.15.

### 4.3. X-ray crystallographic analysis

X-ray intensity data for a colourless crystal of 4d and a yellow crystal of 4p were measured at *T* = 100 K on Rigaku/Oxford Diffraction XtaLAB Synergy diffractometer (Dualflex, AtlasS2) fitted with CuKα radiation (*λ* = 1.54178 Å) so that *θ*_max_ = 67.7° (corresponding to 100% completeness). Data reduction and Gaussian absorption correction were accomplished with CrysAlisPro.^33^ The structures were solved by direct-methods^34^ and refined (anisotropic displacement parameters and C-bound H atoms in the riding model approximation) on *F*^2^.^35^ The N-bound H atoms were refined with the N–H bond lengths constrained to 0.88 ± 0.01 Å, and with *U*_iso_(H) = 1.2*U*_eq_(N). A weighting scheme of the form *w* = 1/[*σ*^2^(*F*_o_^2^) + (*aP*)^2^ + *bP*] where *P* = (*F*_o_^2^ + 2*F*_c_^2^)/3 was introduced towards the end of the refinement in each case. The molecular structure diagrams were generated with ORTEP for Windows^36^ with 70% displacement ellipsoids, and the packing diagrams were drawn with DIAMOND.^37^ Additional data analysis was made with PLATON.^38^

#### 4.3.1 Crystal data for 4d^39^

C_19_H_17_N_5_O, *M* = 331.38, monoclinic, *P*2_1_/*n*, *a* = 11.5648(1), *b* = 6.5343(1), *c* = 21.5417(2) Å, *β* = 97.443(1)°, *V* = 1614.14(3) Å^3^, *Z* = 4, *D*_*x*_ = 1.364 g cm^−3^, *F*(000) = 696, *μ* = 0.716 mm^−1^, no. reflns meas. = 21 624, no. unique reflns = 3338 (*R*_int_ = 0.030), no. reflns with *I* ≥ 2.0*σ*(*I*) = 3105, no. parameters = 233, *R*(obs. data) = 0.039, *a* and *b* in weighting scheme = 0.051 and 0.737, w*R*_2_ (all data) = 0.101.

#### 4.3.2 Crystal data for 4p^39^

C_18_H_14_BrN_5_O_2_, *M* = 412.25, triclinic, *P*1̄, *a* = 6.3686(3), *b* = 7.5121(3), *c* = 17.5514(5) Å, *α* = 85.174(3), *β* = 80.226(3), *γ* = 88.510(3)°, *V* = 824.51(6) Å^3^, *Z* = 2, *D*_*x*_ = 1.661 g cm^−3^, *F*(000) = 416, *μ* = 3.614 mm^−1^, no. reflns meas. = 21 039, no. unique reflns = 3394 (*R*_int_ = 0.036), no. reflns with *I* ≥ 2.0*σ*(*I*) = 3299, no. parameters = 242, *R*(obs. data) = 0.026, *a* and *b* in weighting scheme = 0.030 and 0.675, w*R*_2_ (all data) = 0.064.

## Conflicts of interest

There are no conflicts to declare.

## Supplementary Material

RA-012-D2RA00980C-s001

RA-012-D2RA00980C-s002

RA-012-D2RA00980C-s003

RA-012-D2RA00980C-s004

## References

[cit1] Rashad A. E., Shamroukh A. H., Abdel-Megeid R. E., Mostafa A., Ali M. A., Banert K. (2010). Nucleosides, Nucleotides Nucleic Acids.

[cit2] Bondock S., Rabie R., Etman H. A., Fadda A. A. (2008). Eur. J. Med. Chem..

[cit3] Bondock S., Fadaly W., Metwally M. A. (2010). Eur. J. Med. Chem..

[cit4] Shamroukh A. H., Rashad A. E., Ali H. S., Abdel-Megeid F. M. E. (2013). J. Heterocycl. Chem..

[cit5] Fekri A., Keshk E. M., Khalil A.-G. M., Taha I. (2022). Mol. Diversity.

[cit6] Allam M., Bhavani A. K. D., Mudiraj A., Ranjan N., Thippana M., Babu P. P. (2018). Eur. J. Med. Chem..

[cit7] Kosbar T. R., Abou-Zeid L., Sofan M. A. (2018). J. Heterocycl. Chem..

[cit8] Li M., Liu S., Chen H., Zhou X., Zhou J., Zhou S., Yuan H., Xu Q.-L., Liu J., Cheng K., Sun H., Wang Y., Chen C., Wen X. (2020). Eur. J. Med. Chem..

[cit9] Turnbull A. P., Ioannidis S., Krajewski W. W., Pinto-Fernandez A., Heride C., Martin A. C. L., Tonkin L. M., Townsend E. C., Buker S. M., Lancia D. R., Caravella J. A., Toms A. V., Charlton T. M., Lahdenranta J., Wilker E., Follows B. C., Evans N. J., Stead L., Alli C., Zarayskiy V. V., Talbot A. C., Buckmelter A. J., Wang M., McKinnon C. L., Saab F., McGouran J. F., Century H., Gersch M., Pittman M. S., Marshall C. G., Raynham T. M., Simcox M., Stewart L. M. D., McLoughlin S. B., Escobedo J. A., Bair K. W., Dinsmore C. J., Hammonds T. R., Kim S., Urbé S., Clague M. J., Kessler B. M., Komander D. (2017). Nature.

[cit10] Subramanian G., Vairagoundar R., Bowen S. J., Roush N., Zachary T., Javens C., Williams T., Janssen A., Gonzales A. (2020). RSC Med. Chem..

[cit11] Francis S., Croft D., Schüttelkopf A. W., Parry C., Pugliese A., Cameron K., Claydon S., Drysdale M., Gardner C., Gohlke A., Goodwin G., Gray C. H., Konczal J., McDonald L., Mezna M., Pannifer A., Paul N. R., Machesky L., McKinnon H., Bower J. (2019). Bioorg. Med. Chem. Lett..

[cit12] Abdelazeem A. H., Abdelatef S. A., El-Saadi M. T., Omar H. A., Khan S. I., McCurdy C. R., El-Moghazy S. M. (2014). Eur. J. Pharm. Sci..

[cit13] Cheng C. C., Robins R. K. (1956). J. Org. Chem..

[cit14] Shamroukh A. H., Rashad A. E., Sayed H. H. (2005). Phosphorus, Sulfur Silicon Relat. Elem..

[cit15] Ram V. J., Pandey H. N., Mishra L. (1979). Arch. Pharm..

[cit16] Ram V. J., Pandey H. (1979). Arch. Pharm..

[cit17] Andreassend S. K., Bentley S. J., Blatch G. L., Boshoff A., Keyzers R. A. (2020). Mar. Drugs.

[cit18] Bulychev Y. N., Preobrazhenskaya M. N. (1988). Chem. Heterocycl. Compd..

[cit19] Heo H. G., Yu J., Jillella R., Oh C. H. (2018). Synth. Commun..

[cit20] Dawood K. M., Farag A. M., Khedr N. A. (2008). ARKIVOC.

[cit21] Hassan A. Y., Sarg M. T., El-Sebaey S. A. (2019). J. Heterocycl. Chem..

[cit22] Gouda M. A., Berghot M. A., Shoeib A. I., Khalil A. M. (2010). Eur. J. Med. Chem..

[cit23] Bondock S., Tarhoni A. E.-G., Fadda A. A. (2015). J. Heterocycl. Chem..

[cit24] Finlander P., Pedersen E. B. (1985). Arch. Pharm..

[cit25] Makarov V. A., Ryabova O. B., Alekseeva L. M., Shashkov A. S., Granik V. G. (2003). Chem. Heterocycl. Compd..

[cit26] Lim F. P. L., Tan K. C., Luna G., Tiekink E. R. T., Dolzhenko A. V. (2019). Tetrahedron.

[cit27] Lim F. P. L., Halcovitch N. R., Tiekink E. R. T., Dolzhenko A. V. (2018). Tetrahedron.

[cit28] Lim F. P. L., Luna G., Dolzhenko A. V. (2015). Tetrahedron Lett..

[cit29] Lim F. P. L., Luna G., Dolzhenko A. V. (2015). Tetrahedron Lett..

[cit30] Lim F. P. L., Luna G., Dolzhenko A. V. (2014). Tetrahedron Lett..

[cit31] Lim F. P. L., Luna G., Dolzhenko A. V. (2016). Synthesis.

[cit32] Lim F. P. L., Gan R. X. Y., Dolzhenko A. V. (2017). Tetrahedron Lett..

[cit33] Rigaku Oxford Diffraction , CrysAlis PRO, Yarnton, Oxfordshire, 2018

[cit34] Sheldrick G. M. (2008). Acta Crystallogr., Sect. A: Found. Crystallogr..

[cit35] Sheldrick G. M. (2015). Acta Crystallogr., Sect. C: Struct. Chem..

[cit36] Farrugia L. J. (2012). J. Appl. Crystallogr..

[cit37] Brandenburg K. , Diamond, Crystal Impact GbR, Bonn, Germany, 2006

[cit38] Spek A. L. (2019). Acta Crystallogr., Sect. E: Crystallogr. Commun..

[cit39] CCDC 2143130 (4d) and 2143131 (4p) contain the supplementary crystallographic data for this paper.†

