# Supplementary material for: Three-component microwave-assisted synthesis of 3,5-disubstituted pyrazolo[3,4-d]pyrimidin-4-ones
Source: RSC Adv. 2022 Mar 16;12(14):8323–32. doi: 10.1039/d2ra00980c (PMC8984946; doi:10.1039/d2ra00980c)

Electronic Supplementary Material (ESI) for RSC Advances.
This journal is © The Royal Society of Chemistry 2022

RSC Advances

Electronic Supplementary Information

Three-Component Microwave-Assisted Synthesis of 3,5-Disubstituted Pyrazolo[3,4-*d*]pyrimidin-4-ones

Jia Hui Ng,[a] *Edward R. T. Tiekink*[b] *and Anton V. Dolzhenko**[a,c]

[a] School of Pharmacy, Monash University Malaysia, Jalan Lagoon Selatan, Bandar Sunway, Selangor Darul Ehsan 47500, Malaysia

[b] Research Centre for Crystalline Materials, School of Medical and Life Sciences, Sunway University, 5 Jalan Universiti, Bandar Sunway, Selangor Darul Ehsan 47500, Malaysia

[c] Curtin Medical School, Curtin Health Innovation Research Institute, Faculty of Health Sciences, Curtin University, GPO Box U1987 Perth, Western Australia 6845, Australia

Contents

Molecular packing in 5-benzyl-3-(4-methylphenylamino)-1,5-dihydro-4*H*-pyrazolo[3,4-*d*]pyrimidin-4-one (**4d**) ……………………3

Molecular packing in 3-(4-bromophenylamino)-5-(4-methoxylphenyl)-1,5-dihydro-4*H*-pyrazolo[3,4-*d*]pyrimidin-4-one (**4p**) ……………………4

Copies of ^{1}H and ^{13}C NMR spectra for prepared 3,5-disubstituted pyrazolo[3,4-*d*]pyrimidin-4-ones (**4a-t**) …………………….5

Molecular packing in 5-benzyl-3-(4-methylphenylamino)-1,5-dihydro-4*H*-pyrazolo[3,4-*d*]pyrimidin-4-one (4d)

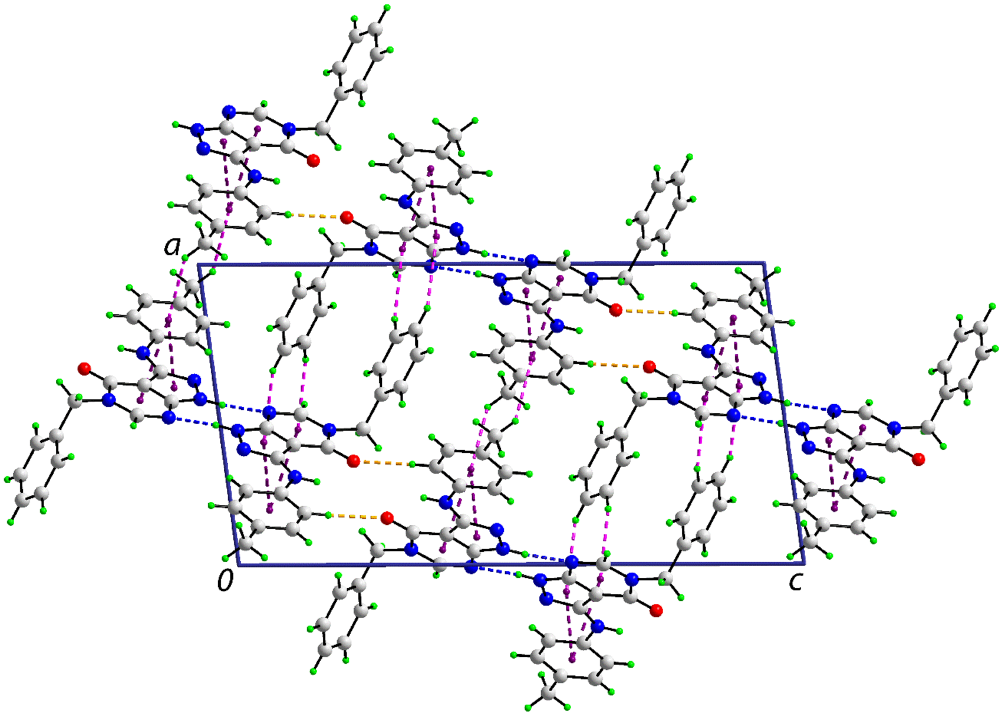

Figure S1. Molecular packing in **4d**: a view in projection down the *b*-axis of the unit cell contents. The N–H···N, C–H···O, C–H···π and π···π interactions are shown as blue, orange, pink and purple dashed lines, respectively.

Geometric parameters (Å, °) characterizing the identified intermolecular interactions

Interaction (A–H···B)	H···B	A···B	A–H···B	Symmetry operation
N31–H31···O1	2.589(12)	3.2035(12)	128.0(11)	*x*, *y*, *z*
N1–H1n···N7	2.061(12)	2.9215(12)	164.4(12)	1-*x*, 1-*y*, -*z*
C32–H32···O1	2.33	3.2751(14)	178	½-*x*, ½+*y*, ½-*z*
C37–H37b···Cg3	2.71	3.5213(13)	141	-*x*, 3-*y*, -*z*
C54–H54···Cg(N5,N7,C3a,C4,C6,C7a)				
	2.76	3.5913(18)	146	1½-*x*, -½+*y*, ½-*z*
C55–H55··· Cg(N1,N2,C3,C3a,C7a)				
	2.91	3.7465(15)	147	1½2-*x*, -½+*y*, ½-*z*
Cg(N1,N2,C3,C3a,C7a)···Cg(C31-C36)				
		3.5566(6)	3.46(6)	*x*, -1+*y*, *z*
Cg(N5,N7,C3a,C4,C6,C7a)···Cg(C31-C36)				
		3.3616(6)	5.03(5)	*x*, -1+*y*, *z*

Molecular packing in 3-(4-bromophenylamino)-5-(4-methoxylphenyl)-1,5-dihydro-4*H*-pyrazolo[3,4-*d*]pyrimidin-4-one (4p)

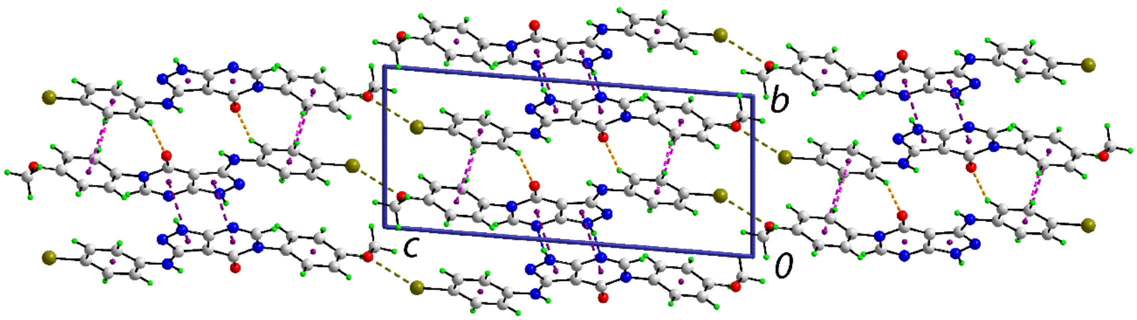

Figure S2. Molecular packing in **4p**: a view in projection down the *a*-axis of the unit-cell contents. The N–H···N, C–H···O, C–H···π, π···π and Br···O interactions are shown as blue, orange, pink, purple and olive dashed lines, respectively.

Geometric parameters (Å, °) characterizing the identified intermolecular interactions:

Interaction (A–H···B)	H···B	A···B	A–H···B	Symmetry operation
N1–H1n···N7	2.039(18)	2.9005(18)	170.3(19)	3-*x*, 2-*y*, 1-*z*
C32–H32···O1	2.37	3.2264(18)	150	1-*x*, 1-*y*, 1-*z*
C33–H33···Cg(C51-C56)				
	2.86	3.6487(16)	142	1-*x*, 1-*y*, 1-*z*
C52–H52···Cg(C31-C36)				
	2.51	3.2868(16)	139	2-*x*, 1-*y*, 1-*z*
Cg(N1,N2,C3,C3a,C7a)···Cg(N5,N7,C3a,C4,C6,C7a)				
		3.4144(8)	4.52(8)	2-*x*, 2-*y*, 1-*z*
C34–Br31···O51		3.1620(11)	154.75(6)	-1+*x*, *y*, 1+*z*

Copies of ^{1}H and ^{13}C NMR spectra for prepared

3,5-disubstituted pyrazolo[3,4-*d*]pyrimidin-4-ones (4a-t)

5-Benzyl-3-(phenylamino)-1,5-dihydro-4*H*-pyrazolo[3,4-*d*]pyrimidin-4-one (4a)

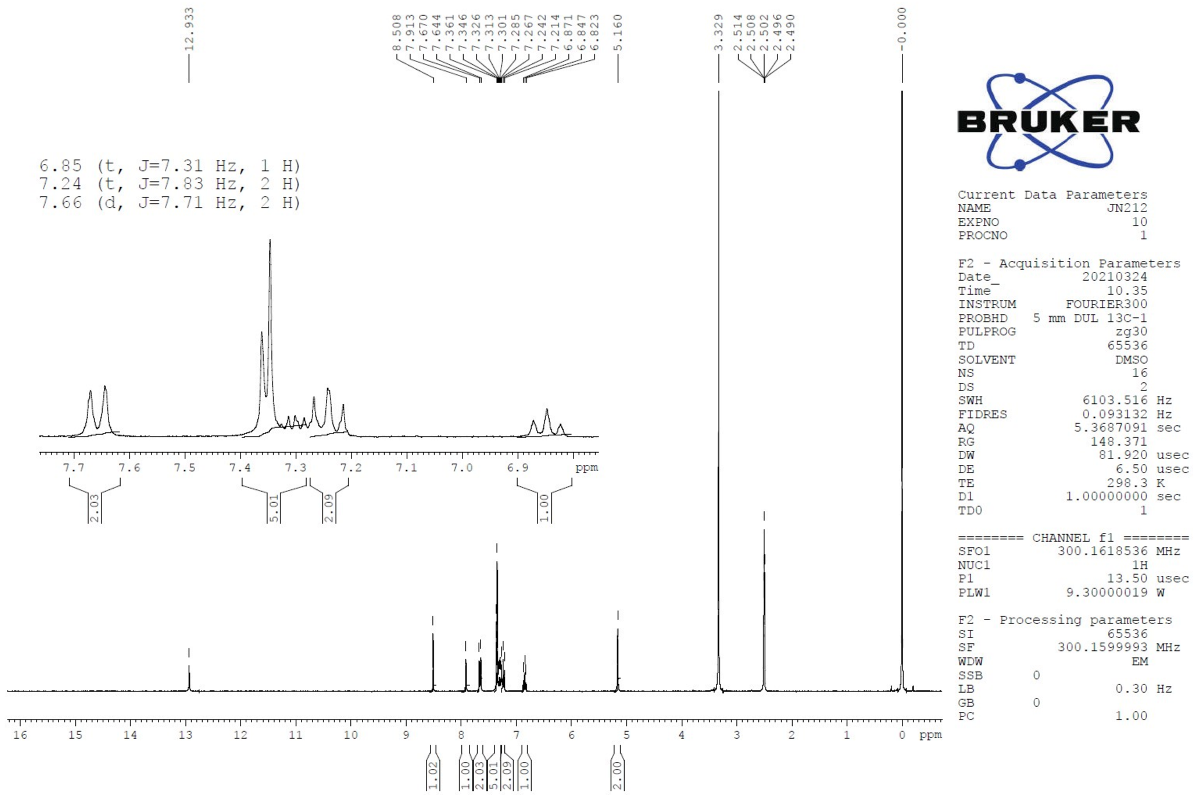

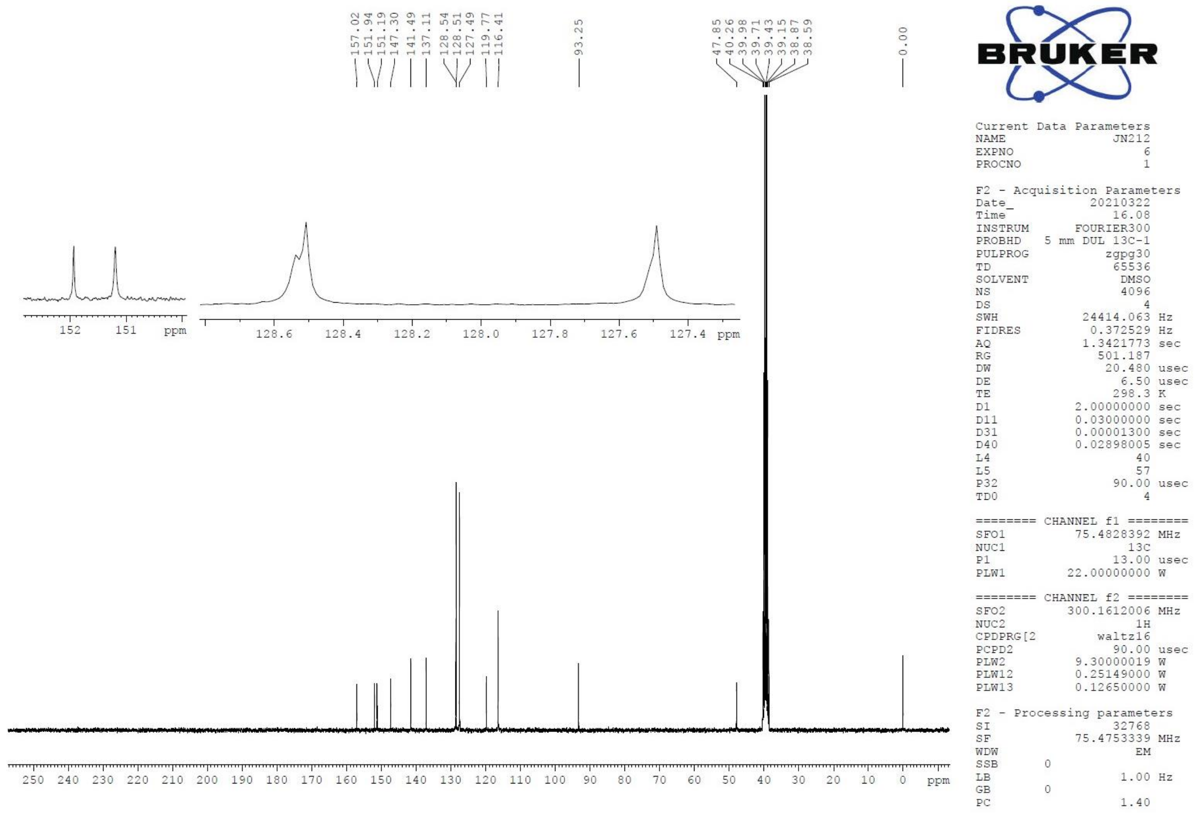

5-Benzyl-3-(phenylamino)-1,5-dihydro-4*H*-pyrazolo[3,4-*d*]pyrimidin-4-one (4a)

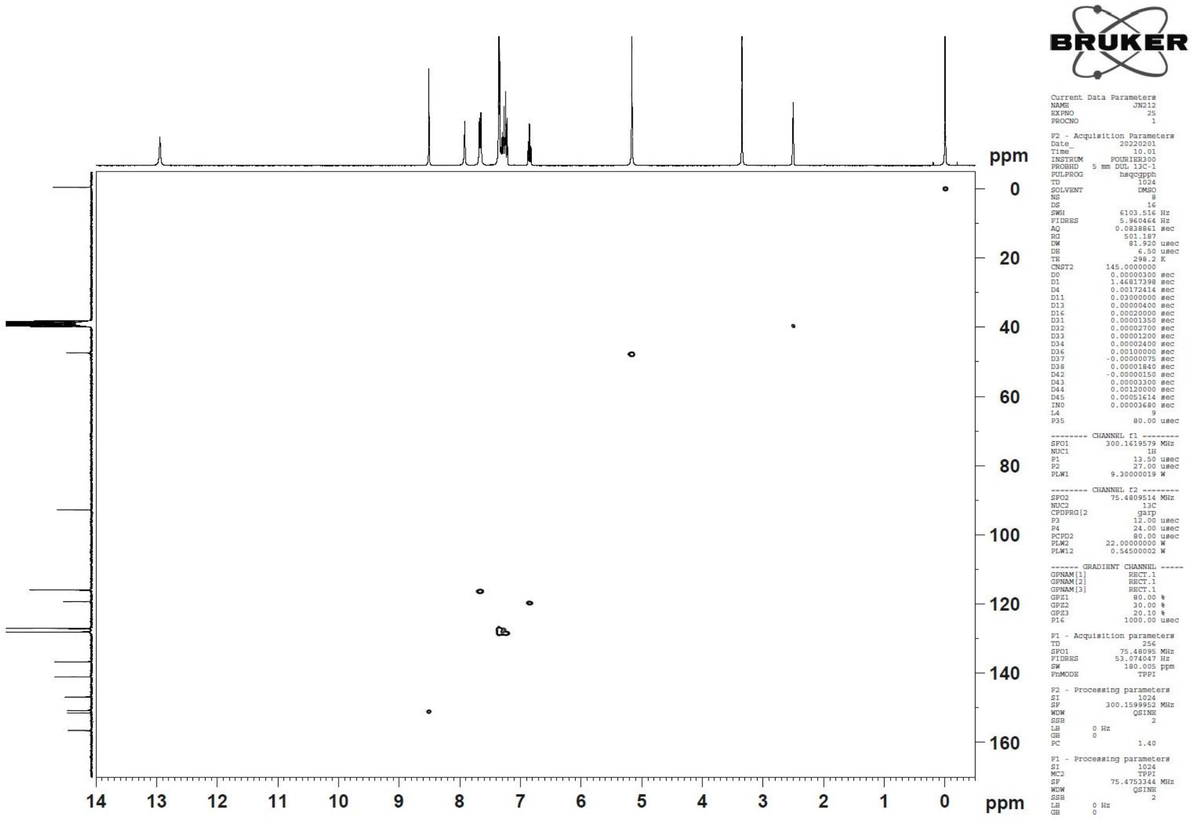

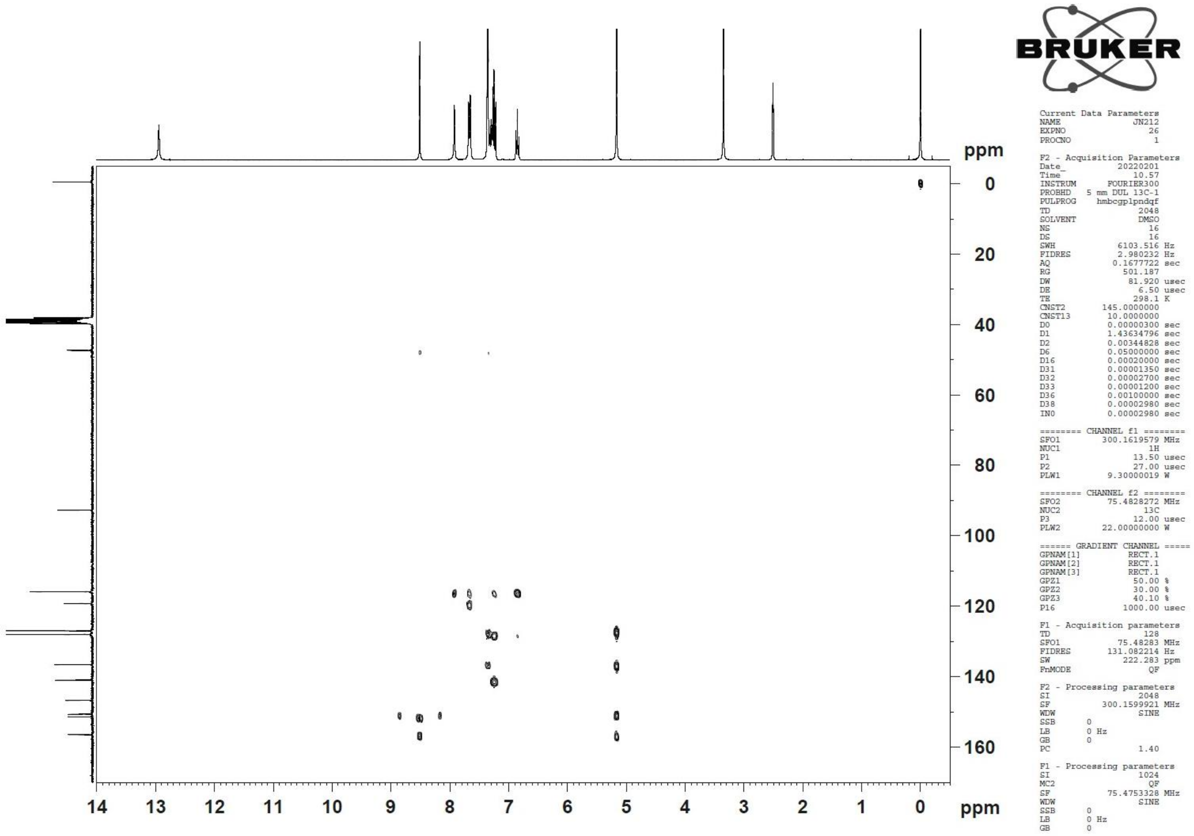

5-Benzyl-3-(4-chlorophenylamino)-1,5-dihydro-4*H*-pyrazolo[3,4-*d*]pyrimidin-4-one (4b)

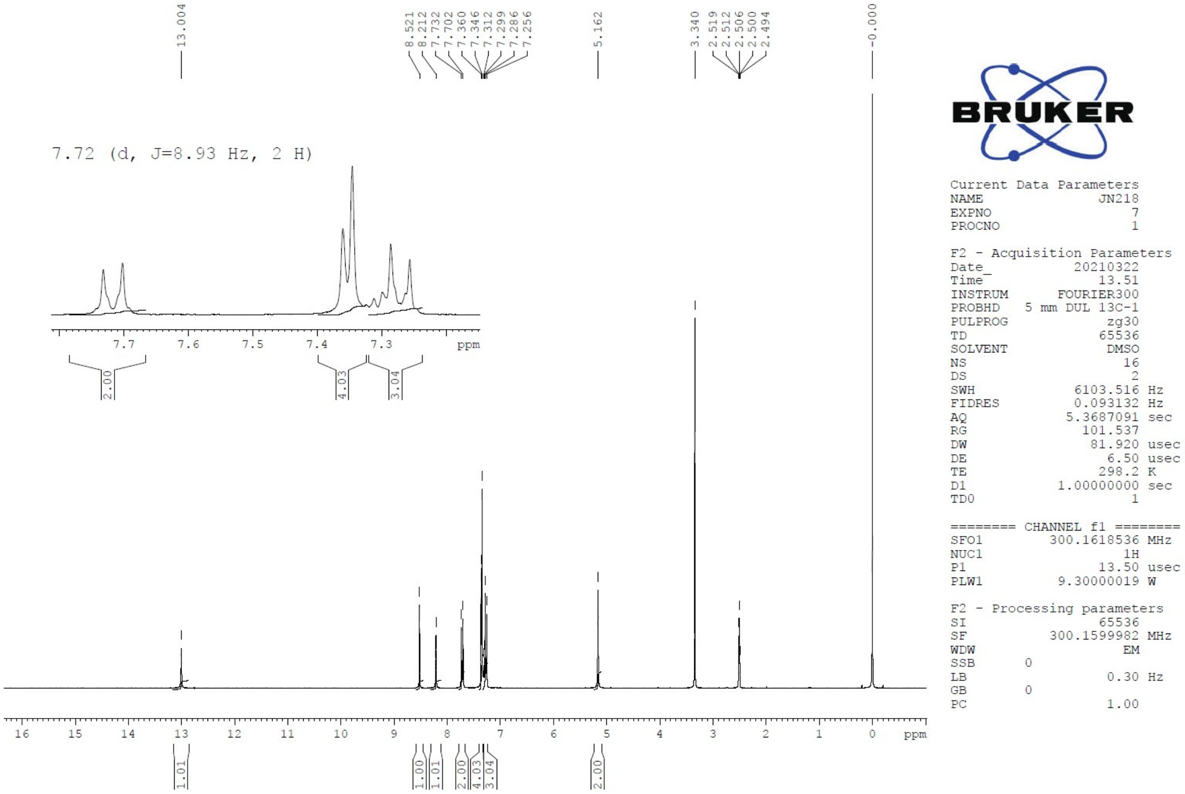

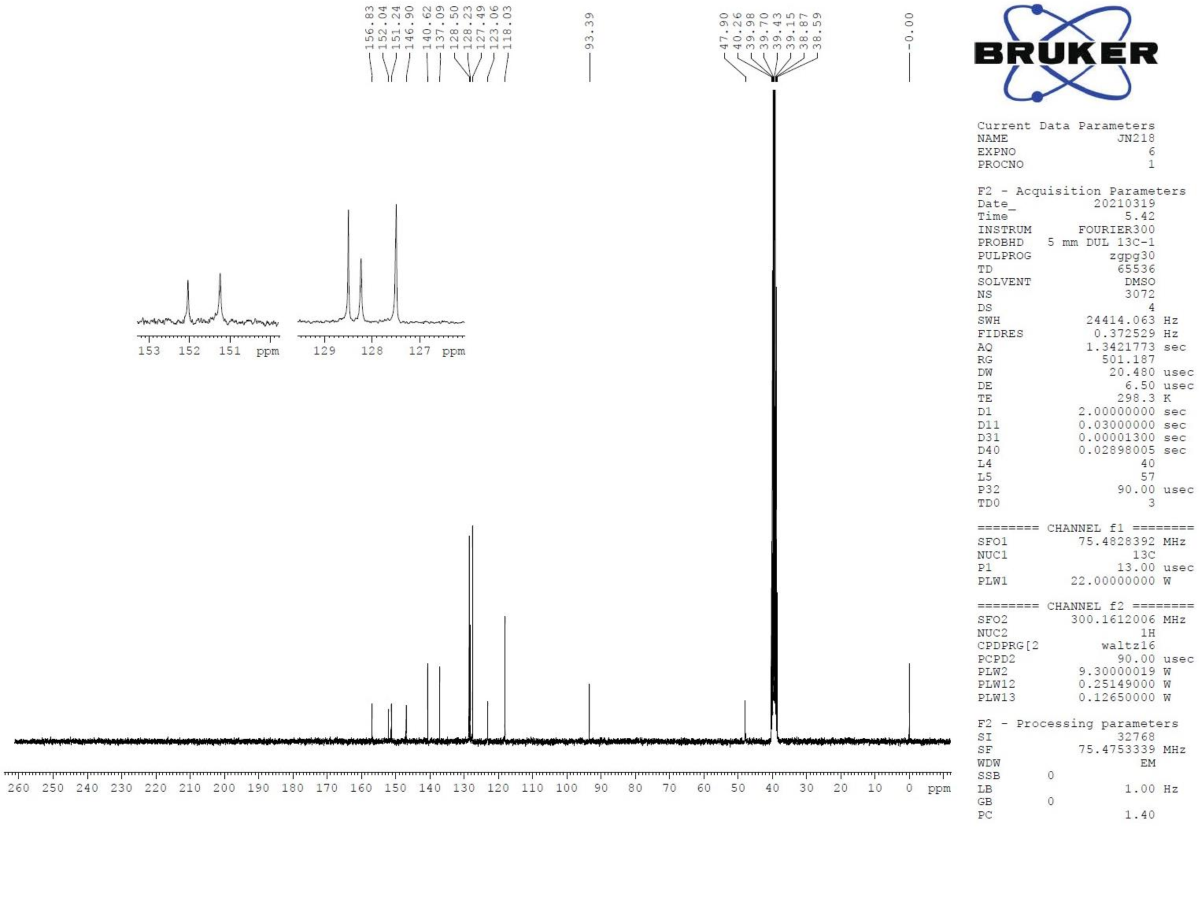

5-Benzyl-3-(4-bromophenylamino)-1,5-dihydro-4*H*-pyrazolo[3,4-*d*]pyrimidin-4-one (4c)

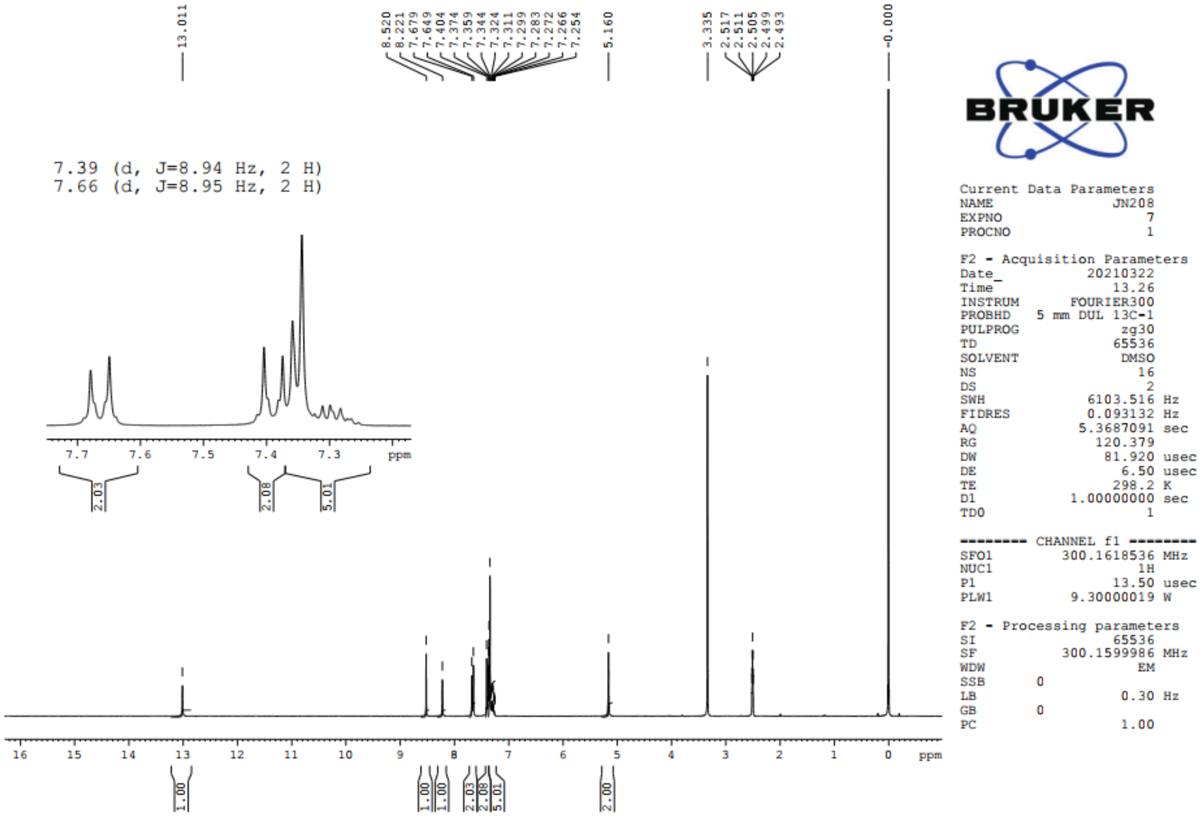

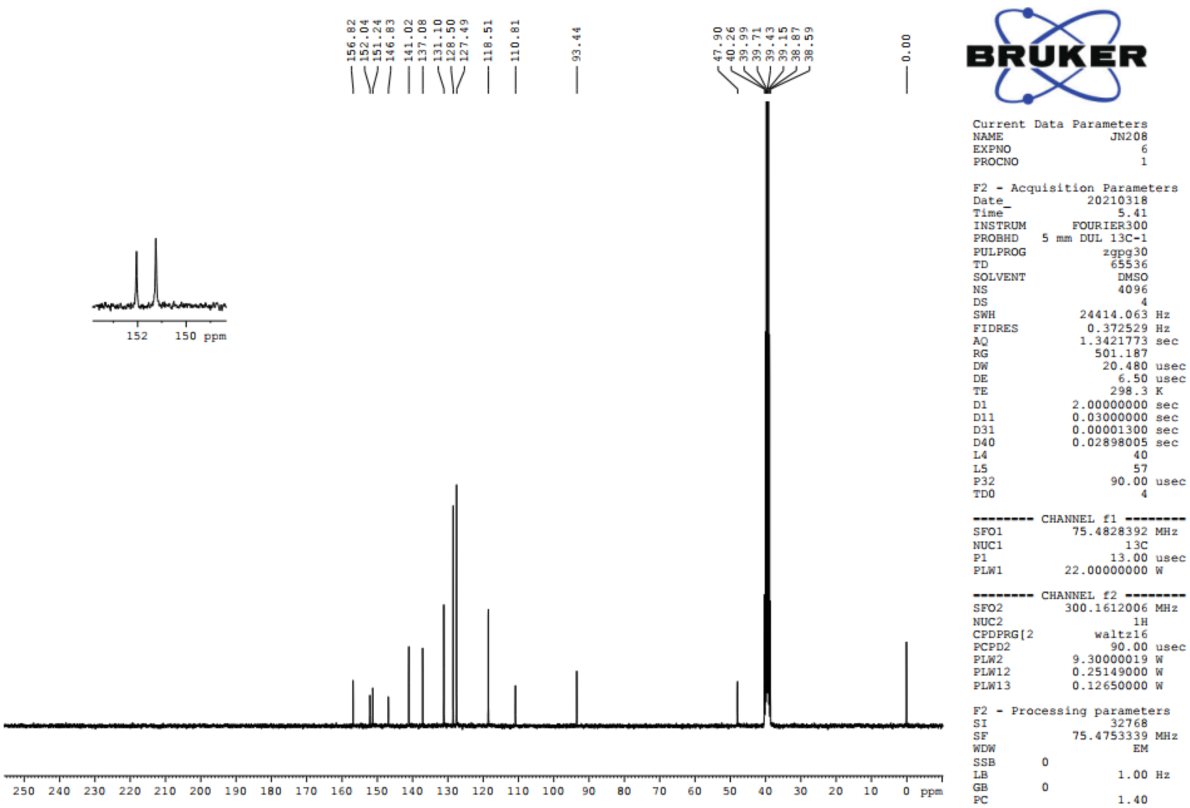

5-Benzyl-3-(4-methylphenylamino)-1,5-dihydro-4*H*-pyrazolo[3,4-*d*]pyrimidin-4-one (4d)

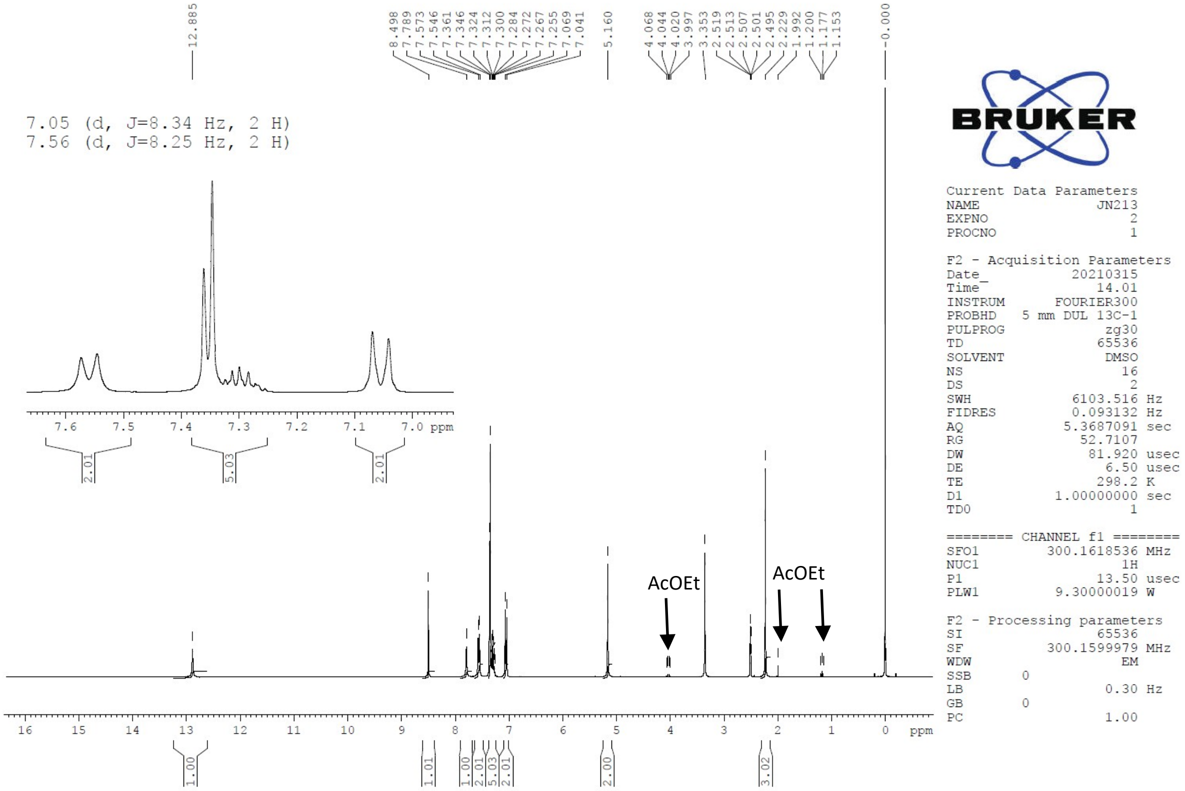

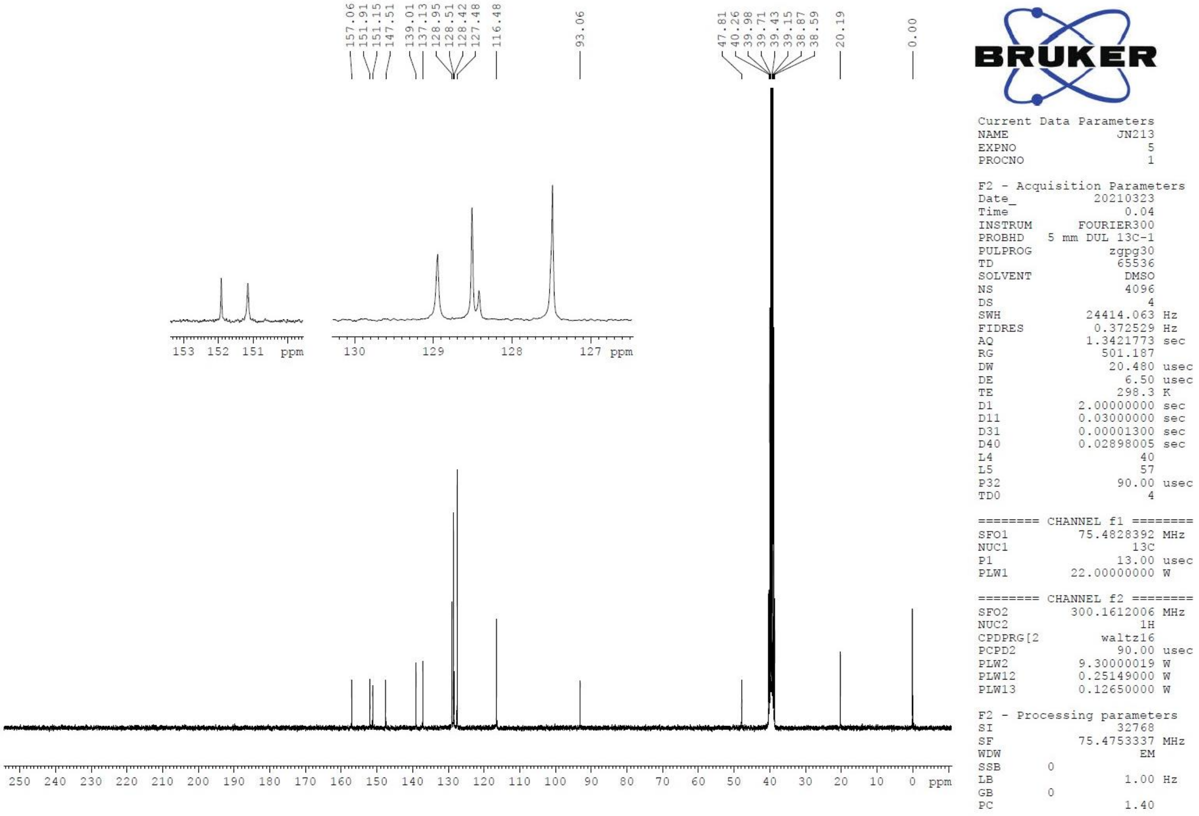

5-Benzyl-3-(4-methoxyphenylamino)-1,5-dihydro-4*H*-pyrazolo[3,4-*d*]pyrimidin-4-one (4e)

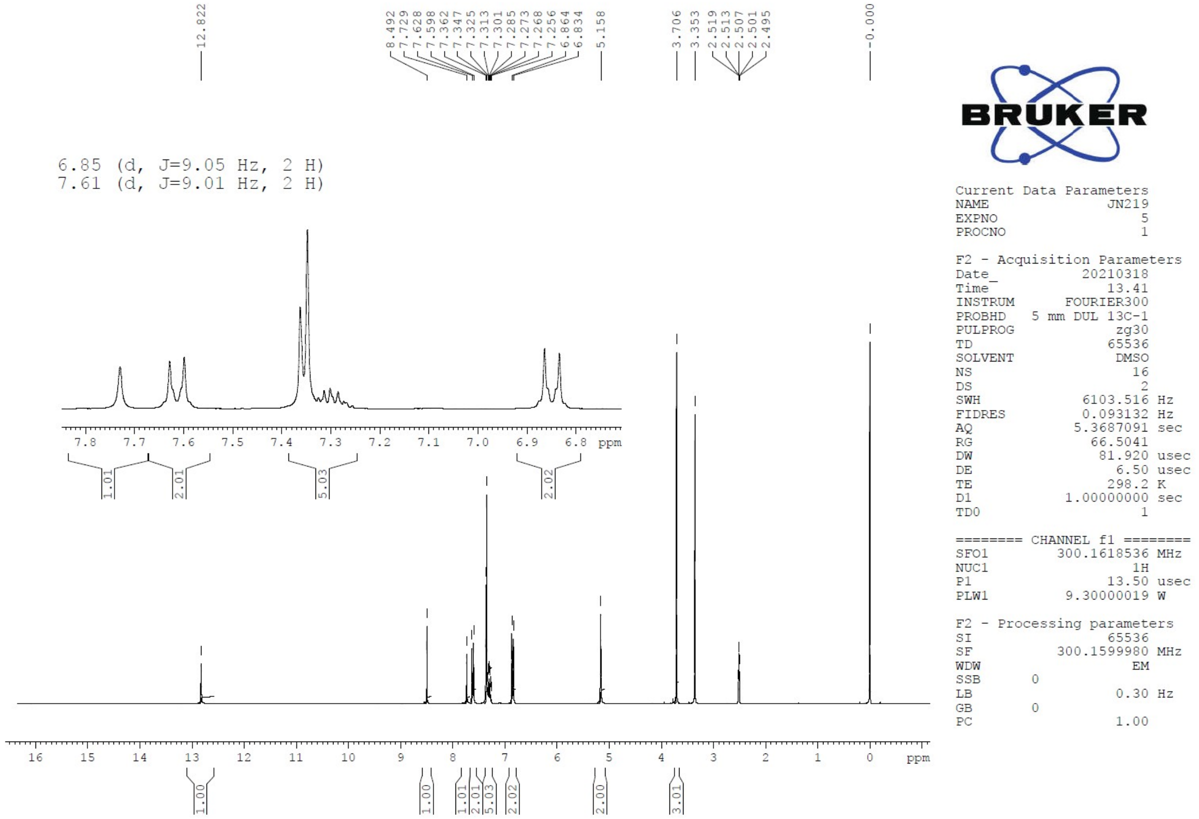

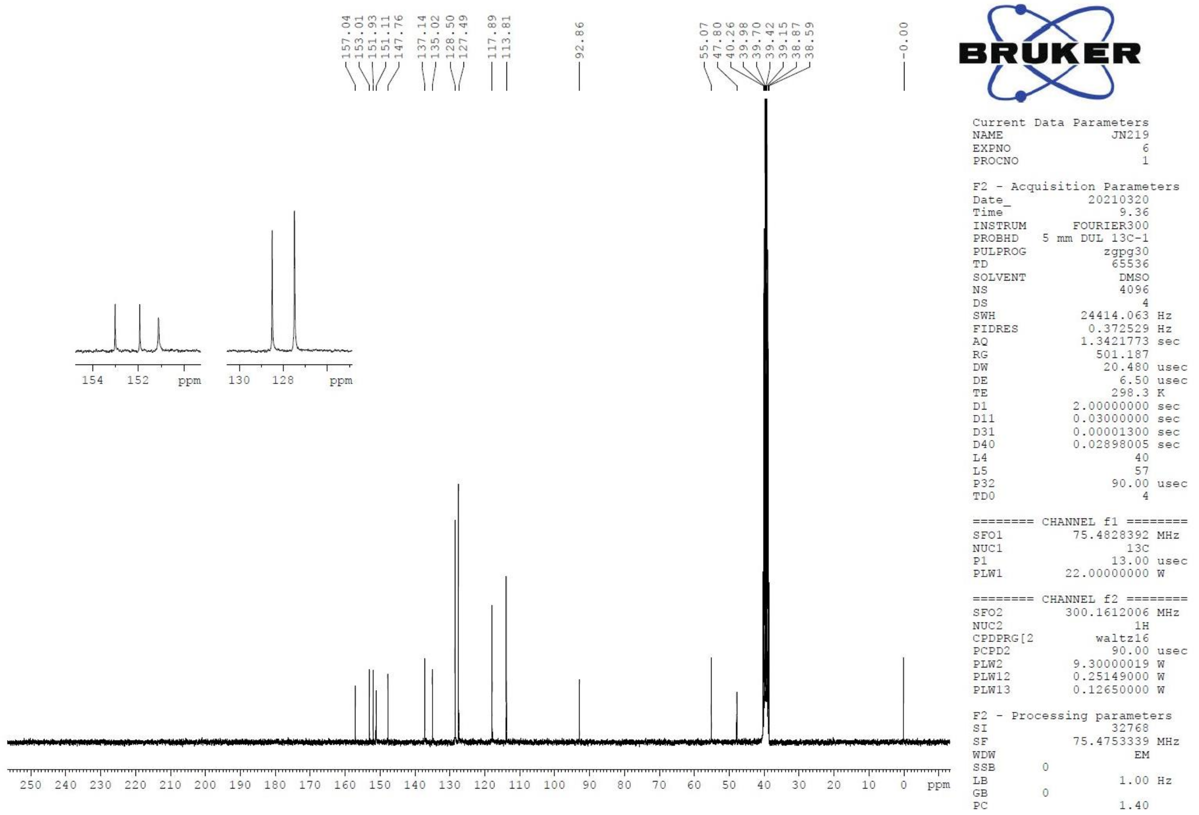

3-(4-Bromophenylamino)-5-(4-fluorobenzyl)-1,5-dihydro-4*H*-pyrazolo[3,4-*d*]pyrimidin-4-one (4f)

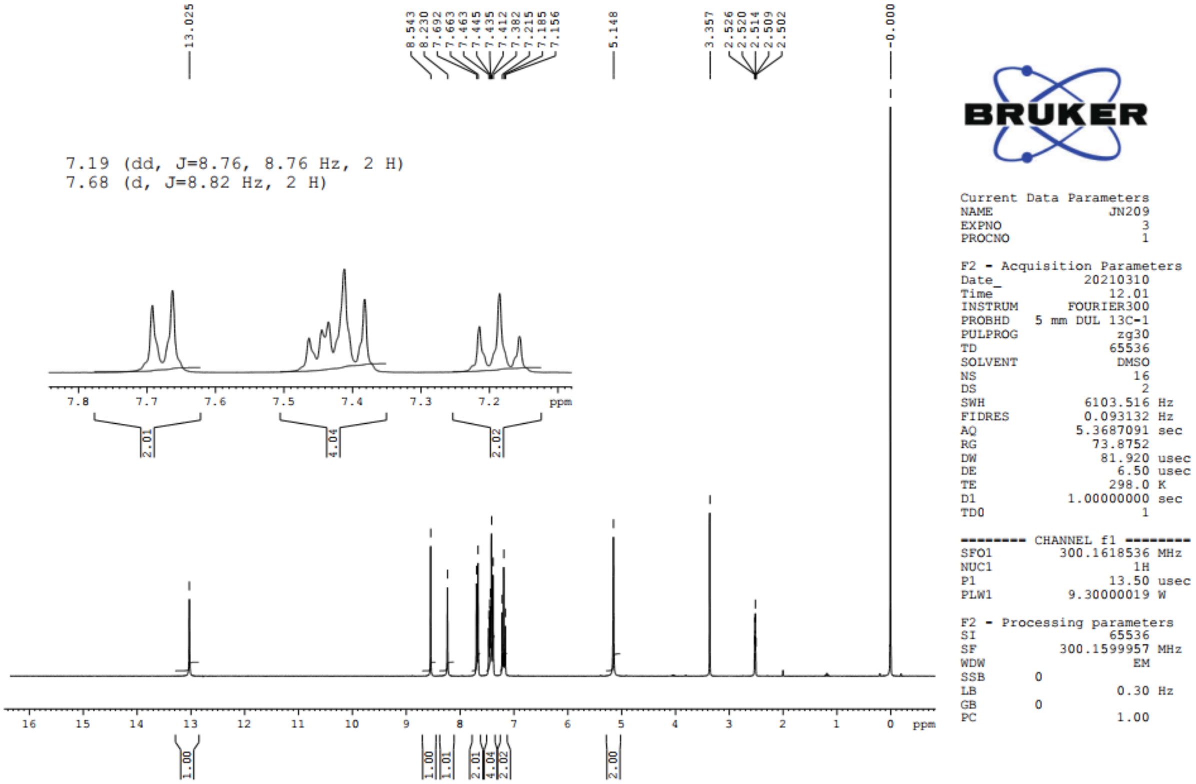

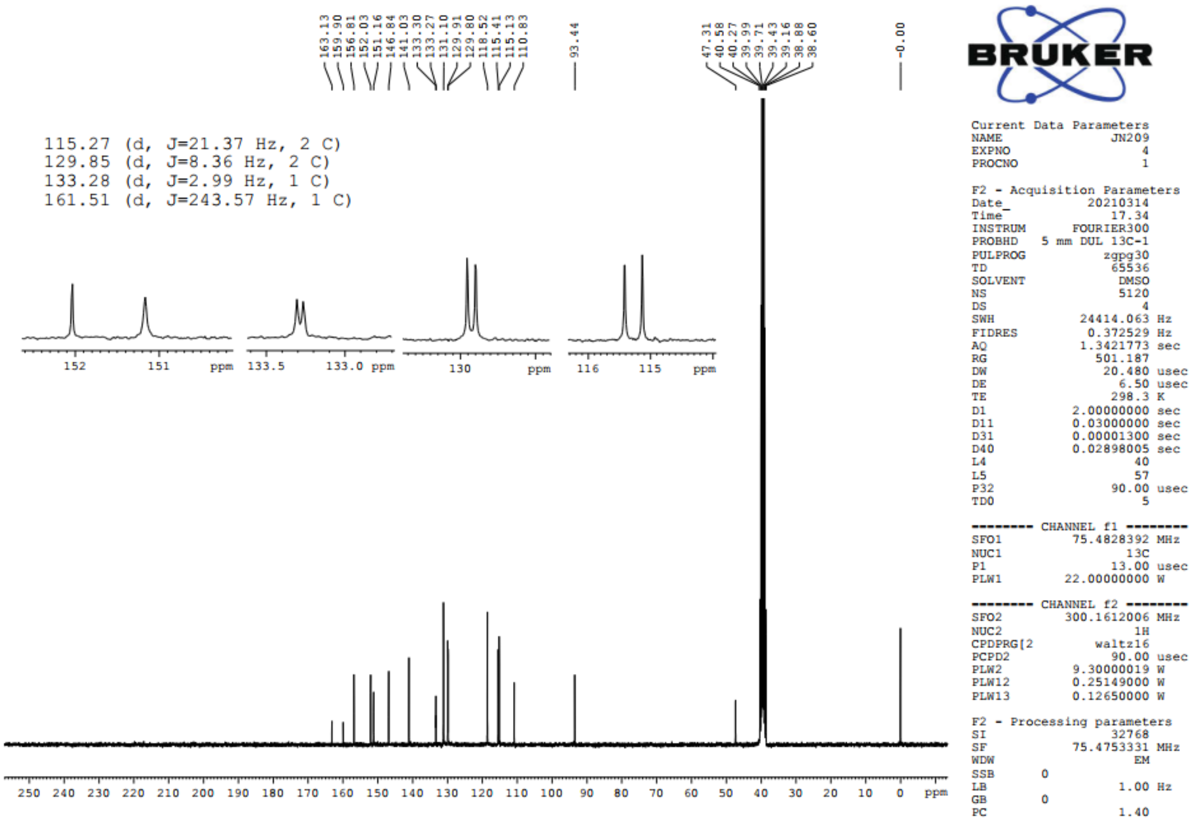

3-(4-Bromophenylamino)-5-(4-chlorobenzyl)-1,5-dihydro-4*H*-pyrazolo[3,4-*d*]pyrimidin-4-one (4g)

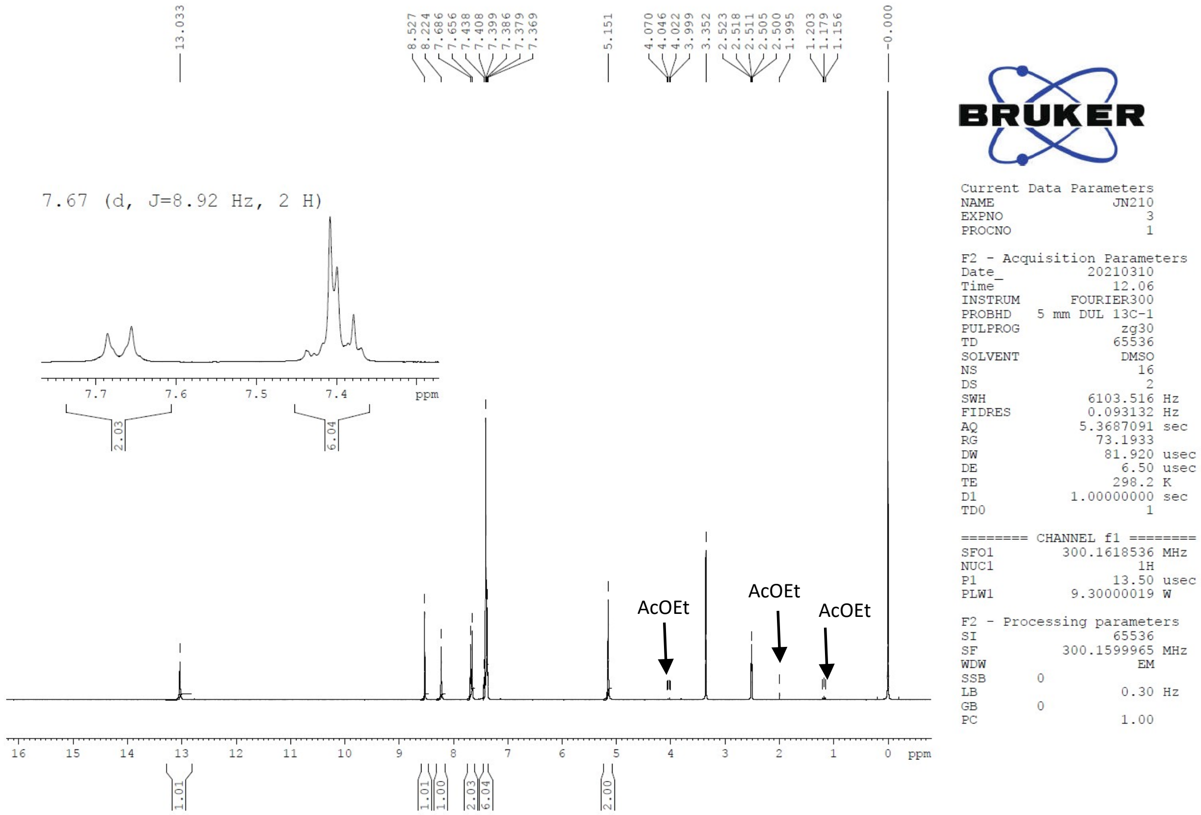

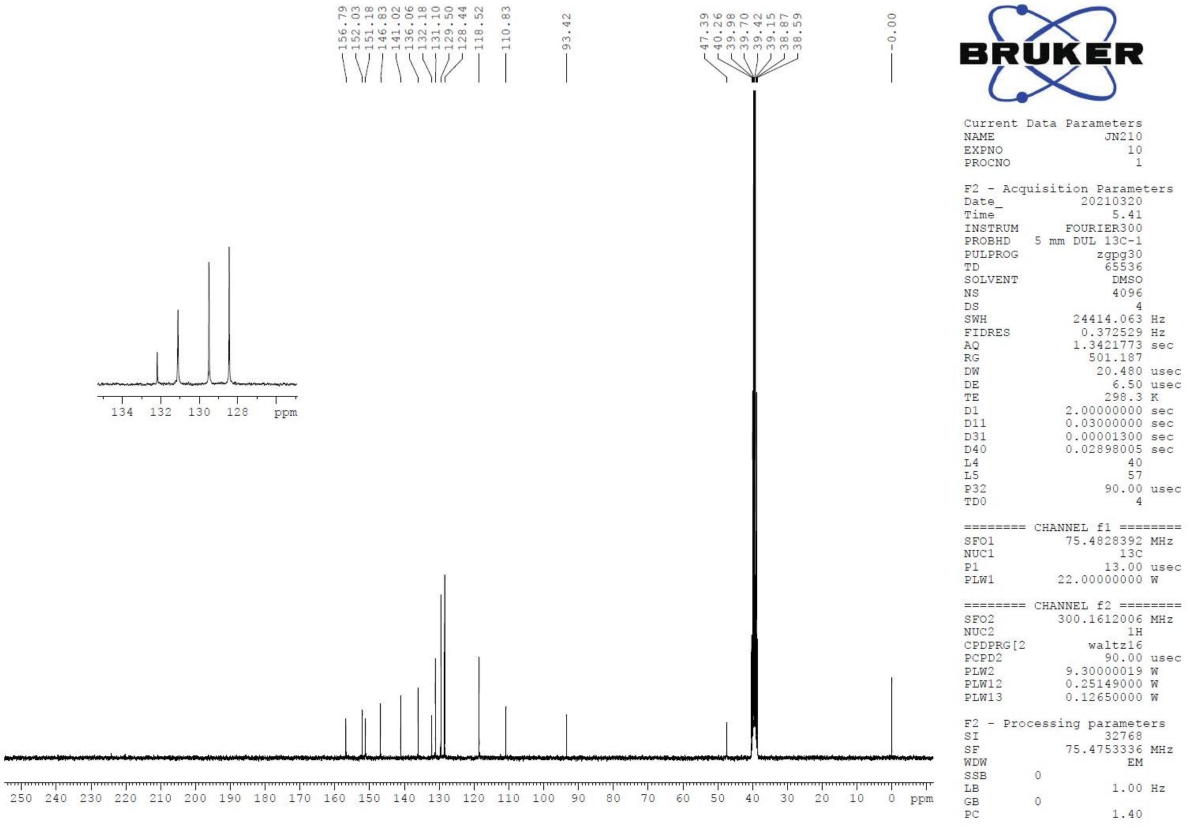

3-(4-Bromophenylamino)-5-(4-methylbenzyl)-1,5-dihydro-4*H*-pyrazolo[3,4-*d*]pyrimidin-4-one (4h)

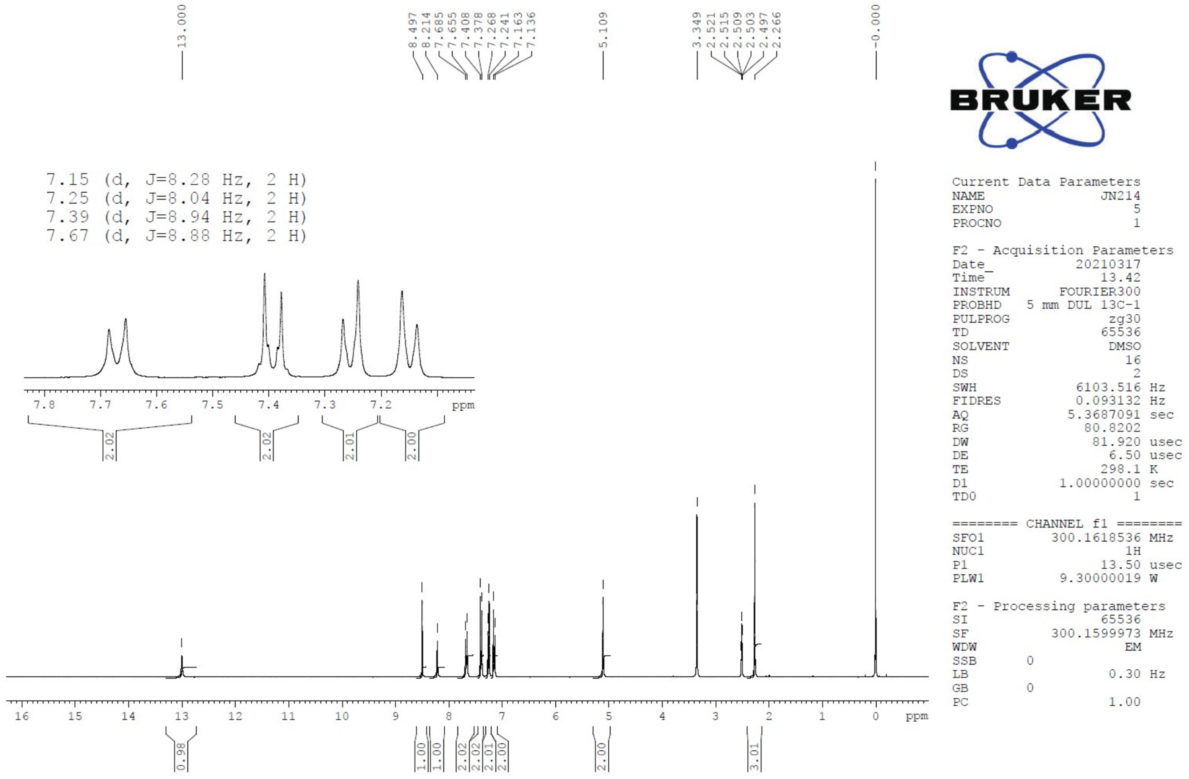

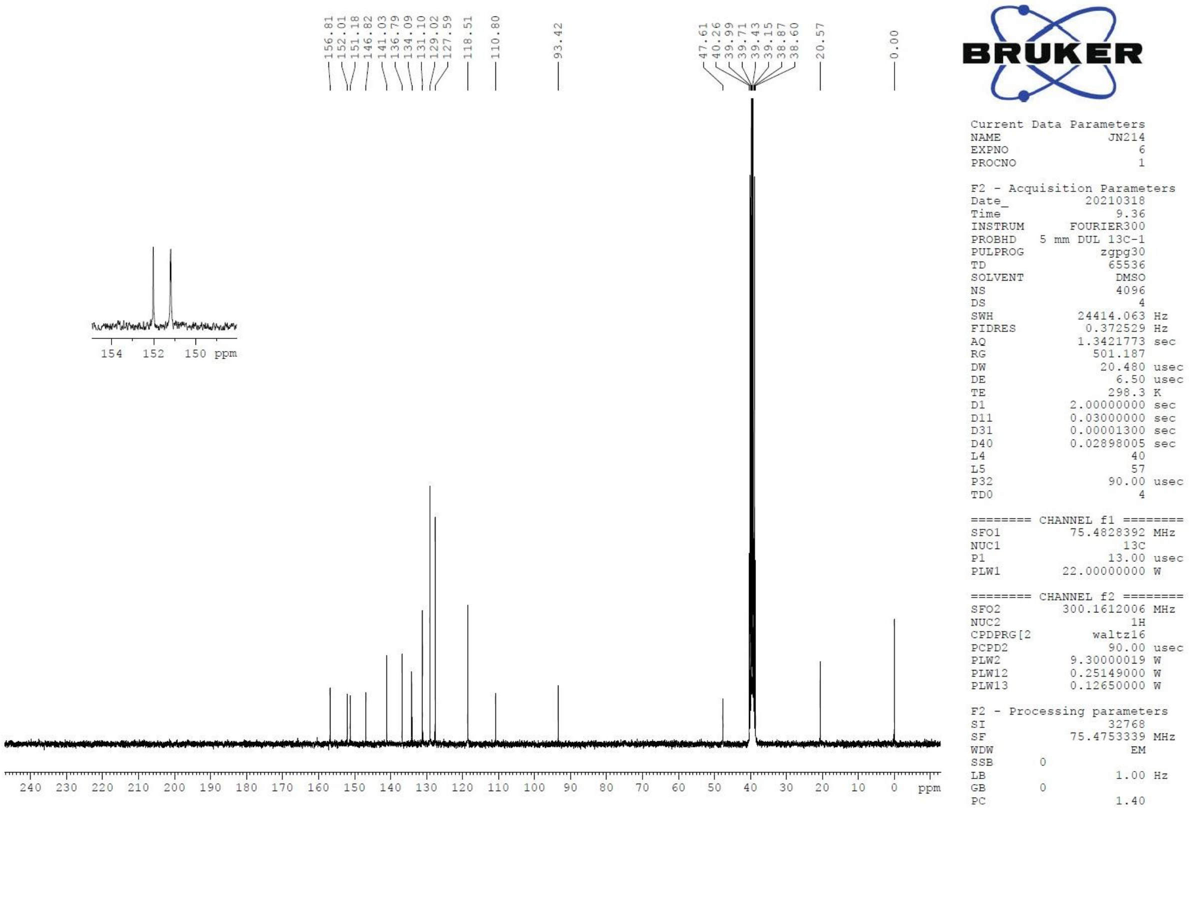

3-(4-Chlorophenylamino)-5-(4-picolyl)-1,5-dihydro-4*H*-pyrazolo[3,4-*d*]pyrimidin-4-one (4i)

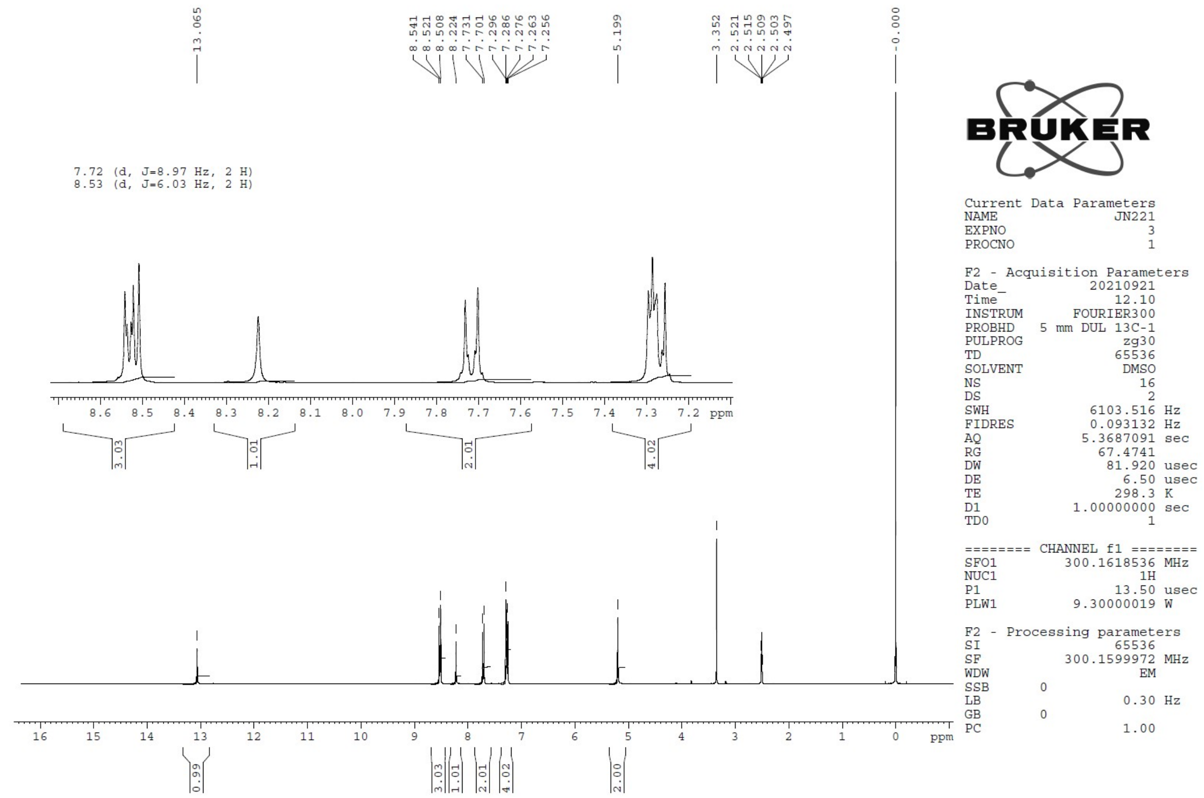

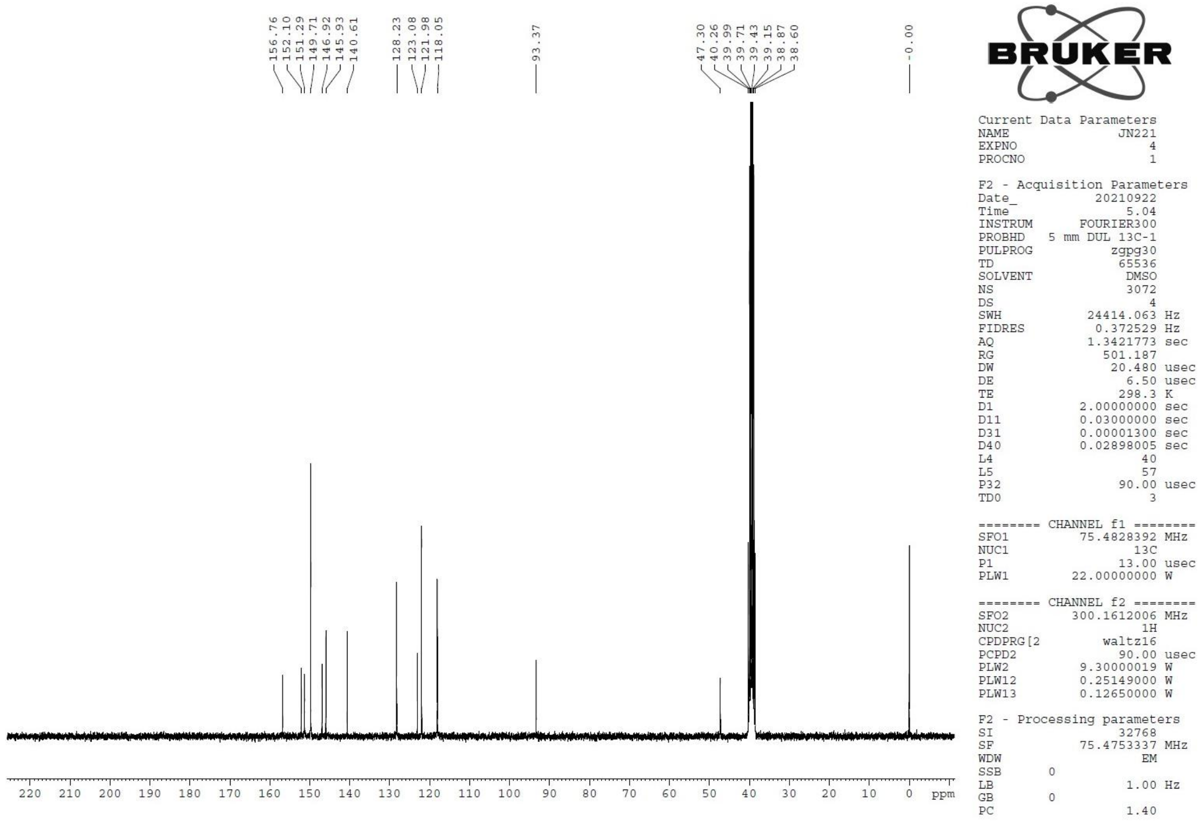

3-(4-Bromophenylamino)-5-(4-picolyl)-1,5-dihydro-4*H*-pyrazolo[3,4-*d*]pyrimidin-4-one (4j)

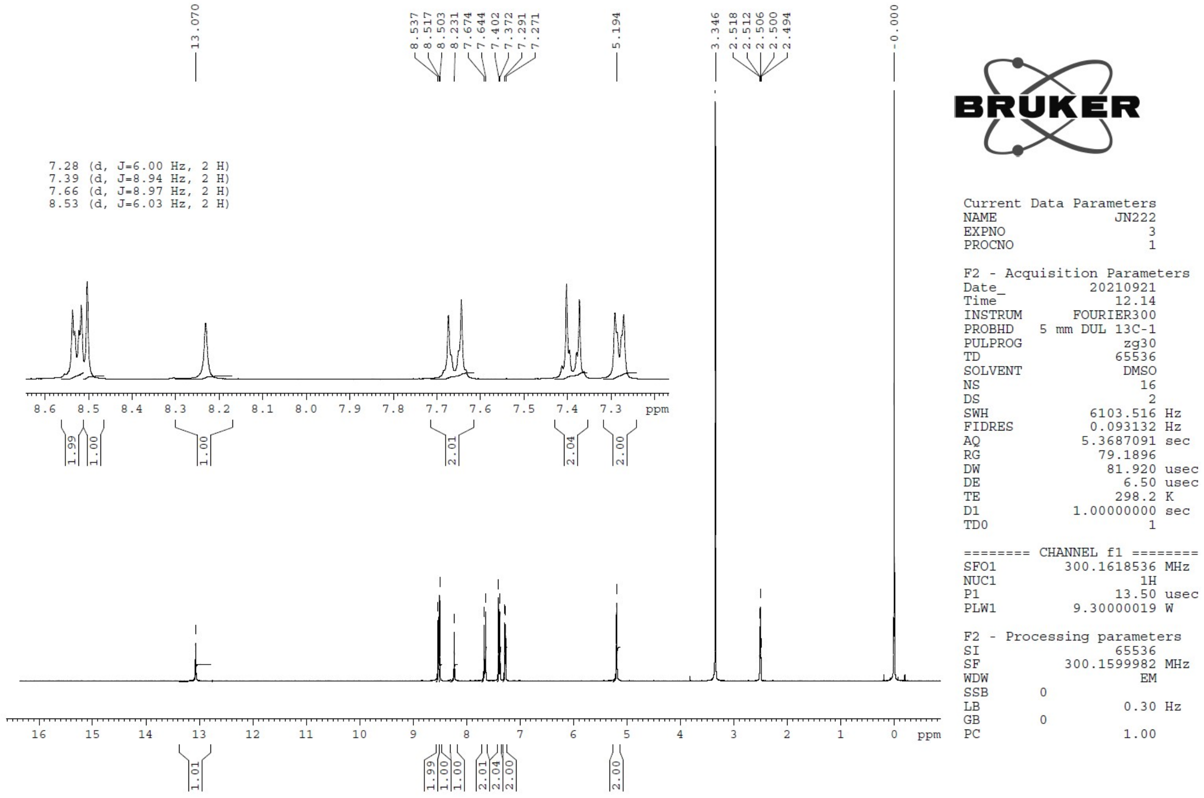

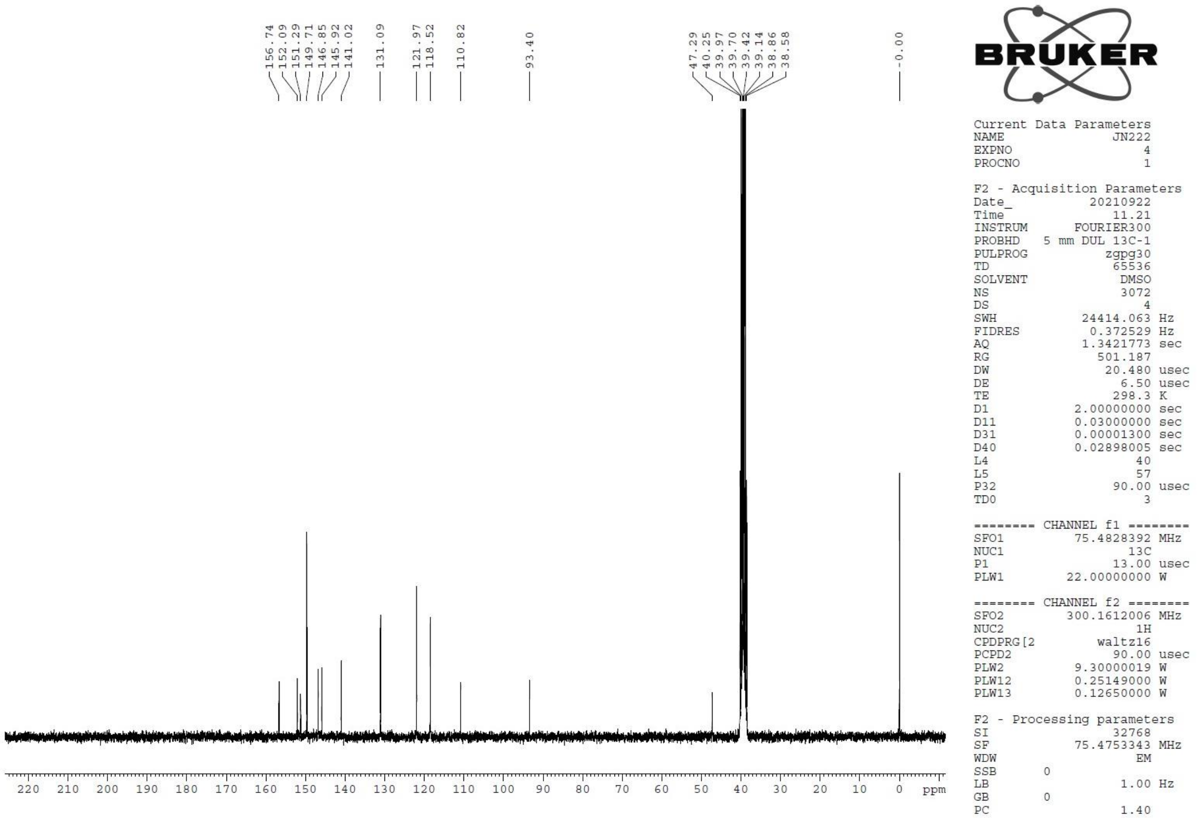

3-(4-Methylphenylamino)-5-(4-picolyl)-1,5-dihydro-4*H*-pyrazolo[3,4-*d*]pyrimidin-4-one (4k)

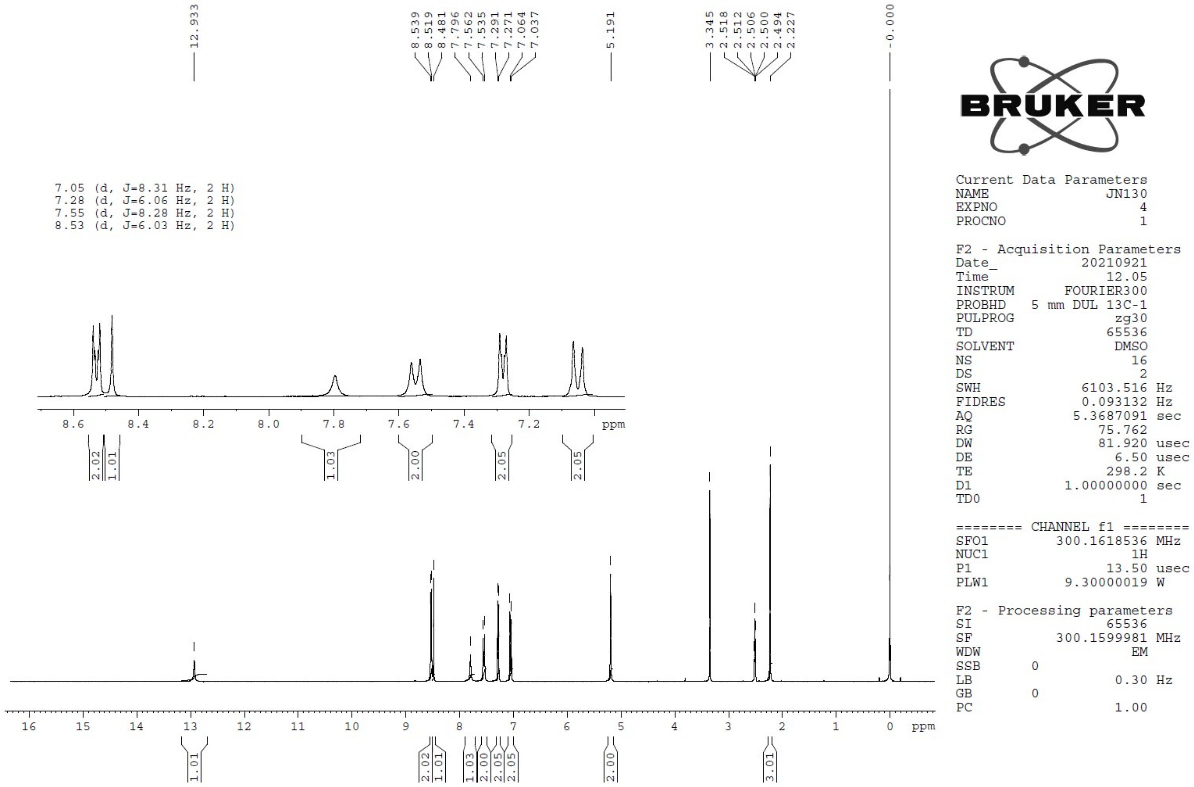

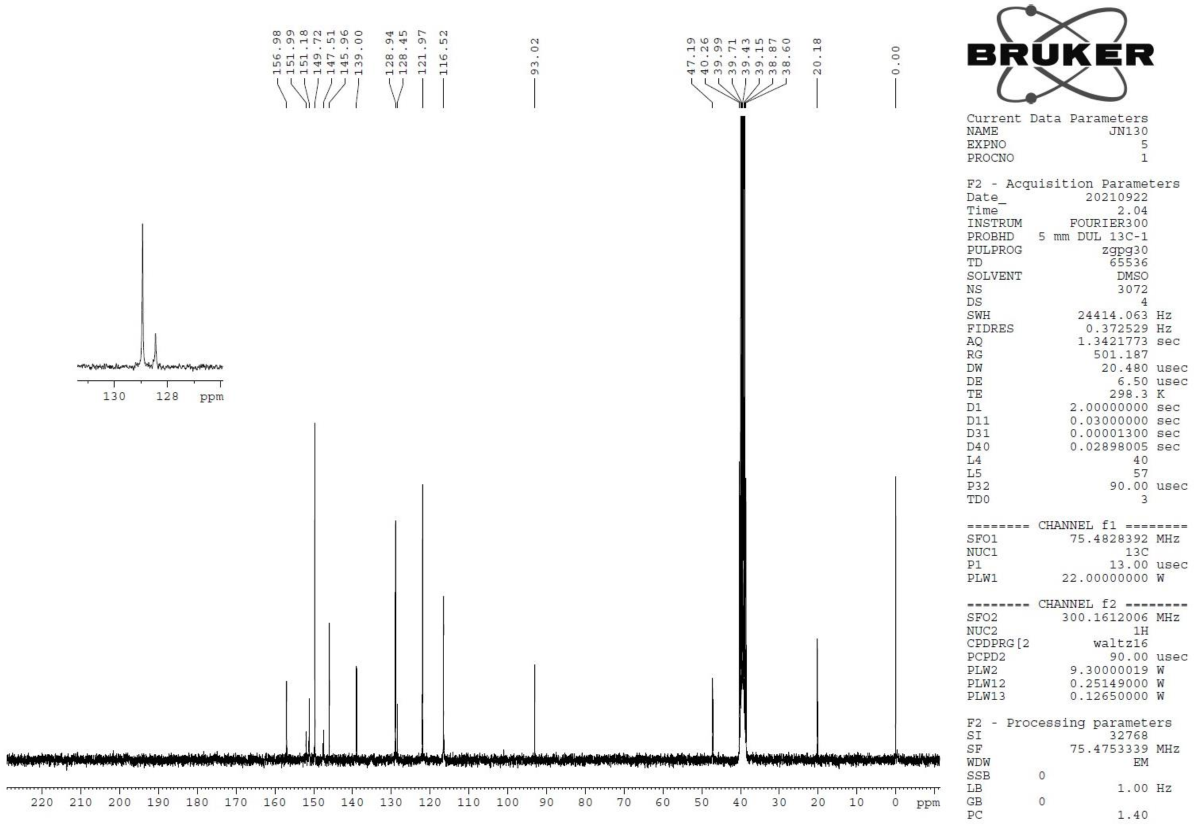

3-(4-Bromophenylamino)-5-(4-phenethyl)-1,5-dihydro-4*H*-pyrazolo[3,4-*d*]pyrimidin-4-one (4l)

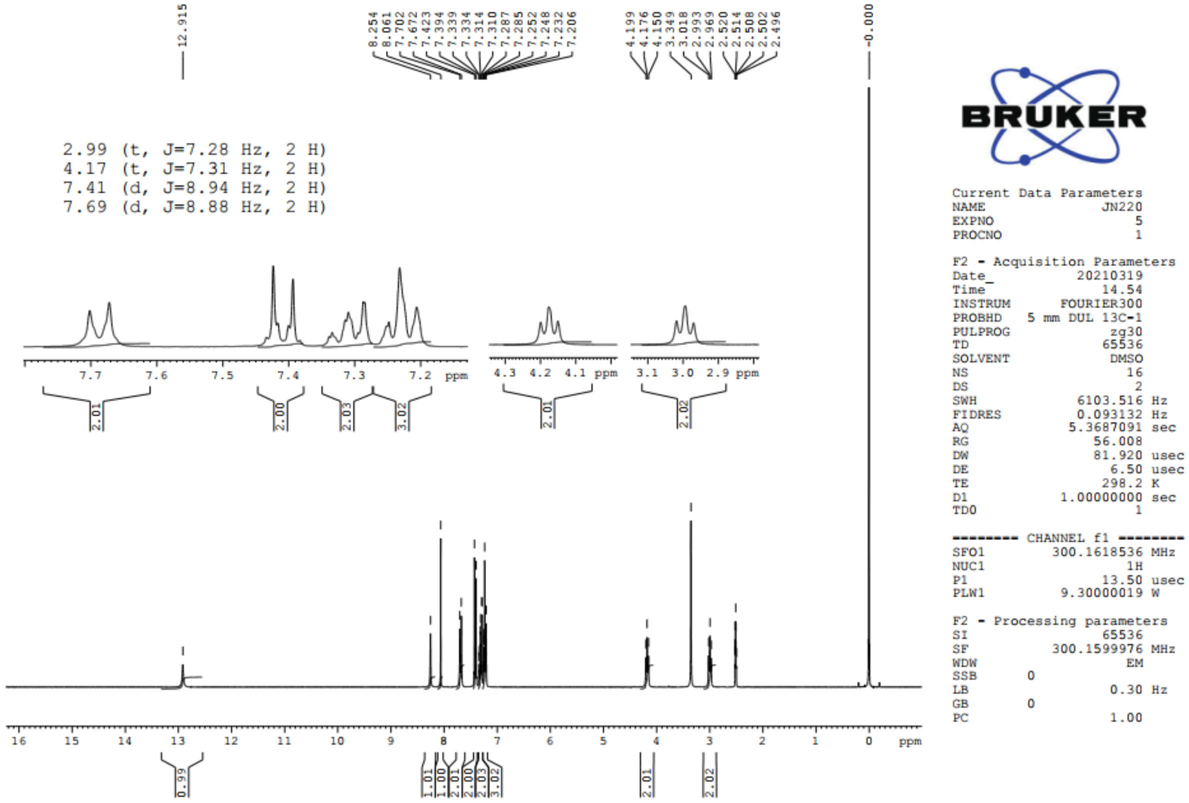

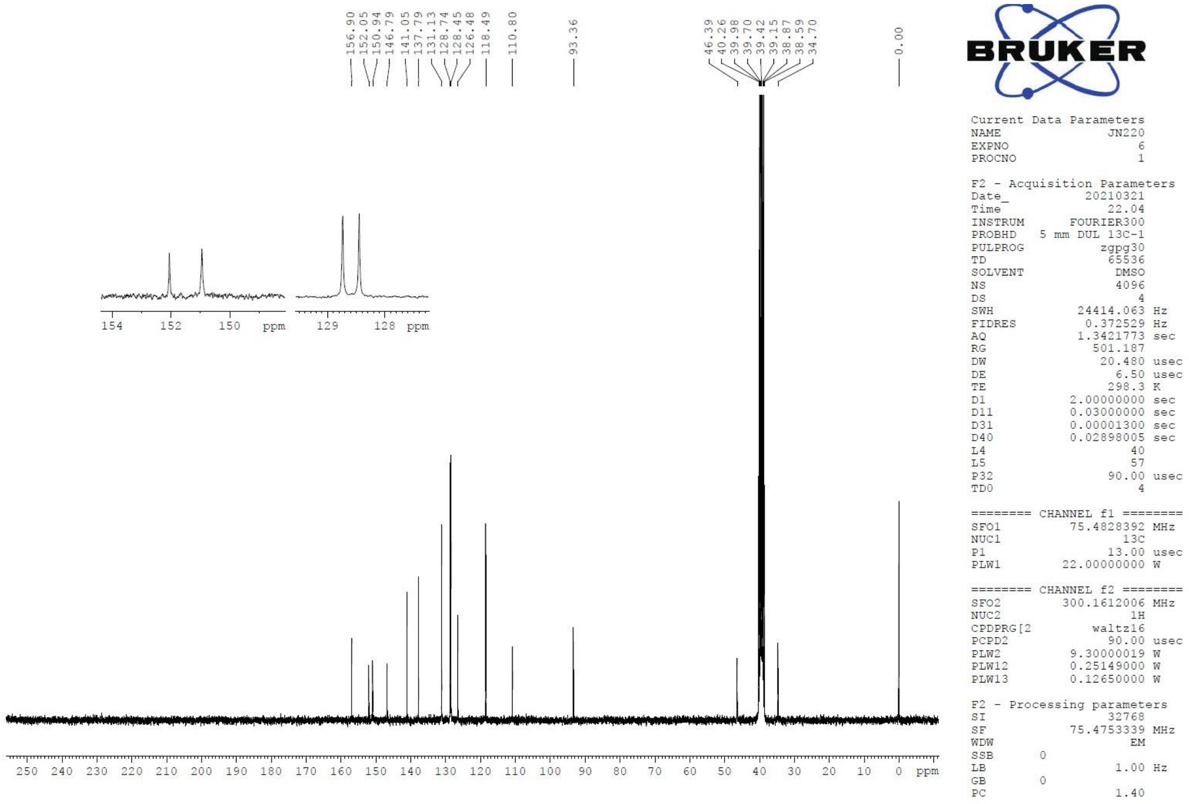

3-(4-Bromophenylamino)-5-(4-methylphenyl)-1,5-dihydro-4*H*-pyrazolo[3,4-*d*]pyrimidin-4-one (4m)

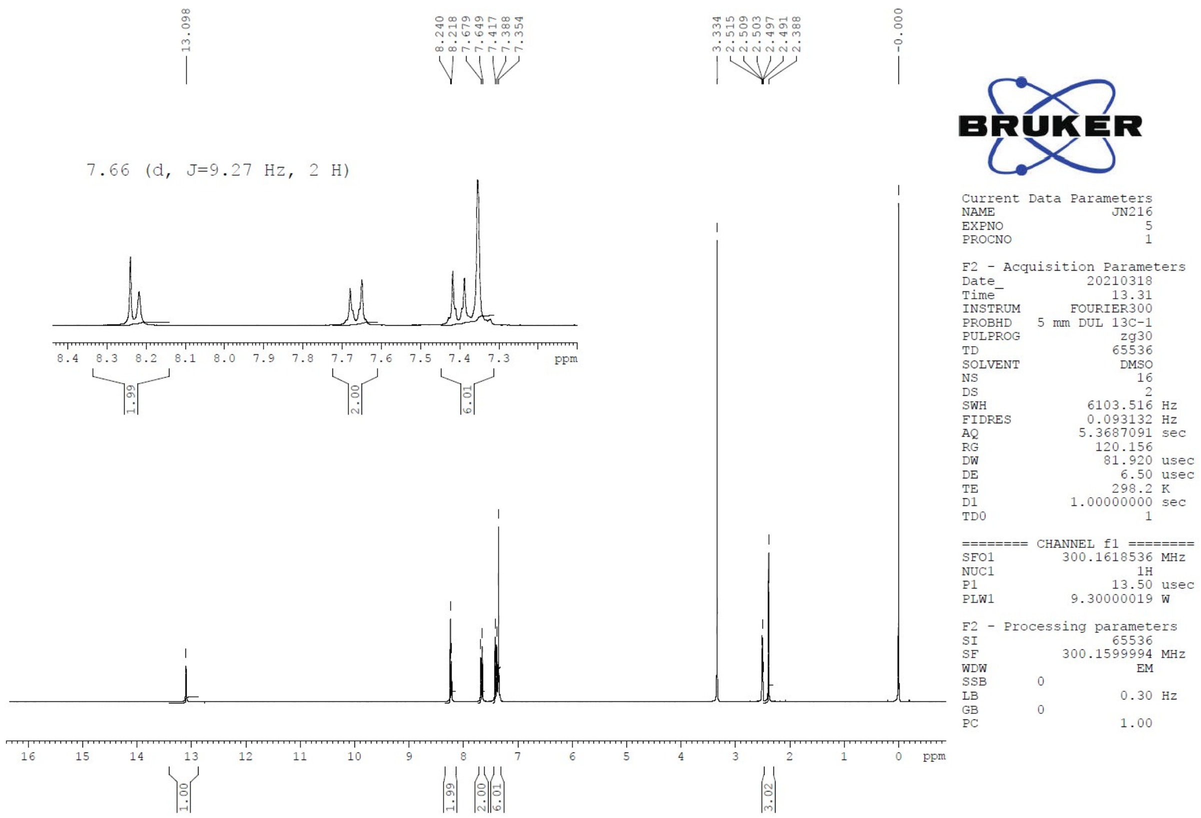

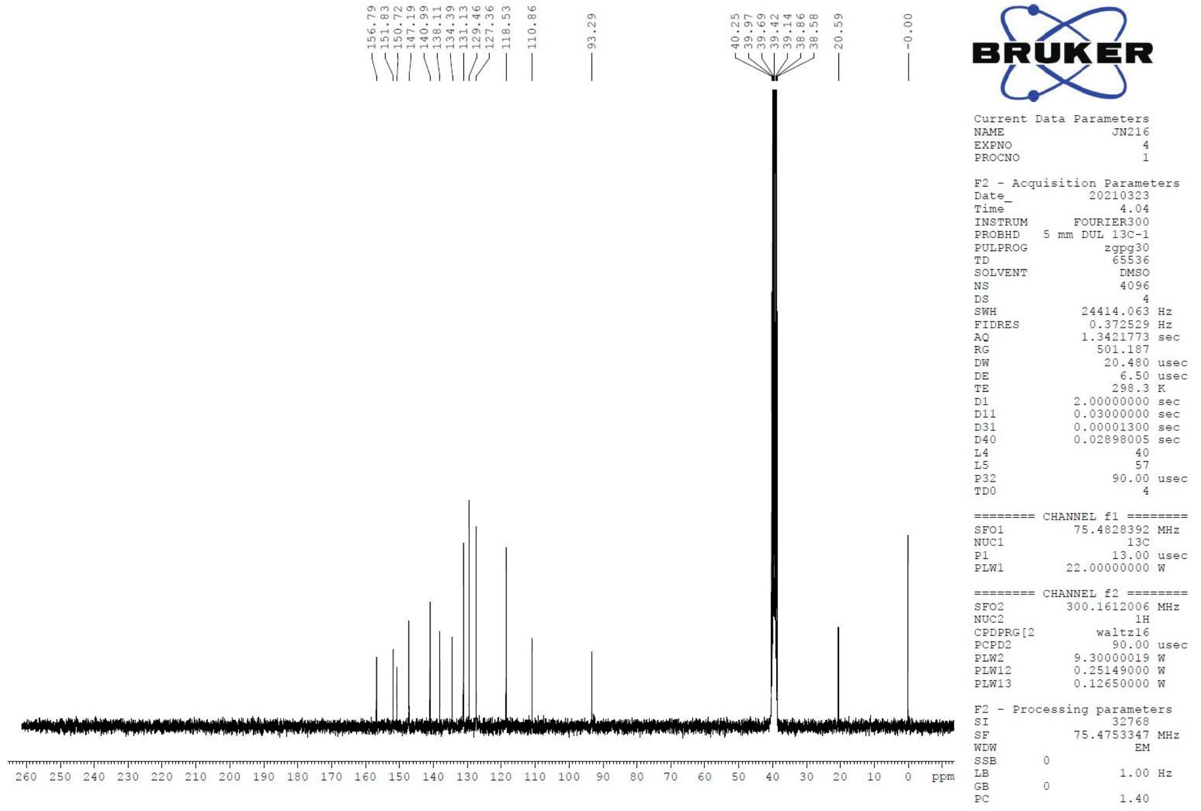

5-(4-Methoxylphenyl)-3-phenylamino-1,5-dihydro-4*H*-pyrazolo[3,4-*d*]pyrimidin-4-one (4n)

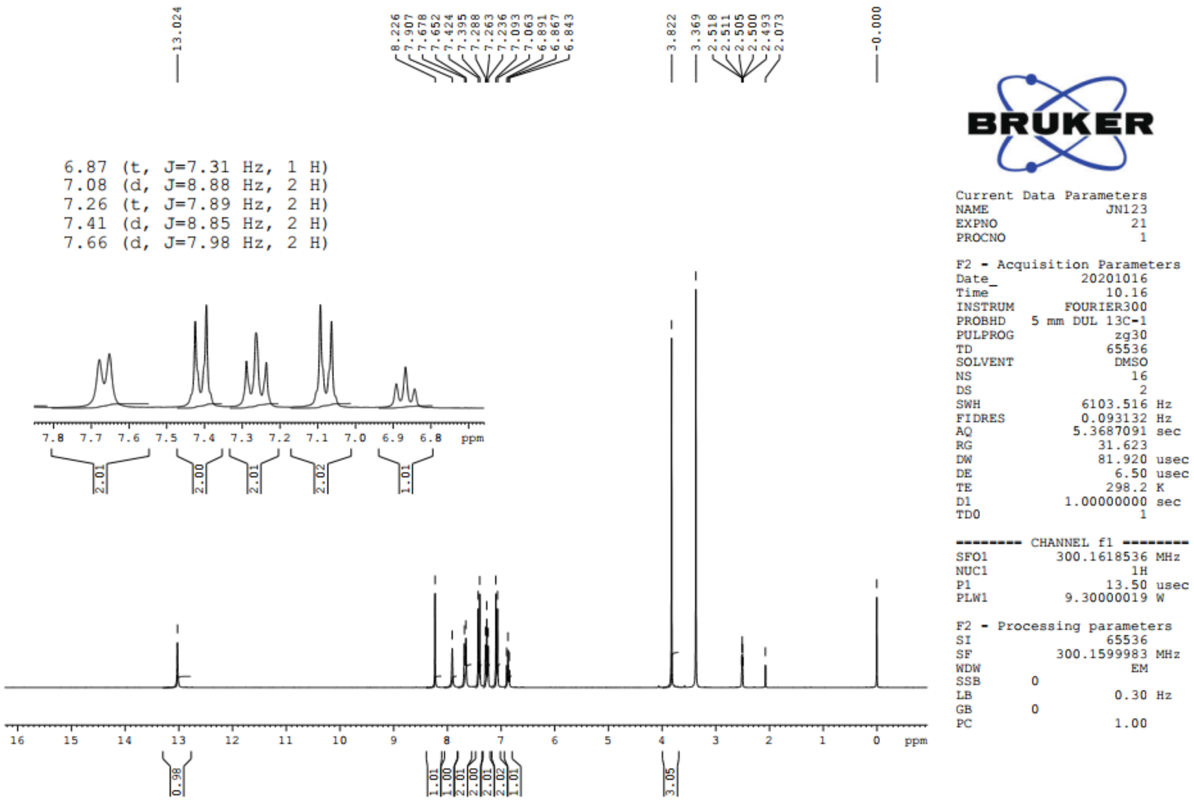

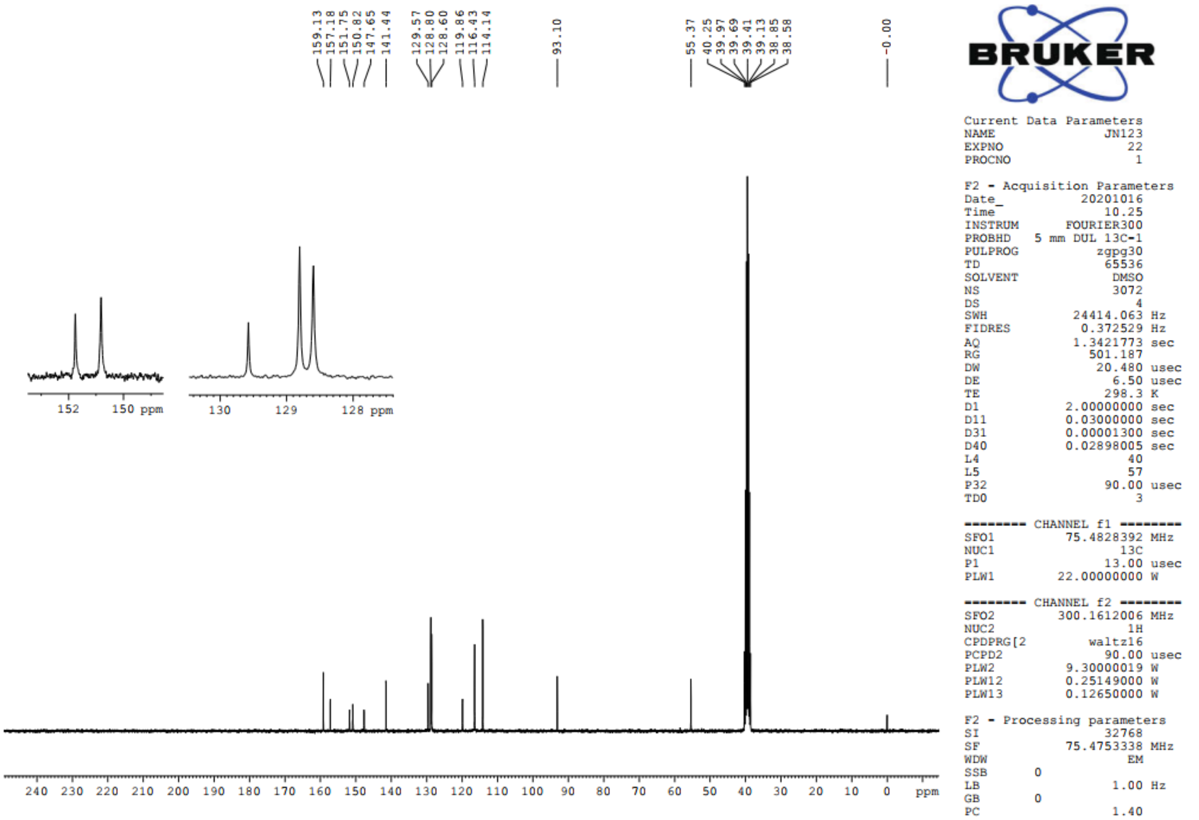

3-(4-Chlorophenylamino)-5-(4-methoxylphenyl)-1,5-dihydro-4*H*-pyrazolo[3,4-*d*]pyrimidin-4-one (4o)

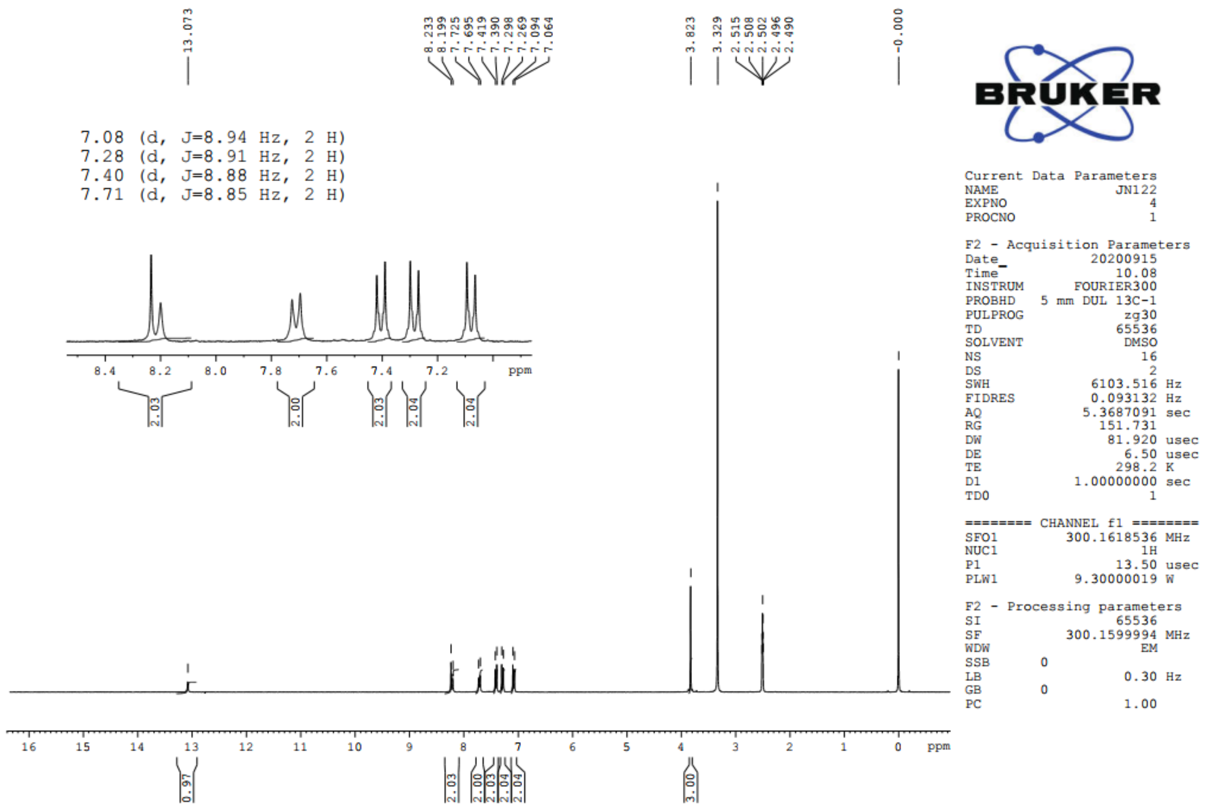

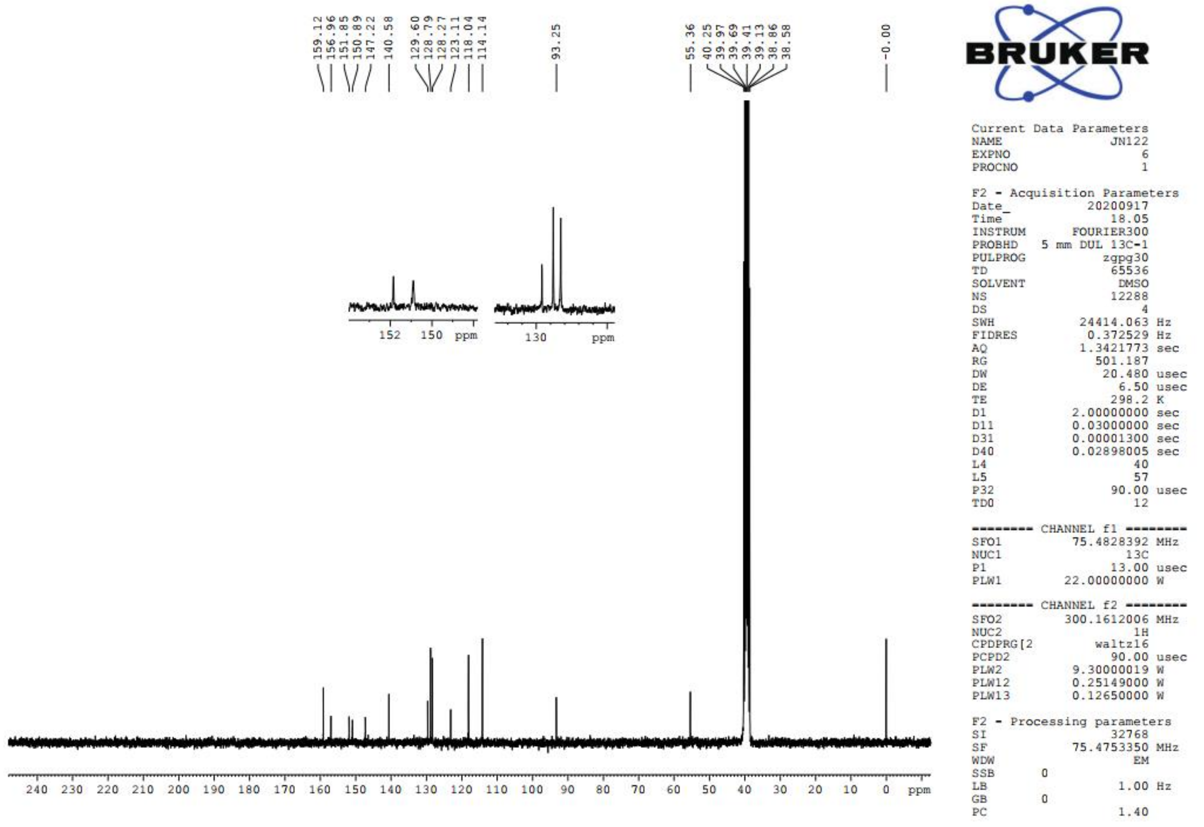

3-(4-Bromophenylamino)-5-(4-methoxylphenyl)-1,5-dihydro-4*H*-pyrazolo[3,4-*d*]pyrimidin-4-one (4p)

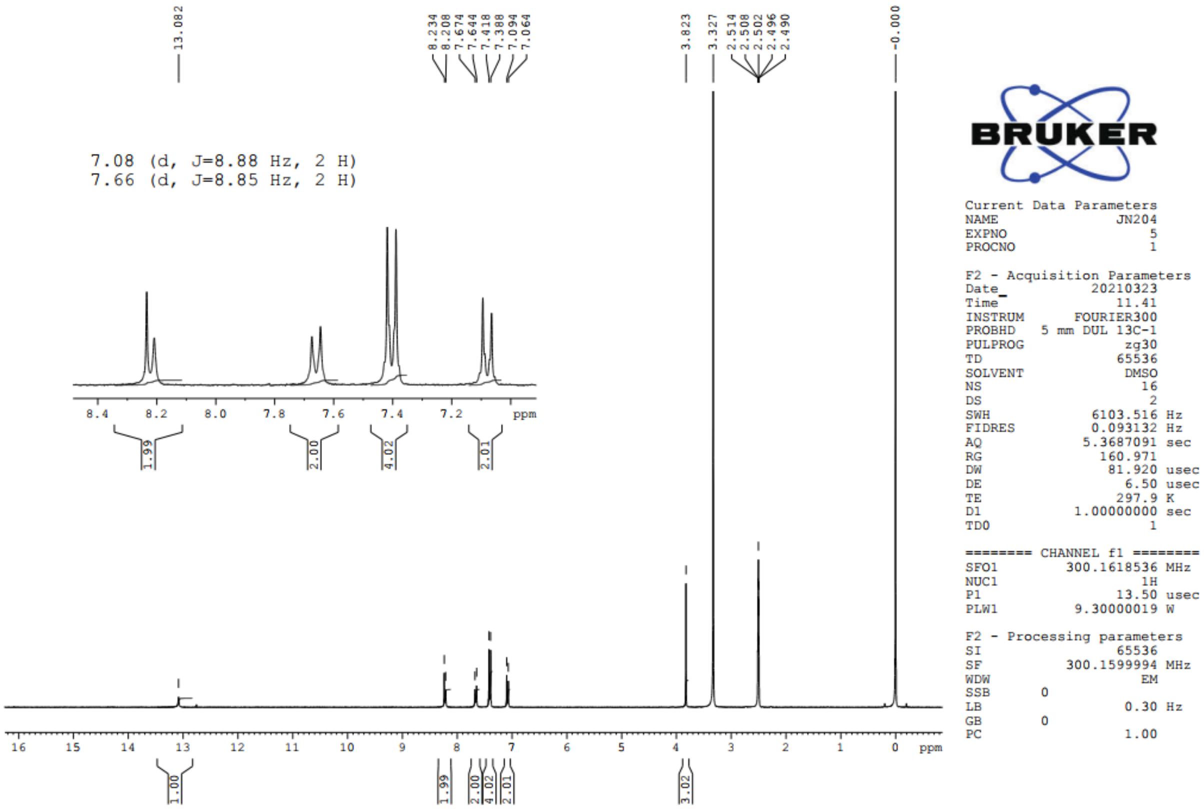

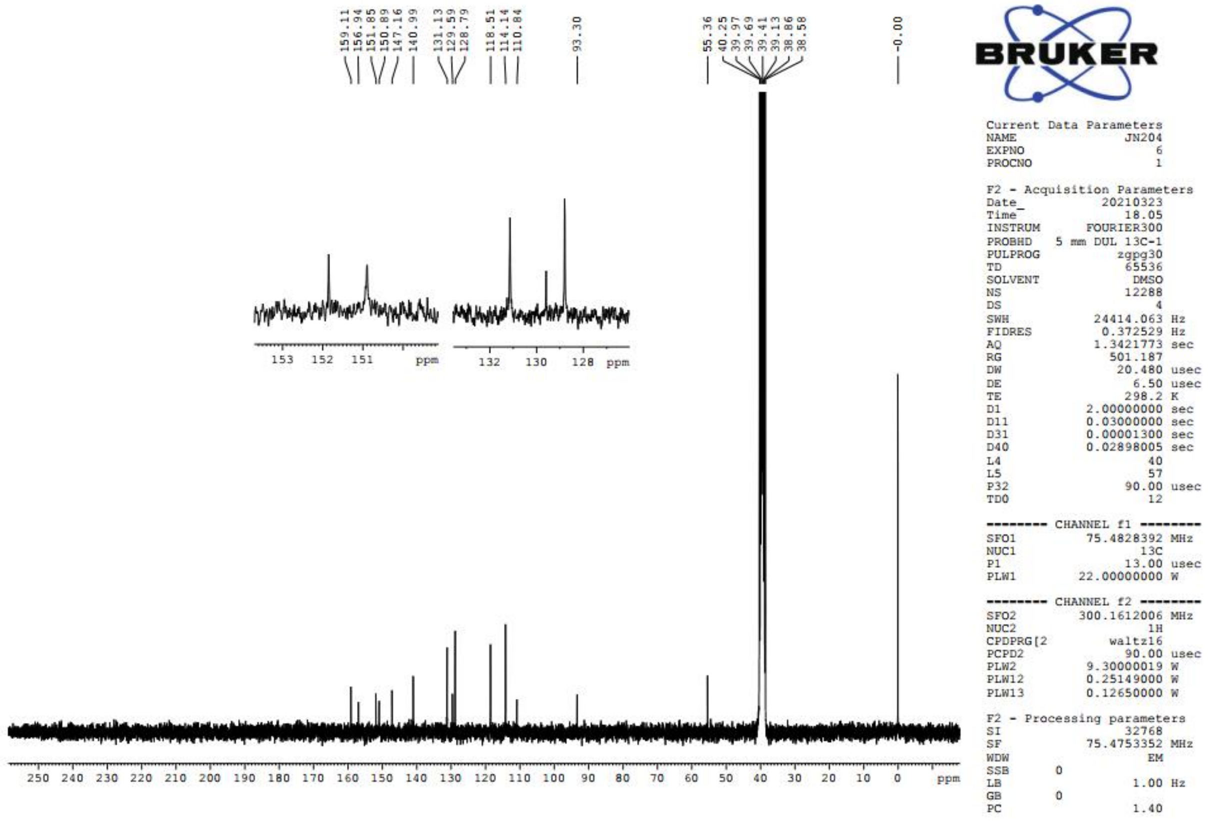

5-(4-Methoxylphenyl)-3-(3-methylphenylamino)-1,5-dihydro-4*H*-pyrazolo[3,4-*d*]pyrimidin-4-one (4q)

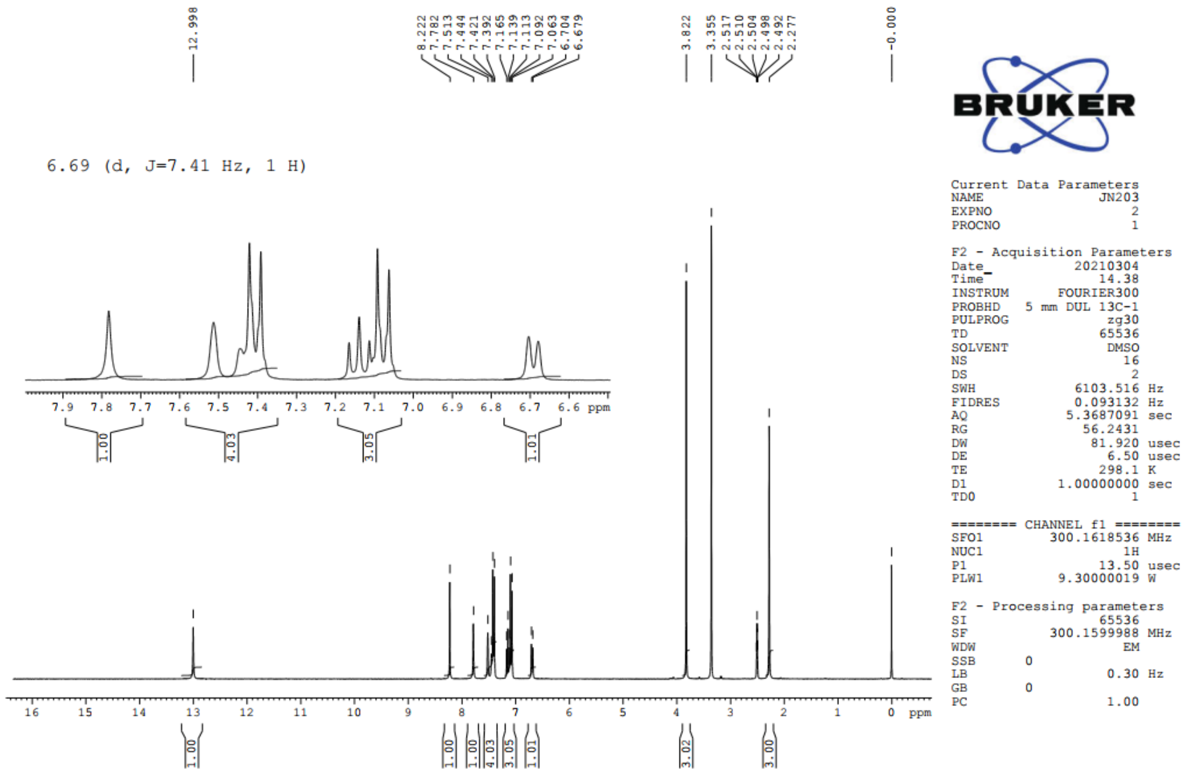

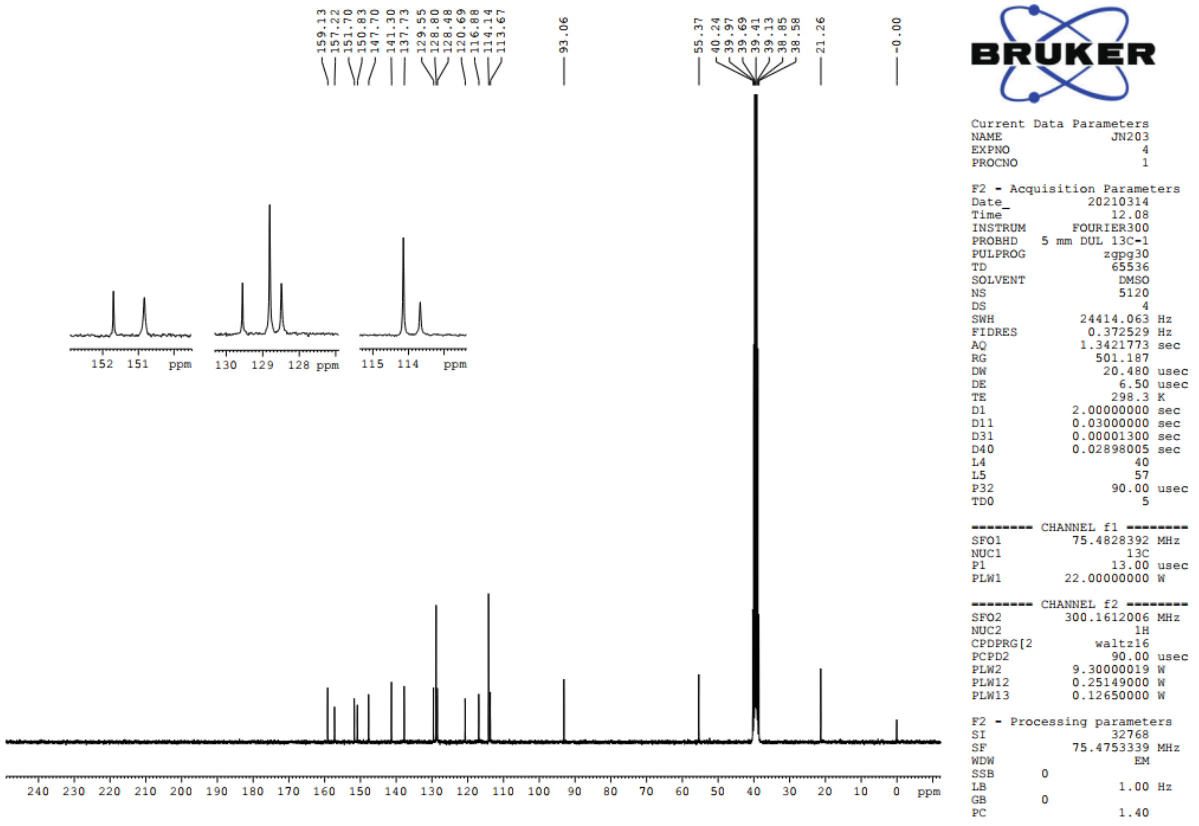

5-(4-Methoxylphenyl)-3-(4-methylphenylamino)-1,5-dihydro-4*H*-pyrazolo[3,4-*d*]pyrimidin-4-one (4r)

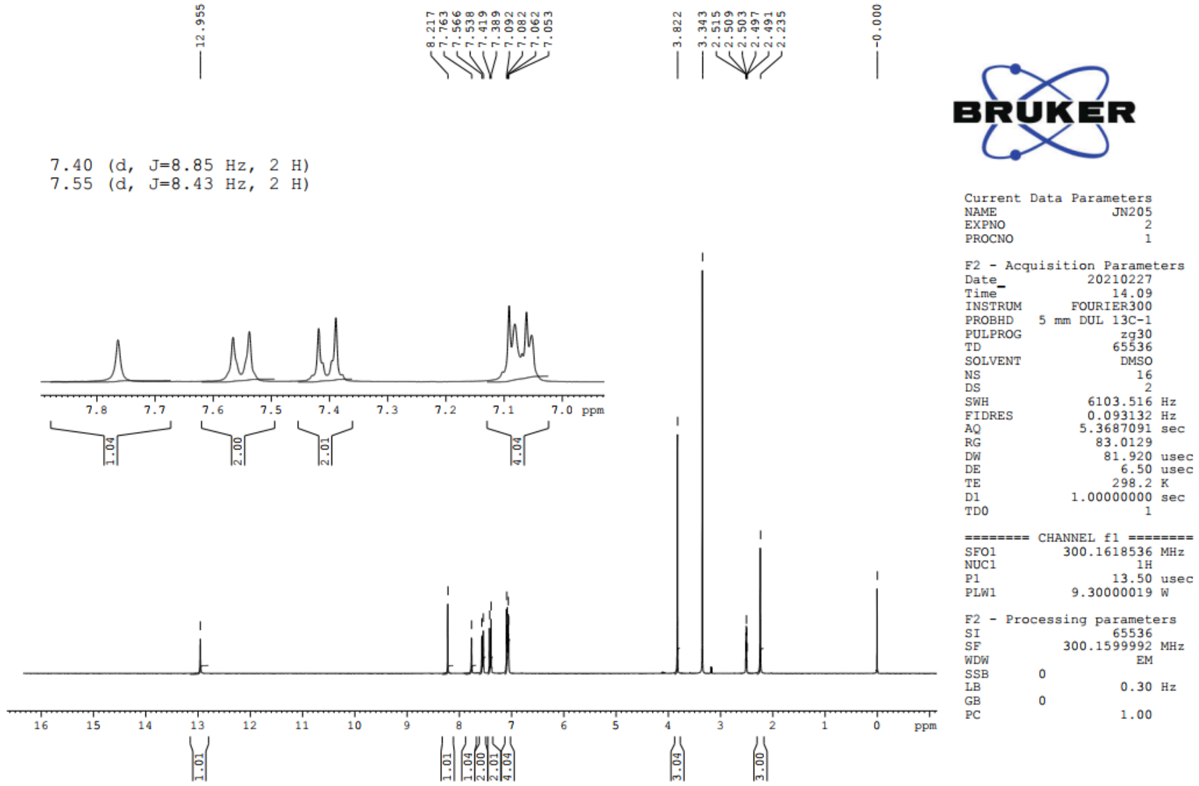

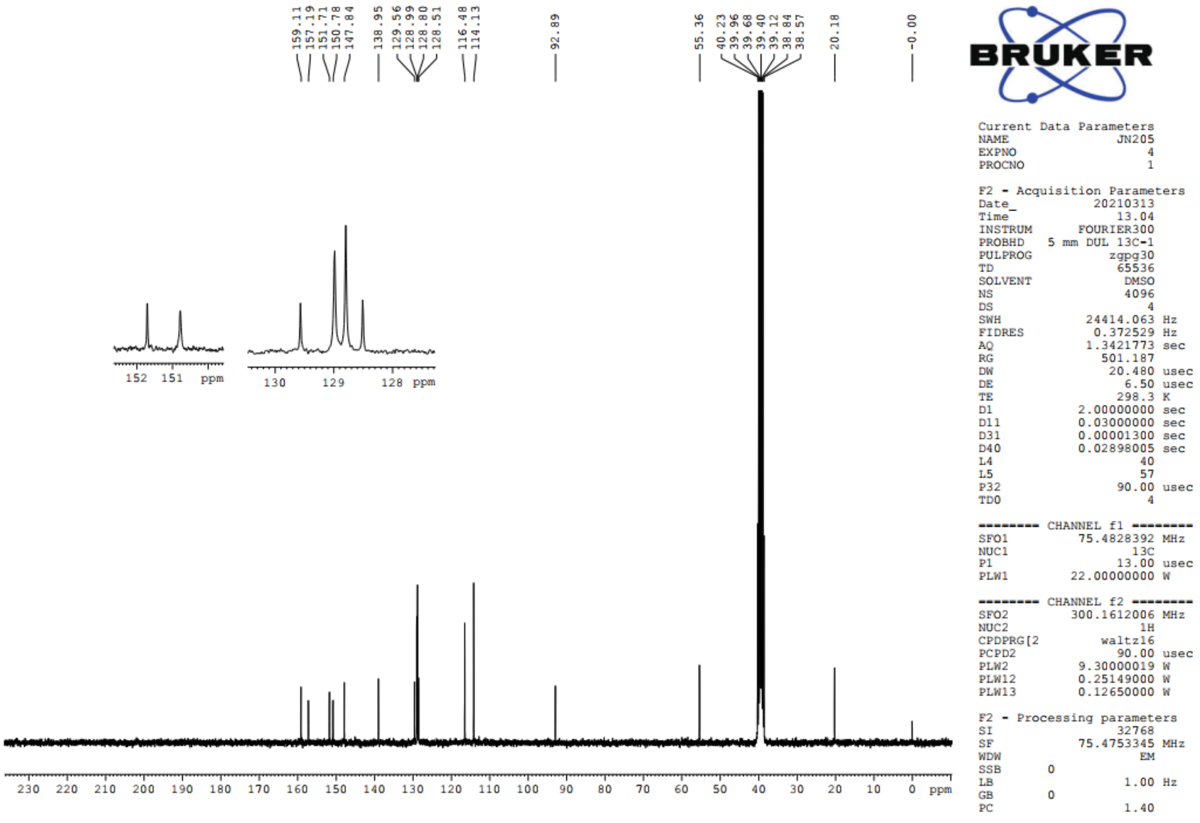

5-(4-Methoxylphenyl)-3-(4-methylphenylamino)-1,5-dihydro-4*H*-pyrazolo[3,4-*d*]pyrimidin-4-one (4r)

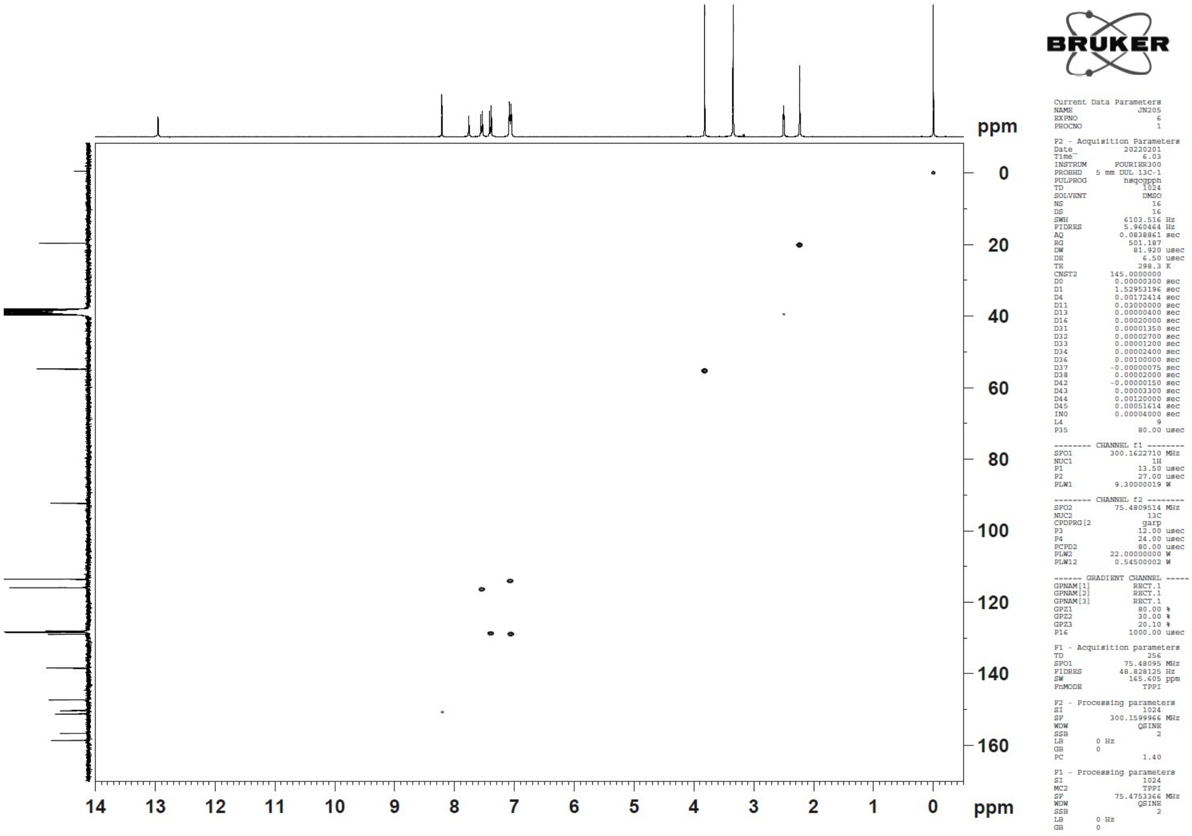

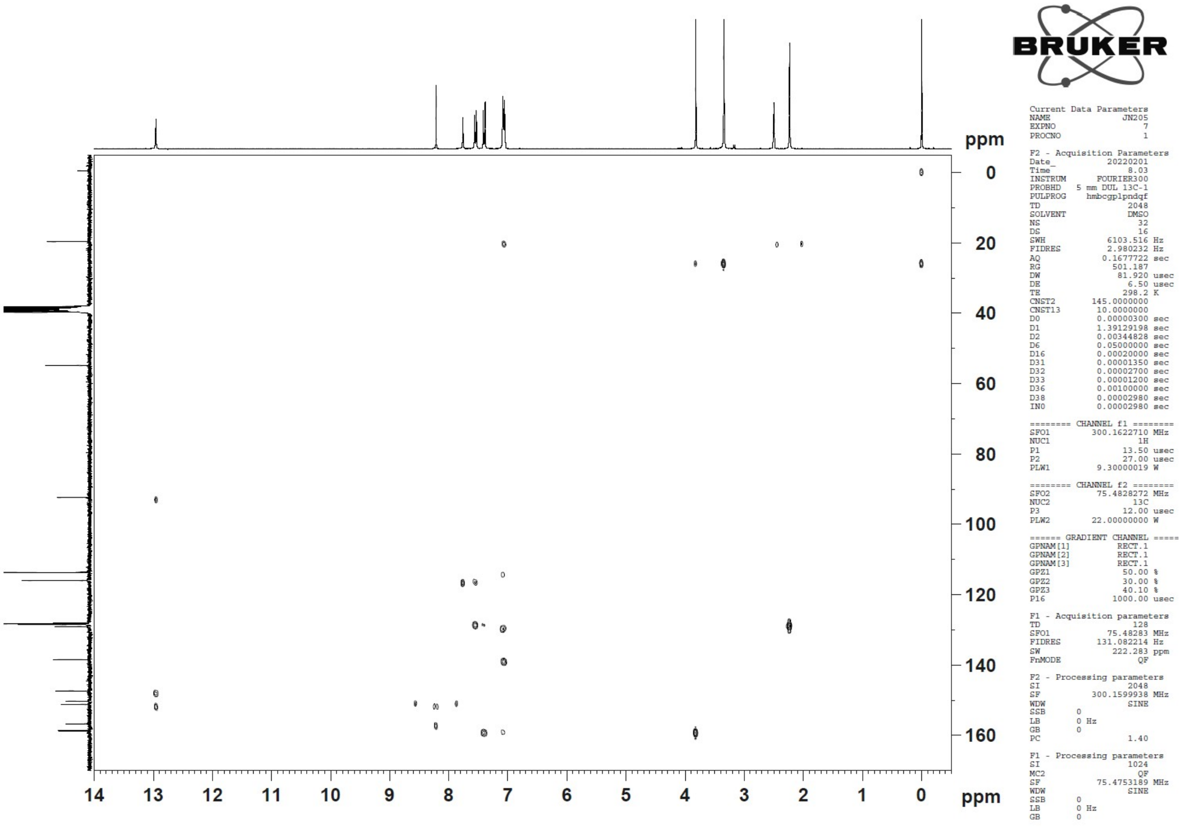

3-(2-Methoxylphenylamino)-5-(4-methoxylphenyl)-1,5-dihydro-4*H*-pyrazolo[3,4-*d*]pyrimidin-4-one (4s)

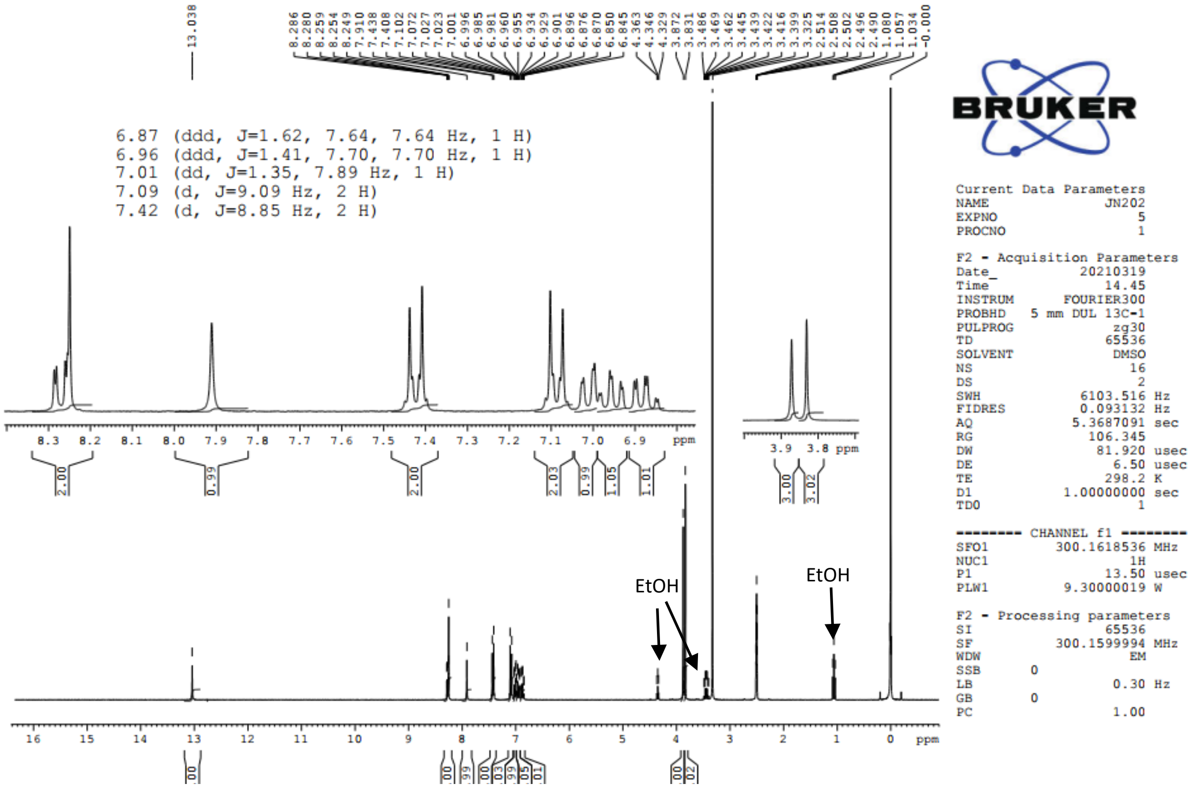

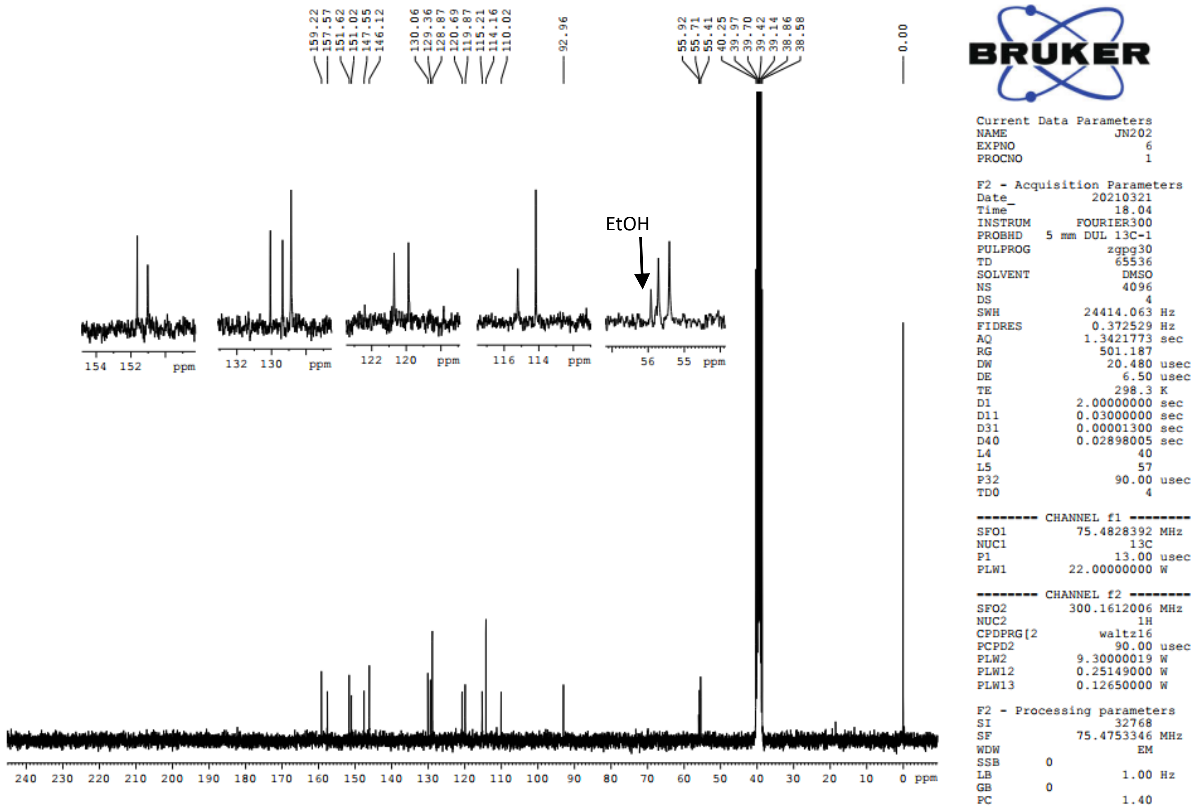

3-(4-Methoxylphenylamino)-5-(4-methoxylphenyl)-1,5-dihydro-4*H*-pyrazolo[3,4-*d*]pyrimidin-4-one (4t)

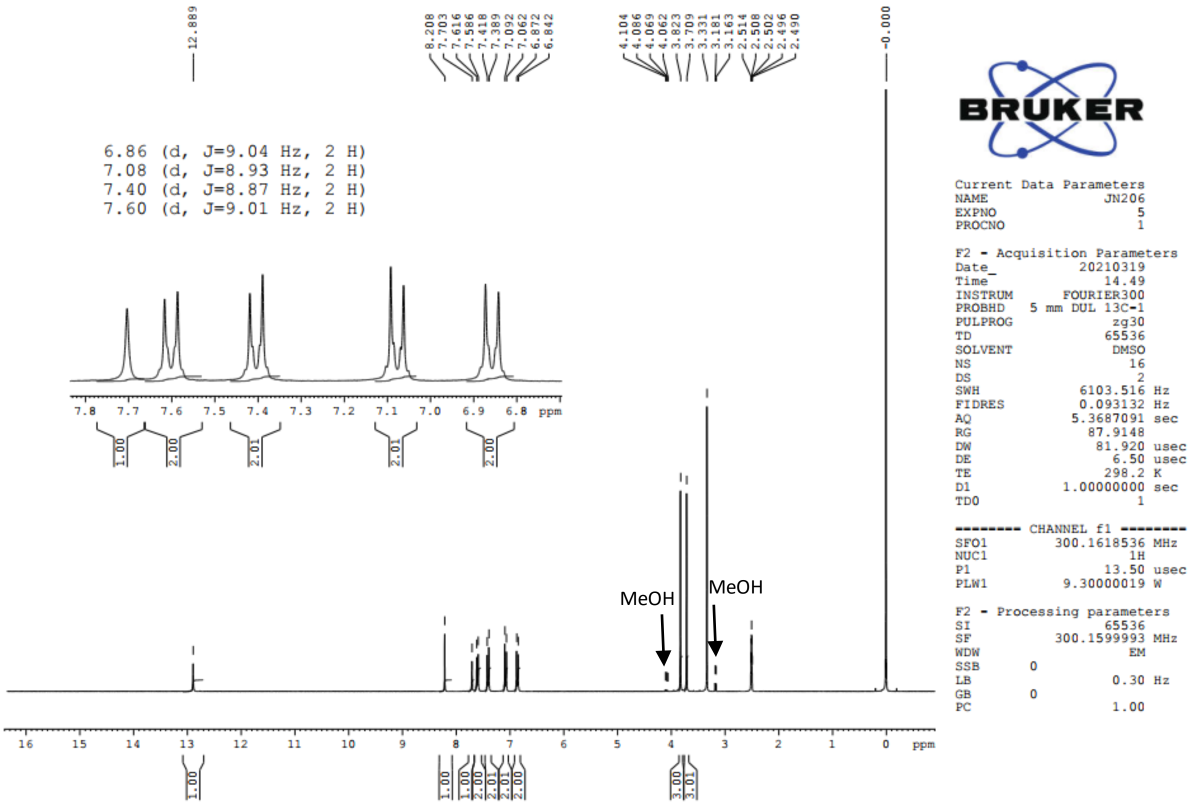

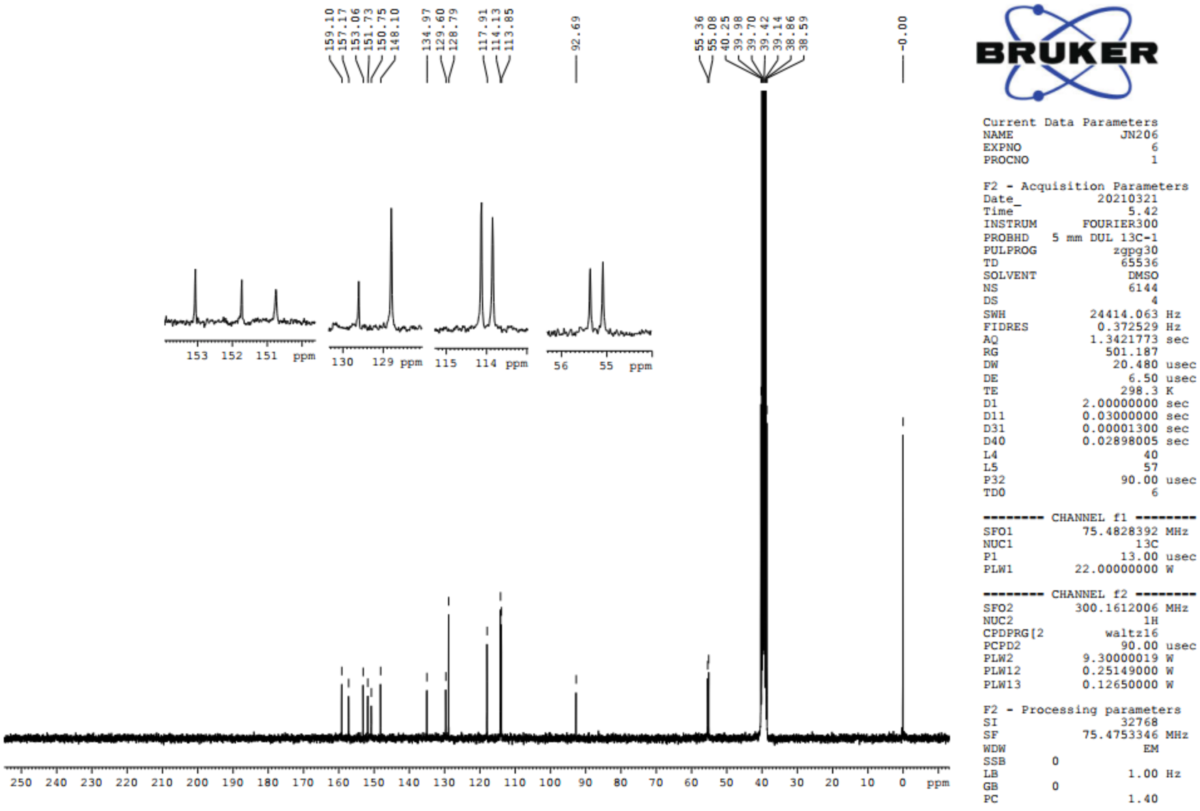

Supplement: RA-012-D2RA00980C-s003 [file RA-012-D2RA00980C-s003.pdf]
